# Association and linkage mapping to unravel genetic architecture of phenological traits and lateral bearing in Persian walnut (*Juglans regia* L.)

**DOI:** 10.1186/s12864-020-6616-y

**Published:** 2020-03-04

**Authors:** Anthony Bernard, Annarita Marrano, Armel Donkpegan, Patrick J. Brown, Charles A. Leslie, David B. Neale, Fabrice Lheureux, Elisabeth Dirlewanger

**Affiliations:** 1 INRAE, Univ. Bordeaux, UMR BFP, F-33882 Villenave d’Ornon, France; 2 CTIFL, centre opérationnel de Lanxade, 24130 Prigonrieux, France; 3 Department of Plant Sciences, University of California, Davis, CA 95616 USA

**Keywords:** Walnut, *Juglans regia* L., Association genetics, GWAS, Germplasm collection, Linkage map, QTL analysis, Phenology, Bearing habit

## Abstract

**Background:**

Unravelling the genetic architecture of agronomic traits in walnut such as budbreak date and bearing habit, is crucial for climate change adaptation and yield improvement. A Genome-Wide Association Study (GWAS) using multi-locus models was conducted in a panel of 170 walnut accessions genotyped using the Axiom™ *J. regia* 700 K SNP array, with phenological data from 2018, 2019 and legacy data. These accessions come from the INRAE walnut germplasm collection which is the result of important prospecting work performed in many countries around the world. In parallel, an F_1_ progeny of 78 individuals segregating for phenology-related traits, was genotyped with the same array and phenotyped for the same traits, to construct linkage maps and perform Quantitative Trait Loci (QTLs) detection.

**Results:**

Using GWAS, we found strong associations of SNPs located at the beginning of chromosome 1 with both budbreak and female flowering dates. These findings were supported by QTLs detected in the same genomic region. Highly significant associated SNPs were also detected using GWAS for heterodichogamy and lateral bearing habit, both on chromosome 11. We developed a Kompetitive Allele Specific PCR (KASP) marker for budbreak date in walnut, and validated it using plant material from the Walnut Improvement Program of the University of California, Davis, demonstrating its effectiveness for marker-assisted selection in Persian walnut. We found several candidate genes involved in flowering events in walnut, including a gene related to heterodichogamy encoding a sugar catabolism enzyme and a cell division related gene linked to female flowering date.

**Conclusions:**

This study enhances knowledge of the genetic architecture of important agronomic traits related to male and female flowering processes and lateral bearing in walnut. The new marker available for budbreak date, one of the most important traits for good fruiting, will facilitate the selection and development of new walnut cultivars suitable for specific climates.

## Background

Persian walnut (*Juglans regia* L.) is one of the oldest food sources known [1]. It is a monoecious and dichogamous tree species with 2n = 2x = 32 chromosomes [2], and grows in temperate regions [3]. Worldwide in-shell walnut production, mainly from China, California and Iran, exceeded 3800 kt in 2017, as reported by the Food and Agriculture Organization of the United Nations (www.fao.org). At more than 22,000 ha, Persian walnut is the second leading tree crop in France, after apple. In the last 3 years, France has oscillated between 7th and 9th position for in-shell walnut production (circa 40 kt) [4]. Increased yield, larger nut size, light kernel color, and ease of cracking are among the main goals of walnut breeding worldwide [5]. The ability to adapt to specific climatic conditions is also a breeding priority, especially in France where late spring frosts are prevalent [4]. In that respect, a better understanding of phenology and bearing habit, both key determinants of yield, is of upmost importance for walnut genetic improvement and cultivation [6].

Climate change, particularly global warming, is no longer to be proven within the scientific community [7], and researchers are studying its impact on phenology of temperate trees. In these species, growth is punctuated by an annually repeated phase of rest, called bud dormancy [8]. This dormancy period is influenced by various environmental factors, such as photoperiod and temperature, resulting in fulfilment of chilling and heat requirements [9]. In walnut, chilling and heat requirements were widely estimated in Iran [10] and showed, for instance, a range of chilling requirements from 650 h at + 4 °C for ‘Serr’ to 1000 h for ‘Hartley’ cultivars [11]. In France, the frost resistance of walnut were studied [12, 13] and many phenology studies of temperate tree species in Europe report a time shift of phenological events [14–17]. An advancing effect of warm springs on phenological events has been observed for walnut in California, particularly for leafing date [18]. Similar findings have been reported in Slovenia [19] and Romania [20]. Using phenological data recorded by the Institut National de Recherche pour l’Agriculture, l’Alimentation et l’Environnement (INRAE) of Bordeaux from 1989 to 2016, we also observed an average advance in budbreak in France of 5 days over the last 3 decades [21]. In Iran, researchers assessed land suitability for walnut cultivation under present and future climatic conditions, and predict that the currently suitable area will be significantly reduced [22].

Genetic control of phenology-related traits is fundamental for the development of new, resilient cultivars, able to adapt to changing climatic conditions. Many studies have focused on genetic dissection of phenological traits (e.g., chilling requirements and flowering time) in diverse fruit crops, such as peach, apricot and sweet cherry [23, 24]. In walnut, a significant genotype effect has been identified for heat requirements [25]. Moreover, high heritability has been shown for leafing date (71–96%), type of heterodichogamy (90%), and female/male blooming (80%) [26, 27]. Persian walnut has two main types of bearing habit. Fruiting can occur only at the terminal position of new branches or at both terminal and lateral positions [28]. A genetic locus for lateral bearing has been identified based in an F_2_ progeny in the United-States [29], but has not been sufficiently robust for wider use in marker-assisted selection.

Release of the first walnut genome sequence [30] facilitated advanced genetic and genomic studies, including development of the first high-density Axiom™ *J. regia* 700 K SNP genotyping array [31]. Application of this powerful genotyping tool allowed genetic dissection of crucial traits in walnut, such as nut-related traits [32] and water use efficiency [33, 34]. A recent study, combining genome-wide association study (GWAS) and classical linkage mapping, found major loci for leafing and harvest dates on chromosome 1 (Chr1), and lateral fruitfulness on Chr11 [35].

Here, we studied for the first time in walnut, the genetic control of budbreak date and female/male flowering dates, using the Axiom™ *J. regia* 700 K SNP array to genotype both a panel of 170 walnut accessions of diverse geographical provenience and an F_1_ progeny segregating for these traits. This study sought to identify candidate genes for both female and male flowering dates and to develop the first Kompetitive Allele Specific PCR (KASP) marker for phenology in walnut. This will be useful for walnut breeding programs in selecting of new resilient varieties to climate change.

## Results

### Phenotypic variations of phenology-related traits and lateral bearing

Two populations were used in this study: a GWAS panel of 170 diverse accessions of worldwide origin and an F_1_ mapping progeny of 78 individuals resulting from a bi-parental controlled cross between ‘Franquette’ (late flowering), and ‘UK 6–2’ (intermediate to early flowering). Both populations were maintained at the INRAE of Bordeaux field station and phenotyped during 2018 and 2019. For the GWAS panel, we also used previously collected (legacy) phenotypical data taken between 1989 and 2011.

For the GWAS panel, the 2018–2019 data exhibited high variation in phenology-related traits, particularly for budbreak which ranged in 2019 from 57 Julian days for ‘Early Ehrhardt’ to 128 for ‘Fertignac’ (Feb 27th to May 9th) (Figures S1 and S2). The F_1_ progeny in 2019 exhibited a smaller range of 76 to 102 Julian days (Figure S3 and S4). Generally, budbreak was earlier in 2019 (87.78 Julian days ±12.65 for the GWAS panel, 90.71 ± 5.48 for the F_1_ progeny) than in 2018 (92.47 ± 11.06 for the GWAS panel, 95.55 ± 4.97 for the F_1_ progeny).

**Fig. 1:**
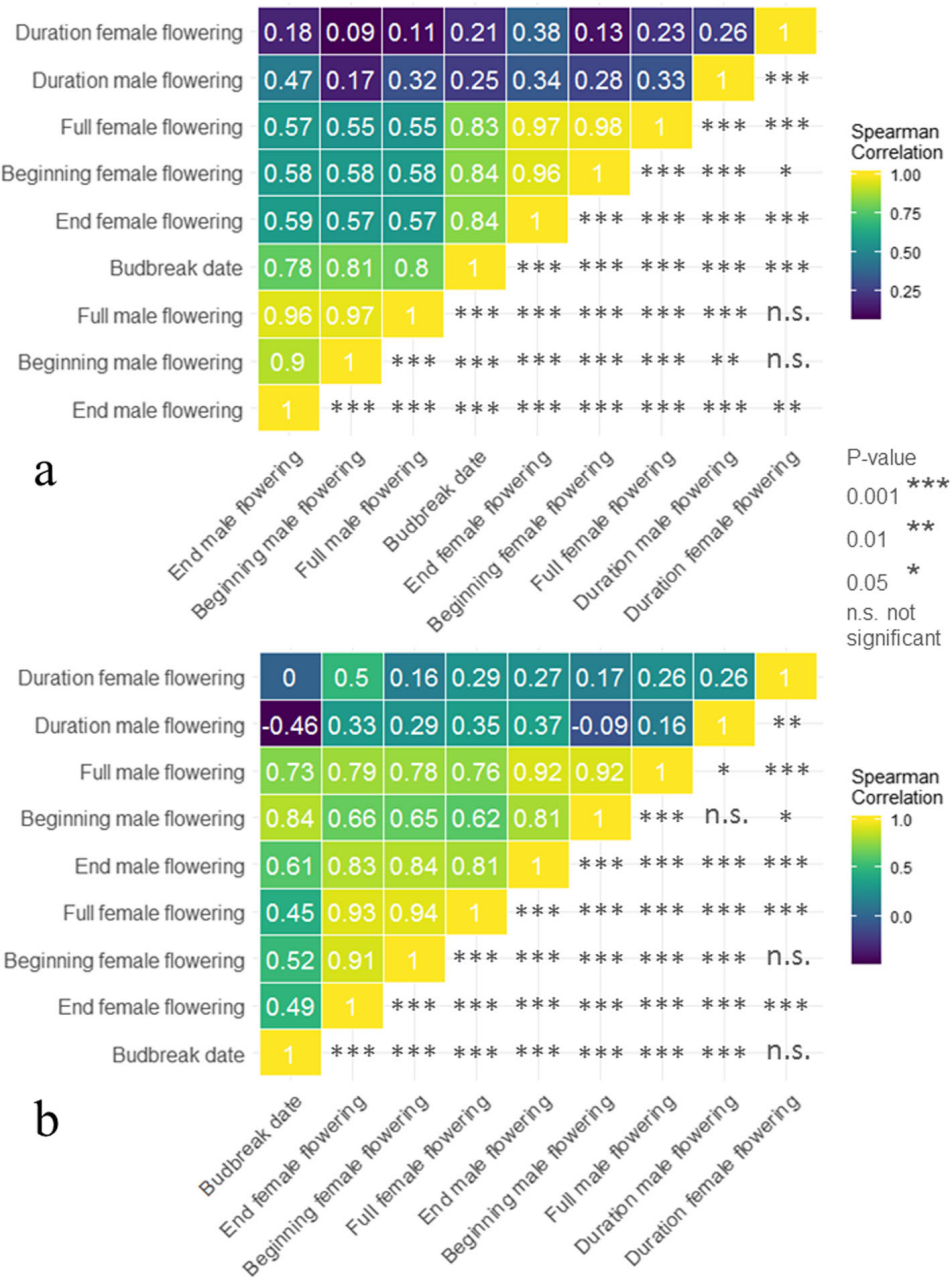
Correlation matrices of the traits using two-year data. **a** Using the GWAS panel, and **b** using the F_1_ progeny

We found significant positive correlations between budbreak date and female flowering stages for both the GWAS panel (0.83 to 0.84; Fig. 1a), and the F_1_ progeny (0.45 to 0.52; Fig. 1b). Similar significant positive correlations were found between budbreak date and male flowering stages for the GWAS panel (0.78 and 0.81; Fig. 1a), and the F_1_ progeny (0.61 to 0.84; Fig. 1b). Comparison of the 2 years shows that early accessions in 2018 were also early in 2019, suggesting genetic control of phenology-related traits in walnut. Female flowering was earlier in 2018 than 2019, but the accession order was consistent for both years. In addition, both female and male flowering durations showed low correlations and low statistical significances with other traits. We did not phenotype the F_1_ progeny for bearing habit, since this trait did not segregate in that population, but we observed great variability for fruit bearing within the GWAS panel.

High broad-sense heritability values were observed for budbreak date, with H^2^ of 0.95 when using legacy data and 0.93 using only two-year data (Table 1). Overall, H^2^ values were lower within the F_1_ progeny (H^2^ = 0.67 for budbreak date). However, we found low values for male flowering duration (H^2^ = 0.22) and female flowering duration (H^2^ = 0.26) within GWAS panel (using the recent two phenotyping years), while no genetic effect was found for the F_1_ progeny. Therefore, we did not consider both male and female flowering durations in the GWAS and QTL mapping analyses.

**Table 1:** Descriptive statistics and broad-sense heritabilities

Trait	Plant material	Year	Mean^**a**^ ± SD^**b**^	Range^**a**^	H^**2**^
Bearing habit	GWAS panel	1989–2016	4.01 ± 2.71	1–9	–
		2018	–	–	–
		2019	4.61 ± 2.18	1–9	–
Budbreak date	GWAS panel	1989–2016	99.02 ± 12.87	60–133	0.95
		2018	92.47 ± 11.06	72–115	0.93
		2019	87.78 ± 12.65	57–128	
	F_1_ progeny	2018	95.55 ± 4.97	90–105	0.67
		2019	90.71 ± 5.48	76–102	
Beginning female flowering date	GWAS panel	1989–2016	119.11 ± 11.71	69–151	0.91
		2018	111.27 ± 10.19	90–142	0.95
		2019	110.70 ± 13.28	78–141	
	F_1_ progeny	2018	112.54 ± 5.12	106–124	0.75
		2019	116.38 ± 5.03	102–128	
Peak female flowering date	GWAS panel	1989–2016	125.11 ± 11.47	78–154	0.93
		2018	115.22 ± 11.42	95–147	0.96
		2019	115.42 ± 13.00	87–144	
	F_1_ progeny	2018	116.69 ± 5.63	110–128	0.67
		2019	121.81 ± 5.28	110–132	
End female flowering date	GWAS panel	1989–2016	135.14 ± 12.09	88–167	0.90
		2018	122.32 ± 12.35	103–153	0.96
		2019	122.38 ± 12.86	97–149	
	F_1_ progeny	2018	123.35 ± 6.27	112–135	0.64
		2019	128.42 ± 5.44	116–137	
Female bloom duration	GWAS panel	1989–2016	16.47 ± 6.66	1–53	0.37
		2018	11.05 ± 4.17	3–23	0.26
		2019	11.68 ± 2.68	5–19	
	F_1_ progeny	2018	10.81 ± 3.11	4–16	0.00
		2019	12.04 ± 2.30	6–17	
Heterodichogamy	GWAS panel	1989–2016	2.80 ± 2.09	1–9	0.95
		2018	3.90 ± 2.15	1–9	0.84
		2019	3.17 ± 2.48	1–9	
Beginning male flowering date	GWAS panel	1989–2016	112.34 ± 10.69	77–149	0.82
		2018	108.17 ± 6.81	99–137	0.86
		2019	105.06 ± 10.68	85–140	
	F_1_ progeny	2018	106.17 ± 3.25	102–114	0.75
		2019	104.17 ± 5.25	88–116	
Peak male flowering date	GWAS panel	1989–2016	116.99 ± 10.64	83–154	0.88
		2018	111.13 ± 8.08	103–142	0.92
		2019	109.09 ± 10.94	91–144	
	F_1_ progeny	2018	108.38 ± 3.89	104–117	0.86
		2019	108.56 ± 5.69	95–128	
End male flowering date	GWAS panel	1989–2016	122.45 ± 10.58	85–163	0.87
		2018	114.40 ± 9.62	104–145	0.95
		2019	114.33 ± 11.13	97–149	
	F_1_ progeny	2018	111.97 ± 4.86	105–123	0.81
		2019	114.74 ± 6.17	102–130	
Male bloom duration	GWAS panel	1989–2016	10.53 ± 4.70	2–35	0.32
		2018	6.23 ± 3.97	2–24	0.22
		2019	9.27 ± 2.45	4–16	
	F_1_ progeny	2018	5.81 ± 2.20	2–13	0.00
		2019	10.58 ± 3.16	6–21	

^a^ Date and duration traits are in Julian days, bearing habit and heterodichogamy are categorical traits from 1 to 9

^b^ SD is the abbreviation for standard deviation

### Population structure of the GWAS panel

**Fig. 2:**
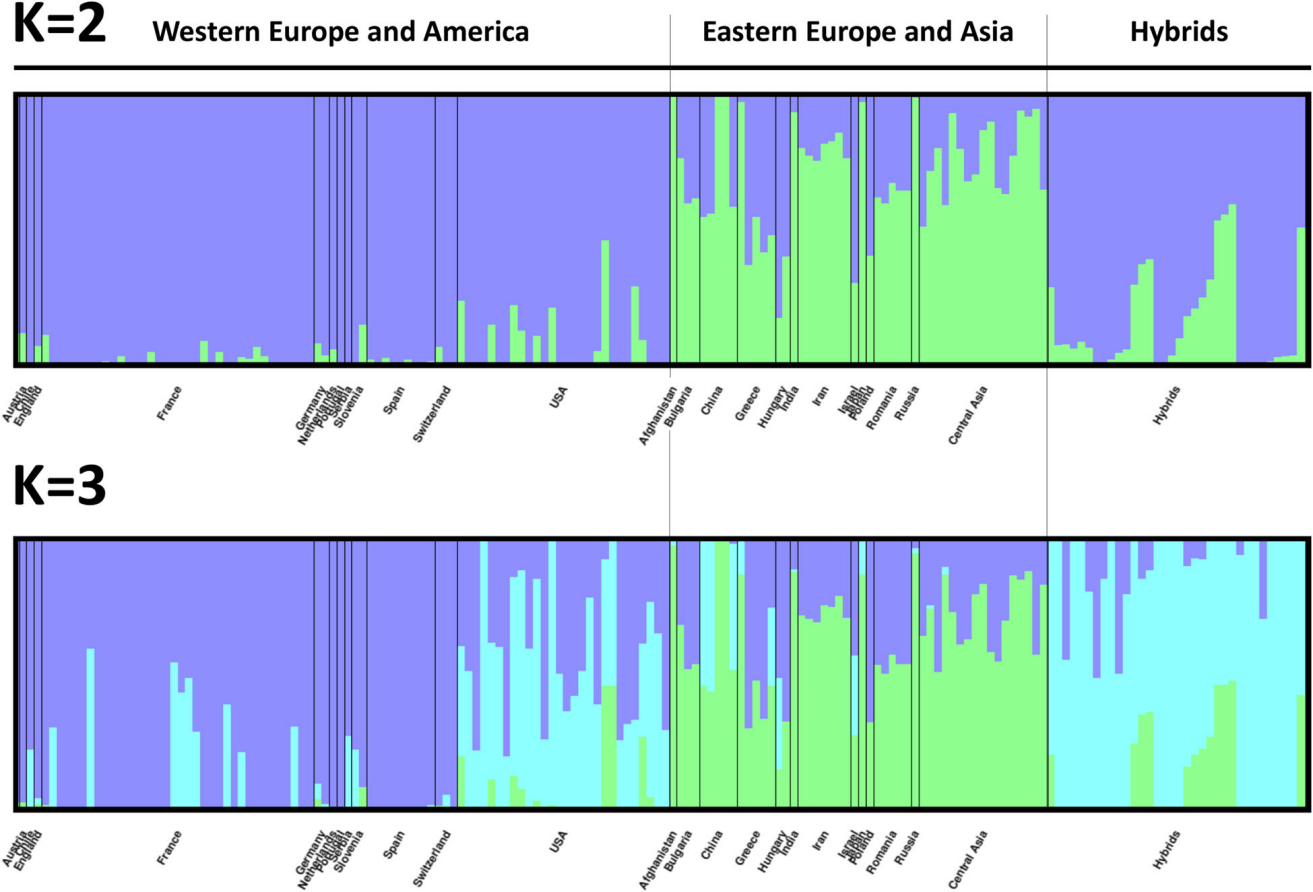
Structure of the GWAS panel. The fastSTRUCTURE software was used. Bar plot of individual ancestry proportions (Q values) for the genetic cluster inferred using the whole set of 364,275 robust SNPs. For K = 2, accessions are geographically separated in two main groups: the purple group for ‘Western Europe and America’ accessions, and the green group for ‘Eastern Europe and Asia’ accessions. For K = 3, the blue group, highlights hybrids

A total of 364,275 SNPs were retained after filtering for high resolution SNPs categories (Poly High Resolution and No Minor Homozygotes), for genotyping rate > 90%, and minor allele frequency > 5% (Table 2). We investigated the population structure of our association panel using the Bayesian clustering approach implemented in fastSTRUCTURE, and Principal Component Analysis (PCA). The fastSTRUCTURE analysis infers accession ancestry from genotypic information and permitted us to determine the best number of clusters (K). The most likely K subpopulations were K = 2 and K = 3 (Figure S5). At K = 2, admixture proportions clustered the accessions according to their geographical origin. In particular, the cluster in purple named “Western Europe and America” includes 86 accessions from Austria, Chile, England, France, Germany, Netherlands, Portugal, Serbia, Slovenia, Spain, Switzerland and USA. The cluster in green named “Eastern Europe and Asia” includes 50 accessions from Afghanistan, Bulgaria, China, Greece, Hungary, India, Iran, Israel, Japan, Poland, Romania, Russia and Central Asia (Fig. 2). At K = 3, a new cluster includes all the hybrids and admixed accessions from France and USA (Fig. 2, Table S1).

**Table 2:** SNPs used for the GWAS analyses and the construction of the parental linkage maps ‘Franquette’ and ‘UK6–2’

	**Number of markers**		**Percentage of markers**	
*Total of SNPs*	*609,658*		*100*	
**To keep SNPs of high resolution from Axiom® Analysis Suite**				
High resolution SNPs				
PolyHighResolution	397,921		65,27	
NoMinorHom	75,564		12,39	
MonoHighResolution	36,684		6,02	
Low resolution SNPs				
CallRateBelowThreshold	27,761		4,55	
OffTargetVariant	4787		0,79	
Other	66,941		10,98	
*Total of retained SNPs*	*510,169*		*83.68*	
**To keep SNPs with mendelian inheritance using F**_**1**_ **progeny**				
SNPs having no mendelian inheritance	661			
*Total of retained SNPs*	*509,508*		*83.57*	
**To keep SNPs having genotyping rate > 90%**				
	**GWAS**		**Linkage maps**	
	**Number of markers**	**Percentage of markers**	**Number of markers**	**Percentage of markers**
SNPs having genotyping rate < 90%	13,993		31,050	
*Total of retained SNPs*	*495,515*	*81.28*	*478,458*	*78,48*
**To keep SNPs having minor allele frequency > 5%**				
SNPs having minor allele frequency < 5%	123,751		–	
*Total of retained SNPs*	*371,764*	*60.98*	–	–
**To delete homozygote markers within parents**				
Homozygote markers	–		264,623	
*Total of retained SNPs*	–	–	*213,835*	*35.07*
**To delete same heterozygote markers within parents**				
Same heterozygote markers	–		40,860	
*Total of retained SNPs*	–	–	*172,975*	*28.37*
**To delete redundant SNPs in the genome**				
Redundant SNPs	7489		10,857	
*Total of retained SNPs*	*364,275*	*59.75*	*162,118*	*26.59*
**To delete distorded and identical markers**				
Distorded and identical markers	–		160,181	
*Total of retained SNPs*	–	–	*1937*	*0.32*
			*‘Franquette’ map: 849*	
			*‘UK 6–2’ map: 1088*

PCA shows similar clustering of our germplasm collection as fastSTRUCTURE (Figure S6). PC1, which explains 7.37% of total variance, separated the “Western Europe and America” (WEAm) accessions from the “Eastern Europe and Asia” (EEAs) accessions. PC2 accounted for 5.80% of variance explained and separated the hybrids and admixed accessions from France and USA, observed with K = 3 in fastSTRUCTURE.

### Relatedness of the GWAS panel

In addition to population structure, we investigated the familial relatedness within our association panel by estimating kinship coefficient (k) with the KING method. To identify first-degree relationships and differentiate “parent-offspring” from “full sibling” pairs, we used the estimates of k and the proportion of zero identical-by-state (IBS0) observed in the F_1_ progeny (Figure S7). In particular, we defined all pairwise relationships in the GWAS panel with k > 0.17 and 0 < IBS0 <  0.019 to be parent-offspring relationships. Results confirmed known pedigrees, particularly for the hybrids accessions and the modern cultivars from France and the USA. We also identified new relationships, such as that between ‘Grosvert n°1’ and ‘Verdelet’, French landraces from the departments of Dordogne and Corrèze, which may be full-sibs (Figure S8). Moreover, ‘Ashley’ and ‘Payne’, said to be identical, show the highest kinship coefficient.

### Genome-wide analysis for bearing H abit

**Fig. 3:**
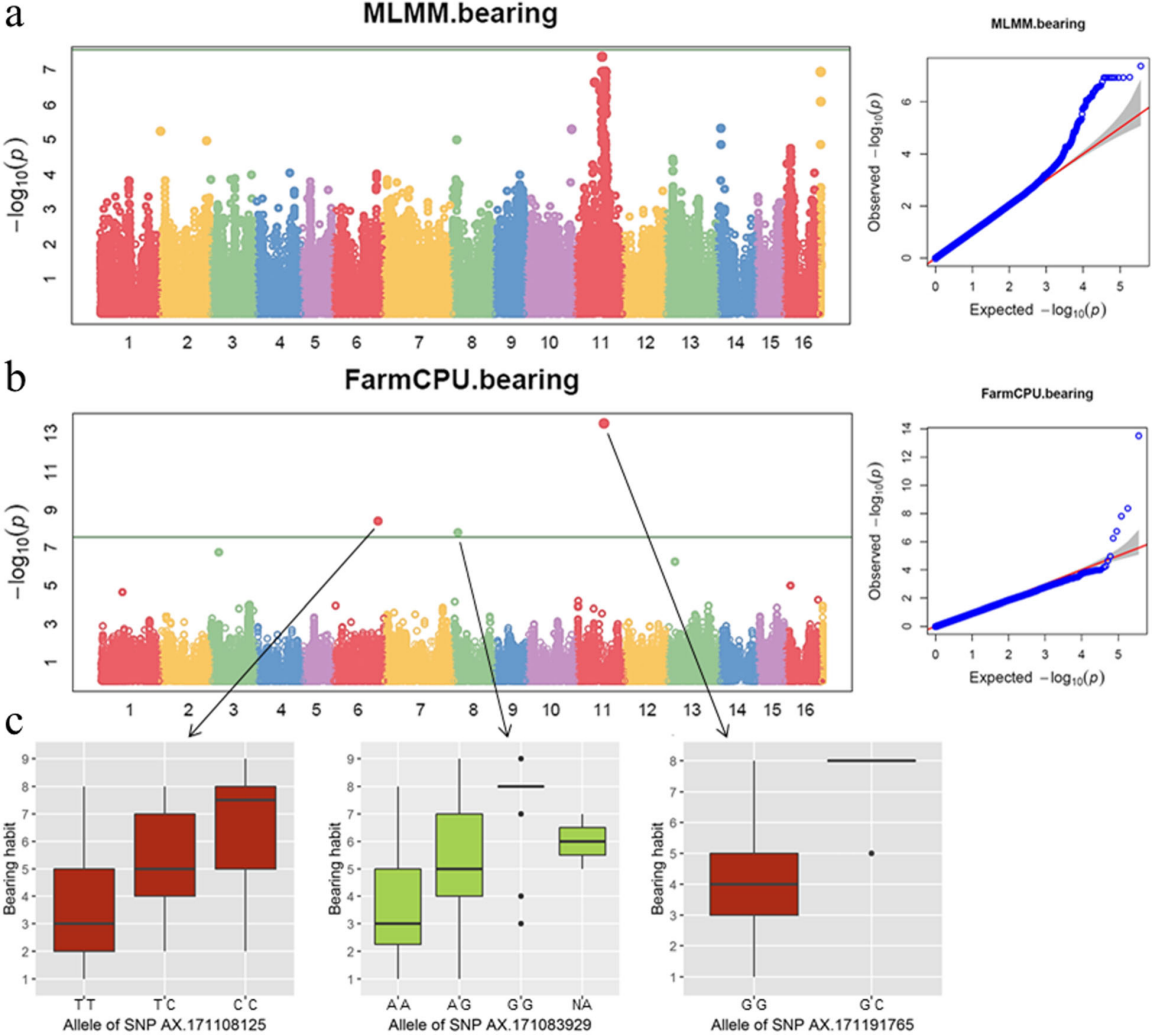
GWAS results for bearing habit using 2019 data. Manhattan plots followed by Q-Q plots using **a**) MLMM model, **b**) FarmCPU model, and **c**) box plots of the allele effects for the 3 SNPs associated with bearing habit

For bearing habit, we found no influence of population structure (PC = 0 according to the ‘model selection’ function implemented in GAPIT; Table S2). We used multi-locus mixed model (MLMM), and Fixed and random model Circulating Probability Unification method (FarmCPU). GWAS results using both models showed a significant association on Chr11 with bearing habit, using only the 2019 data (Fig. 3). The most significantly associated marker was the SNP ‘AX-171191765’ (physical position: 20,831,267 bp; *p*-value: 2.98E-14), and two additional associations are also found on Chr6 (SNP ‘AX-171108125’; p-value = 4.08E-09) and Chr8 (SNP ‘AX-171083929’; p-value = 1.47E-08), according to the false discovery rate (FDR) threshold (≥ 0.05).

The boxplots show the bearing habit phenotypes of 2019 for the different alleles of the three associated SNPs (Fig. 3). For the most significantly associated SNP ‘AX-171191765’, the allele G is linked to a terminal bearing habit, whereas the allele C is linked to a lateral bearing habit (R^2^ = 34.3%, allelic estimated effect = 2.59), leading to an increased yield.

### Association and linkage mapping for Budbreak date and female flowering dates

**Fig. 4:**
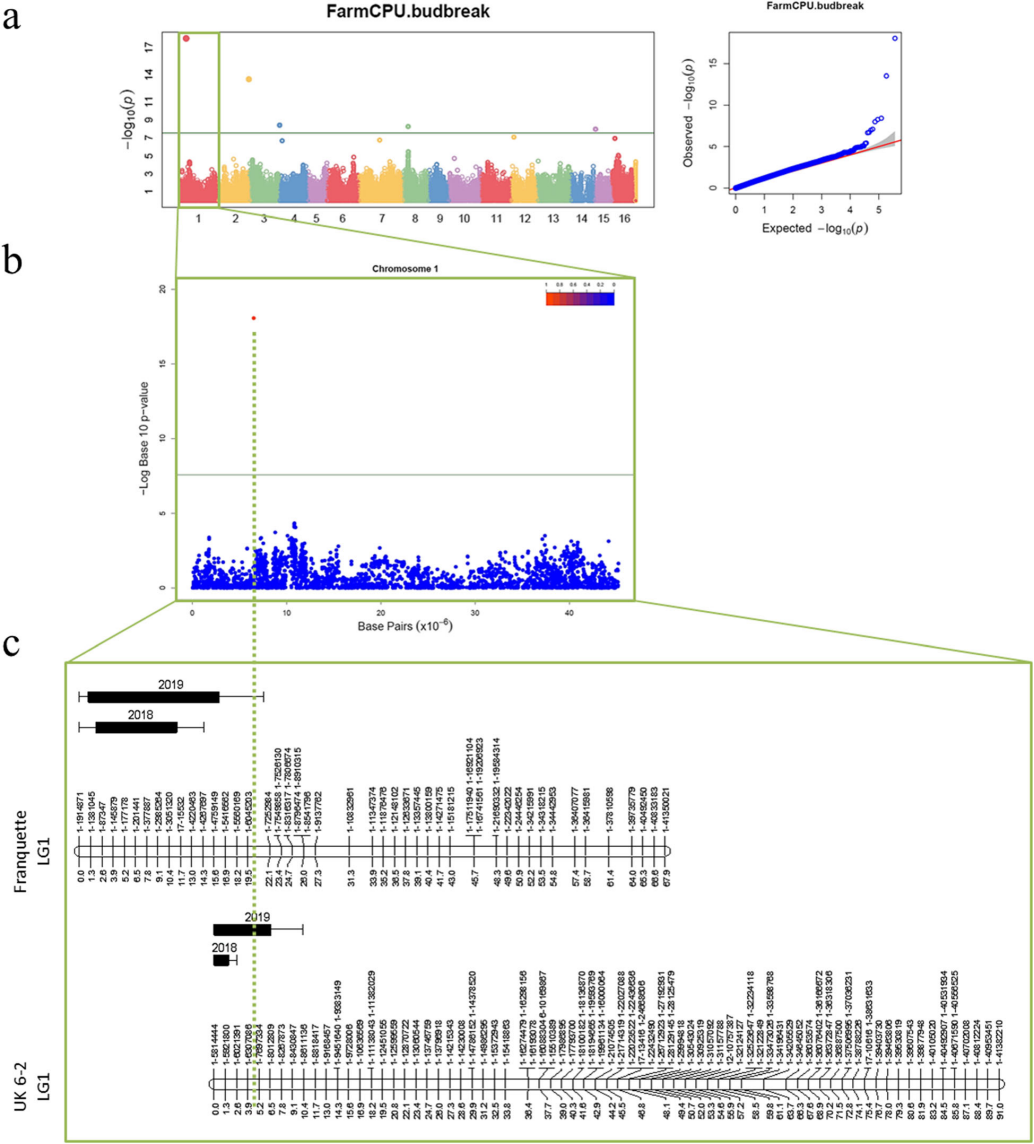
GWAS and linkage mapping results for budbreak date. **a** Manhattan plot followed by Q-Q plots using BLUPs with two-year data and FarmCPU model, **b** focus on chromosome 1, and **c**) QTLs found using 2018 and 2019 data and the F_1_ progeny. The dotted green line indicates the physical position (6,514,832 bp) of the SNP found in GWAS transposed into the linkage maps

**Fig. 5:**
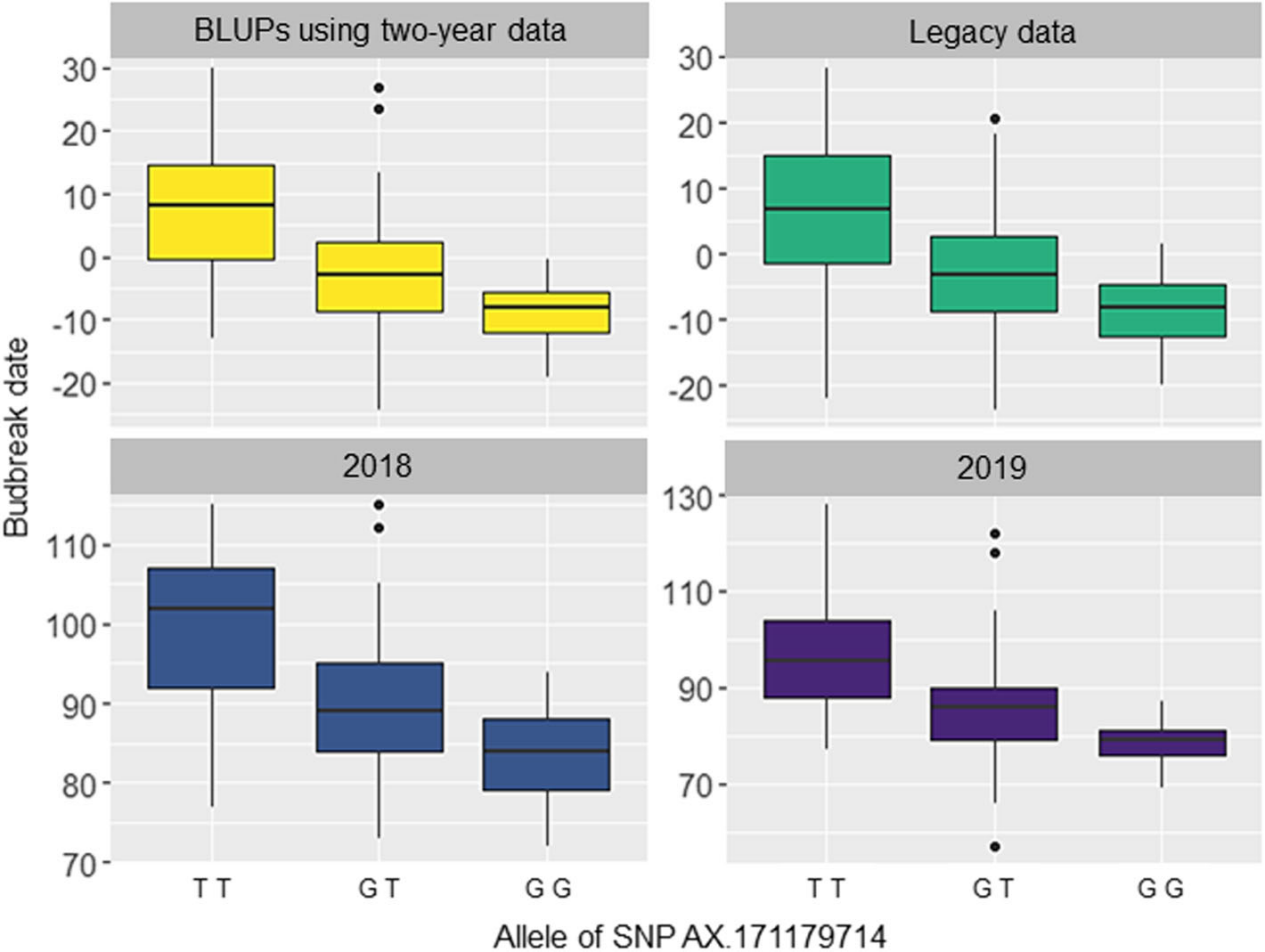
Box plots of the allele effects for the SNP AX-171179714 associated with budbreak date. FarmCPU model was used and all datasets: BLUPs using two-year data, legacy data, 2018 data and 2019 data

Using 1937 SNPs (Table 2), the ‘Franquette’ and ‘UK 6–2’ parental genetic maps constructed have a length of 1015 and 1346 cM, and a number of markers of 849 and 1088 SNPs, respectively (Table S3). The marker names of the genetic maps were changed with the corresponding chromosome number and its physical position for a better visualization (Figure S9). For all the phenology-related traits, we also found that population structure did not influence phenology in our GWAS panel. Both GWAS and classical QTL mapping identified marker-trait associations for budbreak date in the same region on Chr1 (Fig. 4). The most significant associated SNP ‘AX-171179714’ on the Chr1 (physical position: 6,514,832 bp) was found using the Best Linear Unbiased Predictions (BLUPs) of two-year data and co-localizes with the major QTLs identified for both parents in 2019 using the F_1_ progeny data. The allele T of this SNP is linked to a late budbreak date (R^2^ = 30.6%, allelic estimated effect = 5.9) (Table 3). We also ran a Kruskal-Wallis test to find if the phenotypic differences were significant among the three genotypes using the different phenotypic datasets, and this allelic effect remains consistent (*p*-values = 1.84E-13 for two-year data, 6.63E-12 for 2018, 9.76E-13 for 2019, and 2.61E-09 for legacy data; Fig. 5). The co-localizing major QTLs found in 2019 in LG 1 in ‘Franquette’ and ‘UK 6–2’ explain 23.9 and 34.8% of the budbreak date variance, respectively. In addition, GWAS with two-year data found four additional associations on chromosomes 2, 4, 8 and 15, while the classical linkage mapping analysis identified minor QTLs on linkage groups 6, 11, 12 and 14.

**Table 3:** Summary of association and linkage mapping results related to bearing habit, budbreak date and female flowering dates

Dataset	GWAS using FarmCPU model						QTLs detection using MIM method						
	SNP	Chr ^**a**^	Physical position	Significance ^**b**^	R^**2 c**^	Alleles/Effect ^**d**^	Peak SNP ^**e**^	LG ^**f**^	Physical position	Confidence interval ^**g**^	LOD ^**h**^	P.E.V. ^**i**^	d ^**j**^
**Bearing habit**													
2019	AX-171108125	6	33,909,308	4.08E-09	30.8	C/T (0.7)							
	AX-171083929	8	3,102,647	1.47E-08	32.5	A/G (−0.8)							
	AX-171191765	11	20,831,267	2.98E-14	34.3	C/G (2.6)							
**Budbreak date**													
1989–2016	AX-171509395	5	21,137,700	1.38E-10	9.0	C/T (4.1)							
	AX-171490851	7	37,022,231	5.76E-11	35.0	C/G (−4.9)							
	AX-170810238	8	23,687,589	7.78E-12	25.6	C/T (−4.1)							
2018–2019	AX-171179714	1	6,514,832	8.95E-19	30.6	G/T (−5.9)	AX-171180038	F1	177,178	2.8–10.8	11.9	23.7	5.2
	AX-171557178	2	36,586,423	2.84E-14	27.3	A/T (−4.7)	AX-170771092	U1	5,814,444	0.0–2.8	29.5	44.3	−6.9
	AX-171040881	4	591,186	3.70E-09	4.8	G/T (−3.2)	AX-171486072	U6	2,467,998	0.0–46.7	6.5	6.7	−2.6
	AX-171522523	8	5,949,933	5.41E-09	3.2	A/T (−2.6)	AX-170627138	U11	26,651,484	0.0–30.2	4.7	4.7	−2.0
	AX-171509630	15	1,111,543	9.92E-09	6.8	A/G (2.6)	AX-170901703	U12	19,779,190	39.0–67.4	6.8	7.7	2.8
							AX-170818106	F14	9,871,222	3.6–50.0	6.0	11.2	3.5
2018	AX-171520811	1	6,537,230	2.54E-10	29.1	A/G (4.3)	AX-171180038	F1	177,178	0.0–14.4	6.3	31.0	5.4
	AX-171557178	2	36,586,423	4.31E-09	25.1	A/T (−3.4)	AX-170771092	U1	5,814,444	0.0–2.6	15.4	50.2	−7.0
	AX-170682386	15	1,162,635	1.05E-08	18.8	A/G (3.6)	AX-170760959	U6	2,438,026	0.0–61.4	3.4	7.9	−2.6
	AX-170691861	16	3,791,224	1.47E-10	25.5	C/T (−4.3)							
2019	AX-171557178	2	36,586,423	2.70E-11	26.2	A/T (−4.6)	AX-171180038	F1	177,178	0.0–21.4	5.6	23.9	5.4
	AX-171489741	4	3,486,412	8.12E-10	26.2	C/G (−4.8)	AX-170771092	U1	5,814,444	0.0–10.3	11.1	34.8	−6.7
	AX-171139350	6	31,522,110	6.17E-09	< 0.1	C/T (3.6)	AX-170901703	U12	19,779,190	27.8–76.4	3.7	9.2	3.3
	AX-170938460	7	22,604,203	8.50E-13	26.8	A/G (5.0)							
	AX-171047138	10	36,604,822	7.89E-09	0.1	A/G (4.5)							
	AX-170691931	16	3,762,646	2.69E-10	24.0	A/G (−4.9)							
**Beginning female flowering date**													
2018–2019	AX-170990138	1	9,298,520	1.56E-09	35.0	A/G (−3.4)	AX-171521263	F1	201,441	3.4–10.2	12.9	30.6	5.2
	AX-170938895	7	23,018,189	6.62E-13	31.4	A/C (4.0)	AX-170771092	U1	5,814,444	< 0.5	35.1	46.5	−7.1
							AX-170684860	U3	2,653,247	0.0–105.6	5.0	2.6	1.9
							AX-171583903	U6	2,237,730	0.0–82.9	5.9	4.1	−2.1
							AX-170825127	U11	25,463,971	0.0–8.3	9.3	6.5	−2.8
							AX-170901663	U12	19,737,912	44.5–60.8	8.1	6.9	2.7
							AX-170818106	F14	9,871,222	0.0–60.5	4.3	3.8	2.5
							AX-170786852	U16	20,462,383	21.7–77.2	5.1	3.9	2.0
2018	AX-170990138	1	9,298,520	1.79E-11	35.4	A/G (−4.2)	AX-171180038	F1	177,178	0.0–14.2	6.6	32.2	5.7
	AX-171029453	4	4,123,643	7.38E-13	23.6	C/T (−4.9)	AX-170771092	U1	5,814,444	0.0–3.1	14.6	47.6	−7.2
2019	AX-170990138	1	9,298,520	3.33E-13	34.8	A/G (−4.5)	AX-171180038	F1	177,178	0.0–16.4	5.6	21.6	4.7
	AX-171029453	4	4,123,643	1.55E-10	25.6	C/T (−4.2)	AX-170771092	U1	5,814,444	0.0–2.3	23.0	44.4	−7.6
	AX-170938895	7	23,018,189	2.99E-13	35.1	A/C (5.1)	AX-170684860	U3	2,653,247	0.0–23.8	5.3	5.8	2.7
	AX-170586625	16	626,899	2.92E-12	2.6	C/T (−4.2)	AX-171569583	U6	12,329,274	26.3–62.5	7.5	8.5	−3.3
							AX-170825127	U11	25,463,971	39.9–68.1	7.5	8.5	−3.2
							AX-170901663	U12	19,737,912	0.0–3.6	7.5	8.7	3.3
**Peak female flowering date**													
1989–2016	AX-170990138	1	9,298,520	8.15E-13	39.6	A/G (−3.7)							
	AX-171596225	6	14,925,533	1.39E-09	26.7	A/G (3.0)							
	AX-171029018	7	48,197,343	3.80E-09	0.1	A/C (2.2)							
	AX-171161427	7	37,022,706	9.52E-09	35.9	C/T (3.0)							
	AX-171093716	10	23,540,554	5.39E-09	8.3	A/G (−4.6)							
	AX-171553780	10	6,443,284	1.65E-08	0.4	A/T (2.2)							
	AX-171161986	13	3,292,458	2.11E-08	30.9	A/G (2.8)							
2018–2019	AX-170990138	1	9,298,520	4.75E-11	36.2	A/G (−4.5)	AX-171180038	F1	177,178	2.6–11.2	9.7	22.3	5.0
	AX-171204022	7	25,355,784	6.12E-09	31.2	A/G (−3.6)	AX-170771092	U1	5,814,444	0.0–1.9	42.5	49.0	−8.3
							AX-170869355	F2	35,198,358	18.9–67.9	5.2	7.4	−3.2
							AX-171121327	U3	1,942,419	0.0–33.7	6.4	4.3	2.3
							AX-171204628	U9	21,282,336	32.9–57.3	5.9	3.7	2.3
							AX-170627138	U11	26,651,484	0.0–7.3	13.1	7.7	−3.5
							AX-170901663	U12	19,737,912	37.3–65.2	10.0	6.9	3.0
2018	AX-170990138	1	9,298,520	1.41E-08	35.4	A/G (−3.6)	AX-171521263	F1	201,441	0.0–22.0	4.5	23.4	5.4
	AX-170975750	6	22,755,843	1.39E-08	< 0.1	A/G (−3.8)	AX-170771092	U1	5,814,444	0.0–4.9	13.7	45.5	−7.8
	AX-170676955	9	8,265,700	3.56E-09	6.7	A/G (−3.3)	AX-170697456	U4	5,261,727	0.0–49.2	3.3	8.5	3.0
							AX-170787652	U15	151,849	0.0–33.6	3.8	10.0	−3.5
2019	AX-170990138	1	9,298,520	2.70E-11	35.6	A/G (−4.4)	AX-171180038	F1	177,178	0.0–25.2	4.5	18.2	4.5
	AX-170938895	7	23,018,189	6.82E-15	36.7	A/C (5.1)	AX-170771092	U1	5,814,444	0.0–2.0	24.0	46.4	−7.7
							AX-170684716	U3	2,526,842	0.0–50.7	4.4	4.5	2.3
							AX-171569583	U6	12,329,274	0.0–98.6	3.9	3.8	−2.2
							AX-171222942	U8	5,077,987	0.0–98.8	3.8	3.8	2.1
							AX-171204628	U9	21,282,336	37.2–57.3	4.5	5.1	2.5
							AX-170582801	U11	26,090,506	0.0–7.3	9.4	11.0	−3.7
							AX-171033431	U12	17,150,374	34.4–70.9	5.1	5.3	2.6
							AX-170581901	U16	17,340,045	2.2–66.1	4.0	4.0	2.2
**End female flowering date**													
2018–2019	AX-170990138	1	9,298,520	8.81E-11	39.3	A/G (−4.1)	AX-171180038	F1	177,178	2.9–10.5	10.8	24.8	5.2
	AX-171103669	1	3,411,327	8.46E-10	28.0	A/C (3.5)	AX-170771092	U1	5,814,444	< 0.5	32.9	49.9	−8.2
	AX-171199539	15	16,085,753	2.06E-10	< 0.1	A/G (−3.4)	AX-170868251	F2	34,498,992	32.8–67.9	7.2	13.7	4.0
							AX-171121327	U3	1,942,419	0.0–41.2	6.3	3.6	2.7
							AX-170620438	U5	18,147,969	0.0–78.7	5.4	2.9	2.3
							AX-171214747	U9	18,177,182	14.3–57.3	5.0	5.0	2.5
							AX-170825127	U11	25,463,971	0.0–13.8	6.6	3.4	−2.7
							AX-170818106	F14	9,871,222	0.0–56.8	5.8	7.7	3.5
2018	AX-171572440	9	20,815,377	3.24E-09	1.3	A/G (−2.9)	AX-171521263	F1	201,441	2.6–9.8	6.6	25.4	6.4
							AX-170771092	U1	5,814,444	0.0–7.1	13.0	48.0	−8.7
							AX-170867988	F2	34,288,275	18.6–67.9	4.0	13.8	−4.4
2019	AX-170990138	1	9,298,520	6.75E-11	38.1	A/G (−4.3)	AX-170771092	U1	5,814,444	0.0–3.6	19.8	40.1	−7.6
	AX-171029453	4	4,123,643	1.12E-10	27.6	C/T (−4.9)	AX-171210974	U3	2,714,686	0.0–14.7	6.5	8.5	3.4
	AX-170938854	7	22,982,368	1.14E-12	37.0	C/T (4.5)	AX-170682524	U5	18,023,534	0.0–14.0	6.7	8.7	3.5
	AX-170766461	8	27,866,613	7.85E-09	< 0.1	A/C (−5.3)	AX-170626929	U11	26,817,955	2.3–12.5	7.0	9.1	−3.6
	AX-170860236	14	9,448,288	1.25E-10	< 0.1	G/T (4.3)	AX-170901663	U12	19,737,912	13.8–84.9	3.9	4.7	2.5
	AX-170586625	16	626,899	1.39E-08	1.8	C/T (−3.1)							

^a^ *Chr* abbreviation for Chromosome

^b^ For GWAS panel, the significance value indicated is the p-value, which is significant if lower than the False Discovery Rate

^c^ For GWAS panel, R^2^ is the percentage explained variance corrected for genome-wide background

^d^ For GWAS panel, the allelic effect is the difference in mean of measured trait between genotypes with one or other allele

The sign is with respect to the allele that is first mentioned

^e^ For F_1_ progeny using QTLs detection, the indicated SNP is the peak SNP inside the QTL interval

^f^ LG, abbreviation for Linkage Group with F and U for ‘Franquette’ and ‘UK 6–2’ parental maps respectively

^g^ For F_1_ progeny using QTLs detection, the position in cM indicated in brackets is the 99.9% confidence interval of the QTL

^h^ For F_1_ progeny using QTLs detection, the significance value indicated is the LOD score (LOD = logarithm of the odds ratio)

^i^ For F_1_ progeny using QTLs detection, P.E.V. is for percentage explained variance

^j^ For F_1_ progeny using QTLs detection, d is the estimated substitution effect of the QTL

The high power of our gene tagging approach based on both GWAS and QTL mapping, was also confirmed for beginning, peak, and end, of female flowering dates. The SNP ‘AX-170990138’ (physical position: 9,298,520 bp) on Chr1 was systematically found associated with all stages of female flowering (Table 3). For beginning female flowering date, we found this SNP associated using two-year data, and using each year separately. We also observed this marker-trait association for peak female flowering date using legacy data, and for end female flowering date with two-year data and with 2019 data. The SNP ‘AX-170990138’ is 2.8 Mbp apart from the most significant marker-trait association found for the budbreak date on Chr1, and the allele G of this SNP is linked to a delayed female flowering, with a R^2^ ranging from 34.8 to 39.6%, and an allelic estimated effect ranging from 3.4 to 4.5, depending on the stage and the dataset. We identified additional QTLs for all three stages of female flowering but the most significant ones, segregating in both parental maps, co-localize with those previously found associated with the budbreak date on Chr1 (Table 3).

Besides the major QTL on Chr1 identified with both GWAS and QTL mapping, we found three significant associations also on Chr7 for all three stages. These three SNPs are located in a region of about 23 to 25 Mb (Table 3). In addition, we found QTLs on LGs 2 and 14 in ‘Franquette’ map, and on LGs 3, 9, 11 and 12 in ‘UK 6–2’ map.

### Association and linkage mapping for Heterodichogamy and male flowering dates

Results for male flowering are similar to the female flowering results in that a few SNPs in a very close region are associated with all three stages (Table 4). On Chr11, we found four associated SNPs depending on the stage and the dataset, in a region of about 31.8 Mbp and a window of 52 kb. The most significant QTLs for all three stages of male flowering for both parental maps co-localize with those previously identified as associated with budbreak date and the three stages of female flowering. Two additional QTLs on ‘UK 6–2’ map were found on LG 11 for peak male flowering date, using two-year data (26,141,527 – 35,537,934 bp), and for the end of male flowering using 2019 data (27,685,560 – 35,537,934 bp), supporting the GWAS results.

**Table 4:** Summary of association and linkage mapping results related to heterodichogamy and male flowering dates

Dataset	GWAS using FarmCPU model						QTLs detection using MIM method						
	SNP	Chr ^**a**^	Physical position	Significance ^**b**^	R^**2 c**^	Alleles/Effect ^**d**^	Peak SNP ^**e**^	LG ^**f**^	Physical position	Confidence interval ^**g**^	LOD ^**h**^	P.E.V. ^**i**^	d ^**j**^
**Heterodichogamy**													
1989–2016	AX-171595404	11	31,887,506	2.22E-42	69.6	C/G (2.7)							
2018–2019	AX-171218000	11	31,885,611	8.97E-49	77.2	A/G (− 2.7)							
**Beginning male flowering date**													
2018–2019	AX-171121884	2	21,247,915	1.74E-09	7.2	C/T (−2.5)	AX-171521263	F1	201,441	4.1–9.1	14.4	32.6	4.5
	AX-171578684	4	5,966,307	2.41E-10	6.0	C/T (4.5)	AX-170771092	U1	5,814,444	< 0.5	27.6	52.4	−5.3
	AX-170614801	4	3,642,466	2.78E-10	20.2	C/T (3.6)	AX-170983192	U9	22,032,363	23.6–57.3	5.5	4.9	2.2
	AX-171490851	7	37,022,231	3.56E-10	17.8	C/G (−2.3)							
	AX-170625459	10	10,656,433	2.73E-09	20.2	G/T (−2.4)							
	AX-171147588	11	31,874,617	1.58E-10	2.0	A/G (3.1)							
	AX-170794443	14	10,691,227	2.22E-09	< 0.1	C/T (−2.2)							
	AX-170692084	16	3,578,209	1.94E-08	18.2	G/T (−2.5)							
2018	AX-171589348	3	27,241,886	1.24E-10	< 0.1	C/G (−2.9)	AX-171521263	F1	201,441	2.1–10.4	7.6	36.0	3.8
	AX-171559197	6	13,733,426	9.96E-17	20.7	C/T (3.6)	AX-170771092	U1	5,814,444	0.0–2.0	17.5	50.9	−4.6
	AX-170937789	7	30,051,478	2.70E-12	18.0	C/T (−3.7)							
	AX-170625459	10	10,656,433	4.08E-09	20.6	G/T (−2.2)							
2019	AX-171147582	11	31,882,831	6.23E-13	1.7	C/T (6.2)	AX-171521263	F1	201,441	0.7–13.4	6.4	28.1	5.5
	AX-171525706	14	28,381,698	1.38E-10	10.1	C/G (4.9)	AX-170771092	U1	5,814,444	0.0–5.7	9.2	33.5	−5.9
	AX-170692024	16	3,630,320	1.27E-08	22.8	C/T (−4.2)							
**Peak male flowering date**													
2018–2019	AX-171578684	4	5,966,307	2.17E-09	6.1	C/T (6.8)	AX-171180038	F1	177,178	3.4–10.2	17.1	32.2	5.4
	AX-171147588	11	31,874,617	1.34E-10	1.9	A/G (4.1)	AX-170771092	U1	5,814,444	< 0.5	30.2	47.0	−6.2
							AX-170869355	F2	35,198,358	14.5–67.9	4.6	6.6	−2.3
							AX-170615140	U4	3,291,736	0.0–24.4	6.2	5.0	2.4
							AX-171478663	U5	7,984,929	30.6–64.9	9.2	9.7	3.0
							AX-171548959	U6	12,747,130	0.0–80.5	6.1	5.5	−2.4
							AX-171214747	U9	18,177,182	23.7–57.3	5.1	5.1	2.2
							AX-171030926	U11	33,195,441	2.3–49.4	4.8	5.2	1.8
							AX-170818106	F14	9,871,222	0.0–58.2	5.6	8.3	2.8
2018	AX-170672894	1	1,835,862	4.05E-11	20.6	G/T (3.1)	AX-171180038	F1	177,178	0.0–12.9	7.2	30.1	4.2
	AX-170802286	4	414,952	8.03E-10	< 0.1	A/G (−3.0)	AX-170771092	U1	5,814,444	0.0–5.1	15.8	49.8	−5.5
	AX-171578684	4	5,966,307	1.04E-08	5.5	C/T (4.3)							
	AX-171559197	6	13,733,426	3.12E-12	20.3	C/T (3.2)							
	AX-171571158	12	1,647,435	5.48E-09	< 0.1	A/C (−2.4)							
2019	AX-171578684	4	5,966,307	5.63E-09	8.0	C/T (9.1)	AX-171180038	F1	177,178	0.0–15.6	6.7	28.3	6.1
	AX-171490851	7	37,022,231	1.13E-10	19.2	C/G (−4.3)	AX-170771092	U1	5,814,444	< 0.5	11.8	40.1	−7.6
	AX-171147582	11	31,882,831	1.47E-10	1.5	C/T (5.1)							
	AX-170849618	12	14,608,835	1.85E-08	< 0.1	C/T (−2.9)							
	AX-170692024	16	3,630,320	6.46E-15	22.6	C/T (−6.2)							
**End male flowering date**													
2018–2019	AX-171578684	4	5,966,307	1.01E-09	5.4	C/T (7.9)	AX-171521263	F1	201,441	4.8–8.1	19.0	33.6	6.5
	AX-171560933	5	604,688	1.30E-08	19.2	C/G (−2.8)	AX-170771092	U1	5,814,444	< 0.5	29.8	43.2	−7.1
	AX-171559197	6	13,733,426	6.50E-10	18.2	C/T (3.3)	AX-171539906	F2	34,841,469	29.2–67.9	5.3	6.3	−2.9
							AX-171002599	U5	20,087,966	43.9–78.7	6.3	3.4	2.6
							AX-170677995	U6	1,908,834	0.0–24.0	8.8	7.8	−3.3
							AX-170901663	U12	19,737,912	12.8–85.3	5.6	3.9	2.4
							AX-170818106	F14	9,871,222	0.0–60.6	6.2	9.2	3.2
2018	AX-171559197	6	13,733,426	5.14E-13	20.0	C/T (4.1)	AX-171180038	F1	177,178	1.5–11.1	7.4	30.7	5.4
	AX-171142468	11	31,834,919	9.09E-11	3.4	A/G (5.3)	AX-170771092	U1	5,814,444	0.0–2.2	13.4	44.3	−6.5
	AX-171531310	11	29,400,625	1.53E-08	0.7	C/T (3.3)							
2019	AX-170817347	1	32,640,957	1.07E-09	< 0.1	C/T (4.1)	AX-171180038	F1	177,178	1.2–11.6	9.8	36.1	7.7
	AX-171578684	4	5,966,307	2.05E-09	7.7	C/T (9.9)	AX-170771092	U1	5,814,444	0.0–2.1	18.4	42.6	−8.7
	AX-171217997	11	31,886,671	3.10E-09	0.2	A/G (4.8)	AX-171165000	U5	10,051,966	23.5–78.7	4.1	6.1	3.2
	AX-170692024	16	3,630,320	2.07E-08	23.4	C/T (−4.1)	AX-171583903	U6	2,237,730	0.0–81.0	4.7	6.9	−3.4
							AX-170838105	U11	35,888,157	13.9–49.4	3.7	5.3	2.8
							AX-170901663	U12	19,737,912	25.7–74.8	5.5	8.3	3.8

^a^ Chr, abbreviation for Chromosome

^b^ For GWAS panel, the significance value indicated is the p-value, which is significant if lower than the False Discovery Rate

^c^ For GWAS panel, R^2^ is the percentage explained variance corrected for genome-wide background

^d^ For GWAS panel, the allelic effect is the difference in mean of measured trait between genotypes with one or other allele

The sign is with respect to the allele that is first mentioned

^e^ For F_1_ progeny using QTLs detection, the indicated SNP is the peak SNP inside the QTL interval

^f^ LG, abbreviation for Linkage Group with F and U for ‘Franquette’ and ‘UK 6–2’ parental maps respectively

^g^ For F_1_ progeny using QTLs detection, the position in cM indicated in brackets is the 99.9% confidence interval of the QTL

^h^ For F_1_ progeny using QTLs detection, the significance value indicated is the LOD score (LOD = logarithm of the odds ratio)

^i^ For F_1_ progeny using QTLs detection, P.E.V. is for percentage explained variance

^j^ For F_1_ progeny using QTLs detection, d is the estimated substitution effect of the QTL

For heterodichogamy trait (computed by subtracting peak female date from peak male date; Table S5), the significant associations found with GWAS using two-year data and legacy data co-localized with the associations identified for male flowering dates on Chr11 in the region of about 31.8 Mbp.

### Candidate genes for bearing habit and phenology-related traits using the walnut genome

By combining GWAS and QTL results and considering their consistency over phenotypic datasets, we decided to focus on a robust subset of eight loci to find candidate genes for bearing habit and phenology-related traits (Table 5). Using HaploView v4.2 software and the new chromosome-scale reference walnut genome v2.0 [36], several interesting coding sequences were found within the defined Linkage Disequilibrium (LD) blocks for different traits. The SNP ‘AX-171557178’ on Chr2 and associated with budbreak date, falls within a candidate gene encoding for a *putative BPI/LBP family protein At1g04970*. The corresponding LD block of 78 kb also contains a candidate gene coding for a *GrpE-like protein* and one encoding a *65-kDa microtubule-associated protein 1-like*. Only one candidate gene, encoding an *uncharacterized protein LOC108987988*, overlaps with the most significant SNP associated with budbreak date, ‘AX-171179714’ on Chr1.

**Table 5:** List of candidate genes for bearing habit and phenology-related traits

Trait	SNP associated using GWAS	Chromo-some	Physical position ^**a**^	LD block interval ^**a**^ / Definition block method	GeneID	Physical position interval ^**a**^	Functional annotation
Bearing habit	AX-171191765	11	20,831,267	20,831,267 - 20,865,013 (33 kb) / Solid spine of LD	109,017,990	20,831,874 - 20,834,977	uncharacterized protein LOC109017990
Budbreak date	AX-171179714	1	6,514,832	6,511,521 - 6,528,313 (16 kb) / Solid spine of LD	**108,987,988**	**6,512,784 - 6,520,863**	**uncharacterized protein LOC108987988**
Budbreak date	AX-171557178	2	36,586,423	36,535,050 - 36,613,482 (78 kb) / Solid spine of LD	108,983,408	36,536,110 - 36,536,482	GrpE-like protein
					108,984,642	36,549,629 - 36,555,410	65-kDa microtubule-associated protein 1-like
					108,984,651	36,557,793 - 36,562,849	homolog of mammalian lyst-interacting protein 5
					108,983,414	36,564,185 - 36,568,214	indole-3-acetate O-methyltransferase 1-like
					108,984,666	36,576,304 - 36,582,108	uncharacterized protein LOC108984666
					**108,984,671**	**36,582,611 - 36,587,786**	**putative BPI/LBP family protein At1g04970**
					108,984,682	36,601,277 - 36,607,484	transcription initiation factor TFIID subunit-6-like
					108,984,689	36,610,257 - 36,612,478	cell division protein FtsZ homolog 1, chloroplastic-like
Male flowering date	AX-171147588	11	31,874,617	31,868,402 - 31,887,998 (19 kb) / Solid spine of LD	108,984,910	31,868,783 - 31,870,048	uncharacterized protein LOC108984910
					108,984,907	31,884,256 - 31,887,060	probable trehalose-phosphate phosphatase D
Male flowering date	AX-171578684	4	5,966,307	5,946,348 - 6,009,859 (63 kb) / Solid spine of LD	109,020,754	5,953,569 - 5,954,197	uncharacterized protein LOC109020754
					109,022,114	5,954,514 - 5,956,355	trichome birefringence-like 13 protein
					109,022,113	5,960,058 - 5,960,714	uncharacterized protein LOC109022113
					109,022,028	5,984,567 - 5,985,798	uncharacterized protein LOC109022028, transcript variant X2
Male flowering date	AX-171559197	6	13,733,426	13,733,274 - 13,733,426 (< 1 kb) / Confidence interval		–	–
Hetero-dichogamy	AX-171218000	11	31,885,611	31,868,402 - 31,887,998 (19 kb) / Solid spine of LD	108,984,910	31,868,783 - 31,870,048	uncharacterized protein LOC108984910
					**108,984,907**	**31,884,256 - 31,887,060**	**probable trehalose-phosphate phosphatase D**
Female flowering date	AX-170990138	1	9,298,520	9,298,520 - 9,300,288 (1 kb) / Confidence interval	108,998,539	9,298,602 - 9,300,352	chromosome transmission fidelity protein 8 homolog, mRNA

^a^ Physical position given in bp

The candidate genes in bold overlap the physical position of the associated SNP

**Fig. 6:**
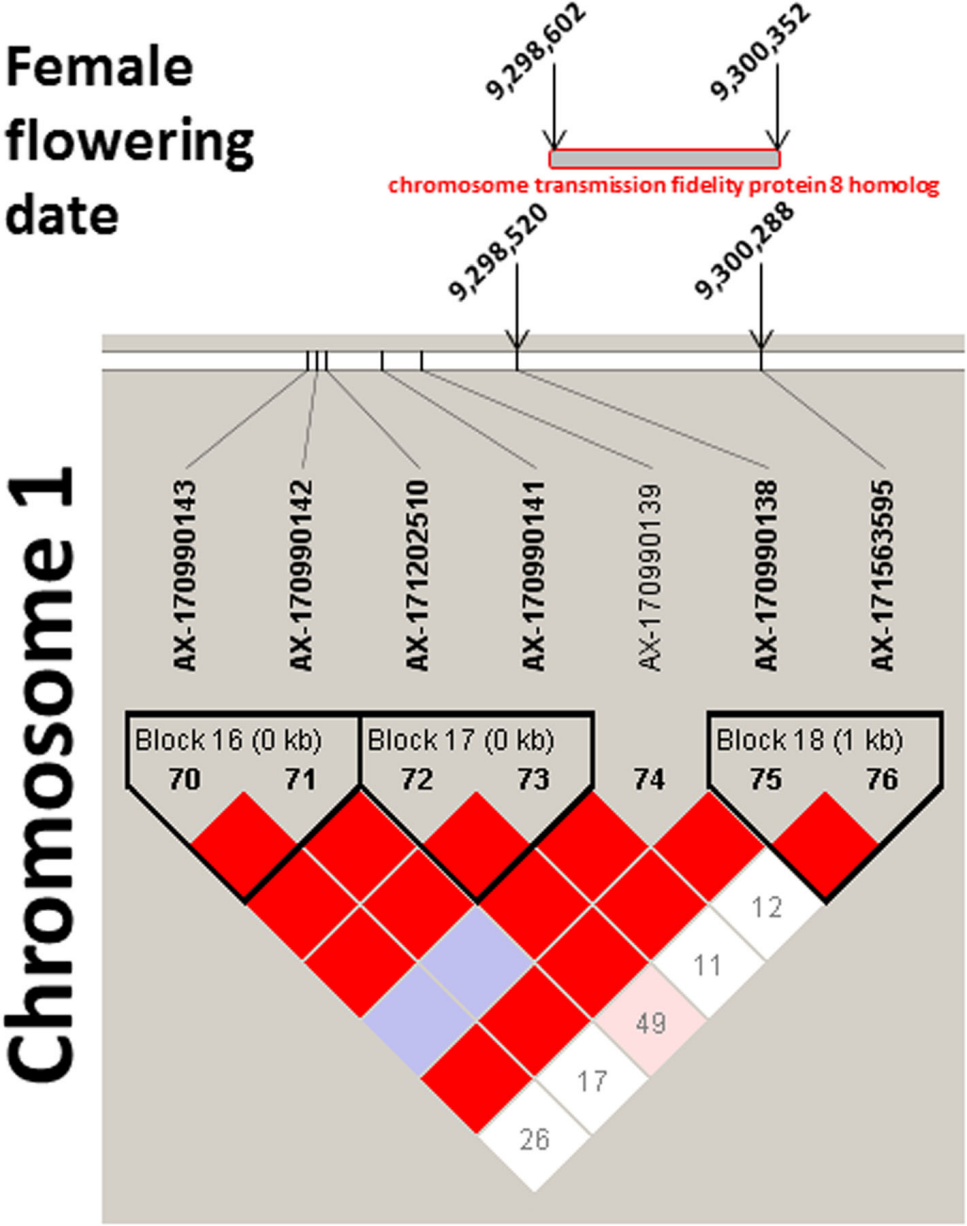
LD block representation for the SNP AX-170990138 associated with female flowering dates. This SNP overlaps with a candidate gene, coding for a *chromosome transmission fidelity protein 8 homolog*

The two SNPs on Chr11 associated with all three stages of male flowering date and with heterodichogamy, belong to the same LD block of 19 kb. Within this block is located a candidate gene encoding for a *probable trehalose-phosphate phosphatase D*. The other SNP on Chr4 associated with all three stages of male flowering date, belongs to a LD block of 63 kb comprising a candidate gene encoding for a *trichome birefringence-like 13 protein*. Only one candidate gene was found in LD with the associated SNP on Chr1 for all three stages of female flowering date. The SNP ‘AX-170990138’ belongs to a small LD block on Chr1 spanning from 9,298,520 to 9,300,288 bp (Fig. 6). The identified candidate gene of 1.75 kb (interval from 9,298,602 to 9,300,352 bp) overlaps with the LD block and encodes a *chromosome transmission fidelity protein 8 homolog*.

### Development of KASP marker for the Budbreak date and validation using 96 accessions of the walnut improvement program of the University of California, Davis

**Fig. 7:**
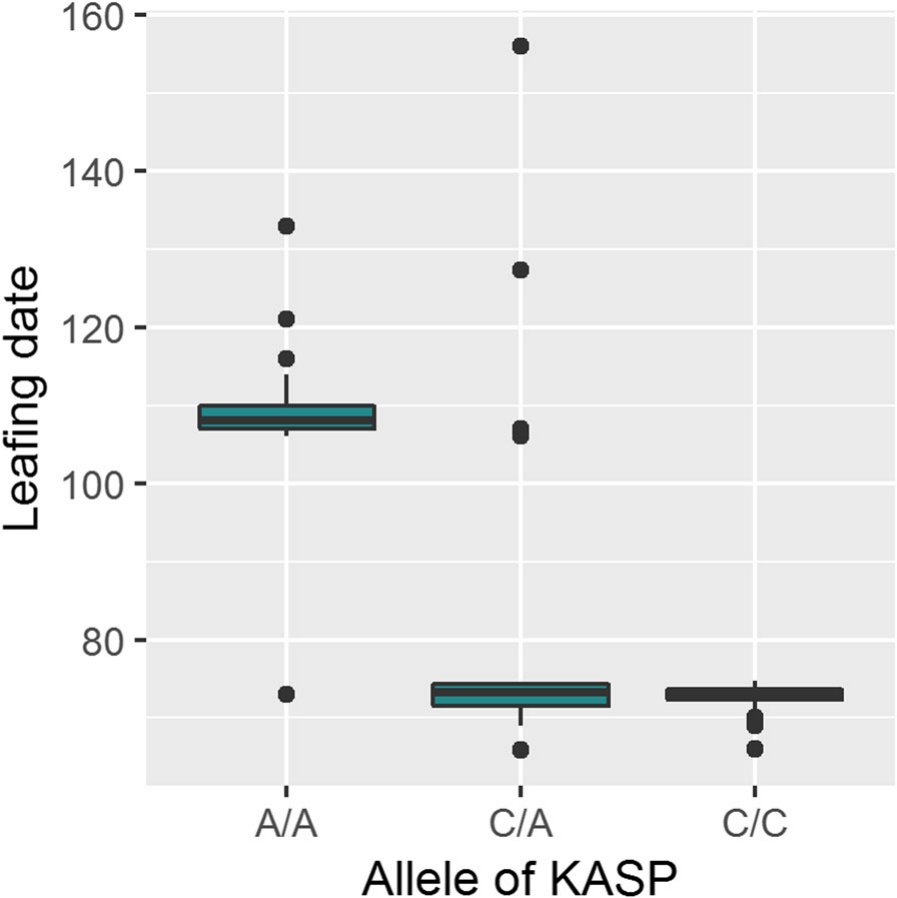
Allelic effect of the KASP marker associated to the leafing date. 96 unreleased breeding line accessions of the Walnut Improvement Program of the University of California, Davis, were used

Primers related to the marker-trait association found on Chr1 for budbreak date (SNP ‘AX-171179714’) were developed. These primers were used to genotype 96 unreleased breeding line accessions of the Walnut Improvement Program of the University of California, Davis. Among them, 48 are early leafing accessions and 48 are late leafing accessions. Leafing date occurs 3 days after budbreak date. Our GWAS results showed that the allele T of the SNP ‘AX-171179714’ (versus G) has an allelic estimated effect of 5.9 (R^2^ = 30.6%), linked with a delayed budbreak date. Since we designed the KASP primers using the complementary strand, we expect to find the allele A (versus C) as involved in late leafing genotypes. The three different genotypes were clearly separated for 95 accessions, whereas one of them could not be determined (Table S4). In order to determine if leafing dates were different among the three genotypes, we ran a Kruskal-Wallis test which showed significance (*p*-value = 6.88E-13). Then, we ran a pairwise comparison using Dunn’s test with Bonferroni p-value adjustment. Results of pairwise comparisons showed that A/A vs. C/A, and A/A vs. C/C have a significant effect on leafing date (*p*-values = 1.4E-06 and 9.4E-12, respectively), contrary to C/A vs. C/C genotypes (p-value = 1). The homozygous accessions A/A have a median leafing date of almost 110 Julian days, while genotypes C/A and C/C have a median leafing date of only 76 Julian days (Fig. 7).

## Discussion

### Analysis of population structure and its effect on bearing habit and phenology-related traits

We found that the most likely K subpopulations were K = 2, with a clustering in two major groups according to their geographical origin, and K = 3 that highlights admixed accessions, including hybrids or modern cultivars from France and USA. The EEAs cluster in PCA resulted more scattered, indicating a higher level of SNP-based genetic diversity, and the three most diverse accessions of the EAAs cluster, located at the bottom-right corner of the PCA plot, are ‘Shinrei’ from Japan, ‘JinLong1’ from China, and ‘EAA6’ from Greece. The two last were previously found to be highly diverse by SSRs and included in an SSR-based core collection [37].

For the entire set of studied traits, we observed no influence of population structure on the phenotypes. Most likely, the familial relatedness among accessions accounted for most of the genetic variation affecting the traits studied, leading to redundant PCA information [38]. Even though our collection included accessions from different geographical areas, it consisted of 30% modern cultivars likely brought to the INRAE of Bordeaux as potential genitors for breeding purposes. Therefore, it is not surprising to observe accessions of the same geographical origin that are both early and late in phenology.

### Strong correlations between traits Lead to close associations

Results found using the F_1_ progeny show that strong QTLs for all the stages of female flowering date segregating in both parents on LG 1, co-localize with strong QTLs for budbreak date. In walnut trees, budbreak phenotyping relies on buds that will produce both leaves and female flowers. Those traits are, therefore, correlated and likely controlled by common genetic and metabolic pathways, as suggested by the identification of QTLs within the same genomic region on Chr1. Similar results were observed in walnut for leafing and harvest dates [35]. On the other hand, GWAS enabled further dissection of the genetic control of these traits by identifying a location for the most significant marker-trait associations for budbreak date at about 2.8 Mbp apart from the SNPs associated to all stages of the female flowering date. This is one of the major advantages of GWAS, whose higher resolution defines more precisely the underlying regions of traits of interest compared to classical QTL mapping.

While the major loci controlling the traits studied were identified with both GWAS and linkage mapping, this did not happen for the minor associations. For instance, for budbreak date, GWAS found a second trait-locus association on Chr2, while one minor QTL was detected on LG 6 of the ‘UK 6–2’ map. This finding can be explained in two ways: (*i*) the loci identified in the GWAS likely are not segregating in the F_1_ progeny, and (*ii*) the QTLs found in the linkage mapping analysis have rare alleles in the association panel. For both GWAS results and QTLs mapping, the minor trait-loci associations were not stable across years, suggesting they are more affected by environmental conditions. For this reason, we decided to focus mostly on the GWAS results based on the two-year BLUPs. Nevertheless, results based on the legacy data allowed confirmation of the major SNP associated with budbreak date.

We observed a different scenario for all the stages of male flowering date: while the major QTL found on LG 1 co-localizes with those for female flowering dates and budbreak date, these results were not confirmed in the GWAS panel. Here, instead, we identified the most significant associations on chromosomes 11 and 4. Then, since heterodichogamy is the measurement of how the male and female flowering dates overlap, it is not surprising to find a marker-trait association on Chr11 with more consistency across years. Since the F_1_ progeny used for QTL mapping included only 78 individuals, likely leading to an overestimation of the QTL effects (Beavis effect) [39], we decided to analyze the GWAS results in more depth. In addition, a few regions were not segregating in our F_1_ progeny, due to monomorphism in the parents, likely preventing accurate QTL detection. This is the case, for example, of the QTLs detected on LG 2 for both parental maps. By checking the physical position of the associated markers, we observed that the beginning of the Chr2 lacks regions from 0 to 5 Mbp for the ‘Franquette’ map, and from 0 to 7.7 Mbp for the ‘UK 6–2’ map.

Combining multi-locus models for GWAS has become widespread [40], and the associations found by multiple methods are usually reliable [41]. Similar work on walnut, also using MLMM and FarmCPU models [35], found three associations within a physical genomic region spanning from 3,187,214 to 4,805,396 bp for the leafing date. This is very close to our association for budbreak date, detected at 6,514,832 bp, and our QTL for the same trait using the ‘Franquette’ parental map, located at 87,347 to 3,051,320 bp. These results indicate that most likely on Chr1 there is a genomic region controlling budbreak, leafing, and female flowering dates in walnut. Since strong positive correlations exist among these traits, with high allelic effects of the marker-trait associations, this would have an impact for selection. For instance, breeding for budbreak and female flowering (and more broadly for all phenological-related traits) would be a difficult job, and would complicate the creation of shorter-cycle cultivars.

In this work, classical QTL mapping mostly confirmed the major marker-trait associations identified. We found a major locus on Chr1 for budbreak date and female flowering dates, and a major locus on Chr11 for male flowering dates. Separating the flowering stages (beginning, peak and end) allowed “repeat” phenotyping, generating robust data for identification of the major loci required for marker development purposes. Also, the high complexity of phenology-related traits indicates that many loci may be involved in their expression [23], making the detection of minor loci difficult.

### Genetic bases for the expression of lateral bearing

Lateral bearing is a crucial trait in walnut breeding since it contributes to increased yield [42]. Fruiting at lateral positions on shoots was originally introduced into California from the cultivar ‘Payne’, which is in the background of all current varieties released by the Walnut Improvement Program of the University of California, Davis [4]. Using a large F_2_ progeny from ‘Chandler’, a lateral bearing cultivar heterozygous for this locus, the genetic basis of lateral fruitfulness was found to be located in the centromeric region of Chr11 [29]. The same location was obtained using F_1_ progeny from ‘Chandler’ by ‘Idaho’ cross [43]. Additional associations for lateral bearing were found using GWAS in a region covering 25 Mb on Chr11, and a major QTL was detected in ‘Chandler’ in proximity to the centromeric region (peak at 17,341,634 bp) of Chr11 [35].

In the present study, we reconfirmed that walnut bearing habit is controlled by a major QTL in the centromeric region of Chr11. In line with previous studies, we observed that an allelic substitution at the most associated locus, ‘AX-171191765’, causes lateral bud fruiting. We confirm previous results regarding genetic control of lateral bearing by performing GWAS in genetically different plant materials, further supporting the power and high resolution of this method in walnut. In our F_1_ progeny, this trait did not segregate and all hybrids are lateral bearing. ‘Franquette’ is terminal bearing and homozygous G/G for this locus, whereas ‘UK 6–2’ is lateral bearing and heterozygous G/C for this locus. Maybe because our progeny is too small or because of segregation distorsion, we did not observe any terminal bearing individuals.

### Gene annotations may give clues to dissect genetic control of flowering process

Availability of the new chromosome-scale reference genome Chandler v2.0 [36] allowed us to explore the gene space surrounding the most significant marker-trait associations identified for bearing habit and phenology. For instance, we found the *chromosome transmission fidelity (ctf) protein 8 homolog* gene as candidate gene for all female flowering dates. Previous studies in *Arabidopsis thaliana* suggest the involvement of the *ctf* genes family in the cell division processes [44]. In particular, the inactivation of the *ctf7–1* and *ctf7–2* genes resulted in developmental defects, including aberrations in flower morphology and male and female gametophytes. Interestingly, a TPX2 encoding gene required for spindle assembly during cell division process was also found likely to be involved in harvest date determination in walnut [35]. These findings suggest that cell division is a crucial process for correct flower and fruit development in walnut, and suggest the *ctf8 protein homolog* locus is an excellent candidate gene for female flowering.

Trichomes consist of only one cell type, appearing as a long, slender appendage [45]. In *J. regia*, during staminate flower development, the tepals differentiate into anthers and filaments shortly before anthesis, enveloping the stamens [46]. Since these tepals become hirsute, it is not surprising to find a *trichome birefringence-like 13 protein* encoding gene within the LD block surrounding the SNP on Chr4 which is associated with male flowering dates. Also, the SNP on Chr11 associated with heterodichogamy and male flowering dates is in LD with a candidate gene encoding a *probable trehalose-phosphate phosphatase D*. This dephosphorylating enzyme and other members of the gene family are highly expressed in male flowers of *Jatropha*, a perennial woody plant, and in transgenic plants of *A. thaliana* with delayed flowering [47], suggesting the putative involvement of this class of genes in the flower development process. Other authors reviewed the roles of sugars in the control of flowering time, and apart from its involvement in carbohydrate metabolism, trehalose-6-phosphate seems to serve as a signal for inducing flowering transition in plants [48, 49]. In this regard, we found a *probable trehalose-phosphate phosphatase D* encoding gene for heterodichogamy.

Surprisingly, we did not find widely known genes to be involved in flowering process or dormancy in our candidate genes. In *A. thaliana*, sweet cherry or other species, these mechanisms are regulated by *flowering locus*, *early flowering*, and *MADS-box* homologous genes, among others [50–52]. For this reason, we decided to list several homologous genes within the RefSeq walnut gene annotation using three keywords: ‘flower’, ‘dormancy’, ‘MADS’, and we checked the physical positions of these genes. We then compared them with our marker-trait associations, regardless of the LD blocks, and we found interesting results. We found a *agamous-like MADS-box protein AGL11* encoding gene (approx. 2.0 Mbp) and a *MADS-box transcription factor 6-like* encoding gene (8.8 Mbp), both at the beginning of the Chr1. The first one co-localizes with the major QTL found for budbreak date using the F_1_ progeny and the second is very near to the major marker-trait association found for female flowering dates (9.3 Mbp). Then, our results showed several marker-trait associations for both female and male flowering dates on Chr7 between 23.0 and 48.0 Mbp. Interestingly, we found the following encoding genes within this window: a *flowering time control protein FCA-like* (33 Mbp), a *flowering-promoting factor 1-like protein 3* (34.4 Mbp), a *dormancy-associated protein homolog 4* (42.9 Mbp), a *protein early flowering 3-like* (44.3 Mbp), a *MADS-box protein SOC1-like* (48.0 Mbp), and a *protein flowering locus D-like* (48.6 Mbp). Finally, we noticed that two genes are near to our marker-trait associations for the dichogamy (31.9 Mbp) and the male flowering dates (from 29.4 to 31.9 Mbp) on Chr11: a *MADS-box transcription factor 27-like* (29.9 Mbp) and a *protein embryonic flower 1* (34.3 Mbp).

### A KASP marker related to phenology is released for the first time in walnut

For the first time in walnut, a KASP marker related to phenology is released. By designing specific primers for the SNP AX-171179714, we found that the homozygous accessions A/A are significantly later leafing. Since this marker is dominant, it will greatly help breeders to accurately select individuals with delayed budbreak. However, due to the complex genetic basis of phenology-related traits in perennials [23], additional markers, especially for minor loci, will be needed to improve the selection. Finally, this SNP is located in a gene encoding an uncharacterized protein, and it would be interesting to know more about the functional role of this gene. The marker can predict a significant portion of the phenotype but we still do not know the regulatory networks involved in this complex trait.

## Conclusions

Due to the significant influence of environment on phenology-related traits in walnut, unravelling their genetic architecture is of upmost importance for the development of markers that could assist the selection of superior genotypes and, therefore, the release of new walnut cultivars adapted to different climatic conditions. Using GWAS with two different multi-locus models, we identified significant associations for budbreak date, and male and female flowering dates, confirmed by classical QTL mapping. In addition, we provide a list of candidate genes for these traits, that will be fundamental in future studies of functional genomics and understanding the metabolic pathways underlying phenology in walnut. We also developed and validated the first KASP marker for budbreak date in walnut, which will allow accurate selection of individuals with a delayed budbreak and, therefore, suitable for cultivation in France and other regions where late spring frosts are challenging. In parallel, the genetic bases for the expression of lateral bearing were confirmed. Since the future French walnut breeding program needs cultivars with high quality kernels, efforts are underway phenotype the entire collection regarding chemical content (e.g. fatty acids and tocopherols) and physical characteristics (nut length, filling ratio, and ease of removal). Our F_1_ progeny is too small to pursue investigations but this study confirms that the INRAE walnut germplasm repository contains an array of plant material suitable for this type of work. New genome-wide analyses now are being initiated to further increase our knowledge concerning the genetic architecture of the main traits of interest in walnut.

## Methods

### Plant material

The panel for GWAS consists of 170 unique *J. regia* grafted trees accessions originating worldwide and maintained at the *Prunus* and *Juglans* Genetic Resources Center located in the Fruit Experimental Unit of the INRAE, Toulenne, France (44°34′37.442″N – 0°16′51.48″W), near Bordeaux. This INRAE walnut germplasm collection is publicly available and is a result of important collecting work performed between 1988 and 2000 by Eric Germain (retired and former head of the INRAE walnut breeding program) in 23 countries including the European, American, and Asian continents. Eric Germain initiated an international cooperation during his activity concerning the exchange of walnut plant materials (especially in Europe) and obtained all permissions to bring them. Experimental research on this plant material complies with our INRAE institutional guidelines. The original source of each accession (institute/lab source or collecting source) is given (Table S1). The panel choice was based on previous genetic diversity work using 13 SSR markers and evaluation of phenotypic variability [37].

The intraspecific F_1_ mapping progeny results from bi-parental controlled crosses made from 1997 to 2004 by Eric Germain between two cultivars with contrasting phenology-related traits, for his needs under his breeding program. The female parent was the ‘Franquette’ cultivar, a French landrace from Isère valley and having a late budbreak date and terminal bearing. The male parent was the accession ‘UK 6–2’, obtained from the Kiev Botanical Garden in Ukraine and thought to originate from the center of origin of *J. regia* in Central Asia (Uzbekistan, Tajikistan, or Kyrgyzstan). ‘UK 6–2’ has an early to intermediate budbreak date and lateral bearing. The progeny consists of 78 individuals, also located at the Fruit Experimental Unit of the INRAE in Toulenne, which were previously genotyped using the same SSR markers as those of the GWAS panel [37] to confirm that they were true progeny. This plant material also complies with our INRAE institutional guidelines.

### Phenotypic evaluation and data analysis

Phenotypic evaluation for the following 10 traits was conducted in 2 years (2018 and 2019) for both the GWAS panel and the F_1_ progeny, at a rate of two to three visits in orchards per week during the months of March, April and May: budbreak date, beginning, peak, end, and duration of female flowering, heterodichogamy, and beginning, peak, end, and duration of male flowering (Table S5). Heterodichogamy, degree of male and female flowering overlap, was computed by subtracting peak female flowering date from peak male flowering date. A negative value (categories ‘1’ and ‘3’, Table S5) means the accession is protandrous since peak male flowering in Julian days occurs before the peak female date. Categories ‘7’ and ‘9’ indicate protogyny.

For the GWAS panel, we also used phenotypical data based on the same scoring scales used for many years previously (mainly between 1989 and 2011) on three sites. These legacy data are available from the INRAE walnut germplasm collection database [21]. Moreover, as bearing habit is not affected by environment conditions, this trait was recorded only in 2019. This trait did not segregate within the F_1_ progeny, so only the GWAS panel was evaluated. Lateral bearing was found in recently released cultivars from France (‘Ferbel’, ‘Fertignac’) and the USA (‘Pedro’, ‘Chico’, ‘Cisco’, ‘Chandler’), and in accessions coming from the Central Botanical Garden of Kiev, Ukraine (Table S1), supposedly originating from the walnut center of domestication, according to the introduction book of the INRAE. Terminal bearing was observed mainly in French landraces such as ‘Franquette’, ‘Grosvert’ and ‘Verdelet’. Data management and visualisation, and Spearman correlation matrices (more appropriate for discrete variables) were performed using “R” software [53], with the packages “tidyverse” [54] and “corrplot” [55], respectively.

For both the GWAS panel and the F_1_ progeny, the means of genotypic effects were obtained for each accession by adjusting for known environmental factors using the BLUPs. When using two-year data, the means were predicted using a mixed linear model considering the year effect (a). When using phenological data from many years and three sites only available for the GWAS panel [21], the means were predicted using a mixed linear model considering the effects of year, site and combined of year and site (b):

a $$ {P}_{ik}=\mu +{Y}_i+{g}_k+{e}_{ik} $$

b $$ {P}_{ijk}=\mu +{Y}_i+{S}_j+\left({Y}_i\times {S}_j\right)+{g}_k+{e}_{ijk} $$

where *P*_*i(j)k*_ refers to the observed phenotype of the *k*^th^ accession in the *i*^th^ year in the *j*^th^ site; *μ* is the mean value of the trait; *Y*_*i*_ is the fixed effect of the *i*^th^ year, *S*_*j*_ is the fixed effect of the *j*^th^ site, *g*_k_ is the random effect of the *k* genotype; and *e*_*i(j)k*_ is the residuals of the model. The BLUPs were performed using “R” package “lme4” [56].

Based on the previous mixed linear models, broad-sense heritability of each trait was estimated using the variance components. When using two-year data, we accounted for the variance component of genotype × year interaction (a). When using phenological data from many years and three sites, we accounted for the variance components of genotype × year and genotype × site interactions (b):

a $$ {\mathrm{H}}^2={\sigma^2}_G/\left[\right({\sigma^2}_G+\left({\sigma^2}_{GxY}/{\mathrm{n}}_{\mathrm{y}}\right)+\left({\sigma^2}_{\varepsilon }/{\mathrm{n}}_{\mathrm{y}}\right)\Big] $$

b $$ {\mathrm{H}}^2={\sigma^2}_G/\left[\right({\sigma^2}_G+\left({\sigma^2}_{GxY}/{\mathrm{n}}_{\mathrm{y}}\right)+\left({\sigma^2}_{GxS}/{\mathrm{n}}_{\mathrm{s}}\right)+\left({\sigma^2}_{\varepsilon }/{\mathrm{n}}_{\mathrm{y}}{\mathrm{n}}_{\mathrm{s}}\right)\Big] $$

where σ^2^_*G*_ is the genotypic effect variance; σ^2^_*GxY*_ is the genotype × year interaction variance; σ^2^_*GxS*_ is the genotype × site interaction variance; σ^2^_*ε*_ is the variance of residuals; n_y_ is the number of years; and n_s_ is the number of sites. We have always only one replication by genotype. For two-year data, n_y_ = 2 (2018 and 2019). Nevertheless, the legacy data are very unbalanced for the years and sites available considering each genotype. We consider n_y_n_s_ = 5 because, in average, we have 5 observations by genotype.

### SNP genotyping and quality control

The genomic DNAs of both the GWAS panel (*J. regia* accessions) and F_1_ progeny (78 individuals) were extracted from young leaves as described in Bernard et al. [37]. The accessions were genotyped using the Axiom™ *J. regia* 700 K SNP array containing 609,658 SNPs uniformly distributed over the 16 *J. regia* chromosomes [31]. These SNPs were then filtered through several criteria (Table 2). First, the filtering metrics were performed by ThermoFisher considering default thresholds: dish quality control greater than or equal to 0.82, and quality control call rate of 97%. The quality control steps were performed using “PLINK 1.9” software [57]. SNPs with Mendelian errors in the F_1_ progeny were removed. After, Poly High Resolution (PHR) and No Minor Homozygotes (NMH) SNPs were filtered using stringent thresholds: SNP call rate (> 90%), minor allele frequency (MAF > 5%), and redundancy in the genome (SNP probes aligning in duplicated regions). Finally, 364,275 robust SNPs (59.8% of the total number of SNPs) were retained for the following genome-wide analysis.

The same steps were performed for the F_1_ progeny, except filtering for MAF, gaining a final panel of 478,458 SNPs. In addition, individuals were checked for outlying heterozygosity rate.

### SNP linkage map construction and quantitative trait locus analyses

Based on the pseudo-testcross strategy [58], 849 SNPs were retained for the ‘Franquette’ female parent, and 1088 for the ‘UK 6–2’ male parent. The two parental genetic linkage maps were constructed using JoinMap® 4.0 software [59] and distorted markers were omitted. A minimum LOD value of 16.0 was chosen for mapping. Kosambi’s mapping function was used to convert recombination frequencies into map distances [60], and graphical display of linkage maps was performed using MapChart 2.32 software [61].

QTLs were determined using MultiQTL 2.6 software (http://www.multiqtl.com/; MultiQTL Ltd., Institute of Evolution, Haifa University, Haifa, Israel). Single trait analysis was performed using a Multiple Interval Mapping (MIM) method [62], in which the highest effect QTL is taken as a cofactor to control the genetic background, whereas another QTL is searched in a different position. All LGs for each parent were scanned using the one QTL model, with 1000 runs of permutation tests, to compare H1 hypothesis (one QTL is present in the LG) and H0 hypothesis (no QTL in the LG). The threshold for MIM was 0.05 and computation of Type I error rate for each QTL (α_chr_) was performed as follows:

$$ {\alpha}_{\mathrm{chr}}=1-\left\{1-\right[1-{\left(1-0.05\right)}^{1/M}\Big\}{}^m $$

where *M* is the total number of markers used for the QTL detection on each parental map, and *m* is the number of markers in the LG carrying the QTL [63]*.*

### Population structure and kinship analyses

For the GWAS panel, the R packages “SNPRelate” and “gdsfmt” [64] were used to perform PCA and relatedness estimations based on a LD pruned set of 29,825 SNPs to avoid capturing too much variance of high LD regions. For the PCA, the ten largest eigenvalues were obtained to check for structure. The KING method of moment was used to estimate identity-by-descent (IBD) proportions between all pairwise comparisons [65]. Population structure was also investigated using the “fastSTRUCTURE” software [66], and the most likely K was determined using the ΔK method [67]. Then, “CLUMPP” [68] and “distruct” [69] softwares were used for clustering accessions and graphical presentation respectively.

### Genome-wide association analysis

GWAS was carried out using the R package “GAPIT” [70], accounting for the familial relationships in the form of a kinship matrix. The ‘model selection’ function implemented in GAPIT was used to select the best number of PCs by running a mixed linear model (MLM) with a maximum of ten PCs tested. Then, the best number of PCs to include in the GWAS analysis was selected according to the Bayesian Information Criterion (BIC). Using BLUPs previously calculated as phenotypic data and each year separately, two multi-locus models were applied: multi-locus mixed model (MLMM) [71] and Fixed and random model Circulating Probability Unification method (FarmCPU) [72]. Thresholding with FDR [73] classically implemented in “GAPIT” was applied to define the significant associations.

### Search of annotations within LD blocks of associated loci

LD levels around the most associated loci were estimated using HaploView v4.2 software [74]. Each physical position of these trait-SNPs associations was investigated to explore the extension of the surrounding LD blocks and determine the genomic regions where to search for candidate genes. The LD blocks were investigated using the method of confidence interval [75], and “solid spine of LD” method, in which the first and last SNPs in a block are in strong LD with all intermediate markers but the intermediate ones are not necessarily in LD with each other. The identified LD blocks were then searched for candidate genes using RefSeq walnut gene annotation (https://www.ncbi.nlm.nih.gov/genome/annotation_euk/Juglans_regia/100/) and mapped onto the new chromosome-scale reference genome v2.0 [36] (https://www.hardwoodgenomics.org/Genomeassembly/2539069).

### Kompetitive allele specific PCR marker development for Budbreak date and validation

Kompetitive allele specific PCR (KASP) markers are based on the dual Fluorescence Resonance Energy Transfer (FRET) method, in which the sample DNA is amplified with allele specific primers conjugated to fluorometric dyes HEX and FAM at their 5′ end. Based on GWAS results regarding the budbreak date, the following KASP primers were developed (LGC Genomics, Hoddesdon, UK) to target the most significantly associated SNP with this trait:

Allele A: 5′-AGGACAGCAATAAACTCAATCACACA-3′.

Allele C: 5′-GGACAGCAATAAACTCAATCACACC-3′.

Allele A tail (FAM tail): 5′-GAAGGTGACCAAGTTCATGCT-3′.

Allele C tail (HEX tail): 5′-GAAGGTCGGAGTCAACGGATT-3′.

Common reverse: 5′-AGGTTCTGCCAAGCTACAGGGTATA-3′.

These primers were developed by the BioGEVES laboratory (Beaucouzé, France) on the complementary strand. The KASP reaction and its components are explained at https://www.biosearchtech.com/support/education/kasp-genotyping-reagents/how-does-kasp-work. The KASP reaction was prepared as follows: 1.95 μL of PCR mix PACE (3CR Biosciences Limited, Harlow, UK), 2 μL of genomic DNA (2 ng/μL), and 0.053 μL of the three primers (Integrated DNA Technologies, Leuven, Belgium), for a total volume reaction of 4.003 μL. Amplification was performed in a hydrocycler, starting with 15 min at 94 °C, a touchdown phase of 10 cycles at 94 °C for 20 s and at 61 °C for 60 s with a 0.6 °C decrease in temperature per cycle, followed by 35 cycles of 94 °C for 20 s and 55 °C for 60 s. Once the cycle was complete, Fluostar Omega reader (BMG Labtech, Paris, France) was used to read the fluorescence signal. The KASP method was tested using DNA from a set of 96 unreleased breeding line accessions from the Walnut Improvement Program of the University of California, Davis.

## Supplementary information

**Additional file 1: Table S1.** List of association panel accessions with their origin, breeding level, pedigree when known, and their membership to clusters based on fastSTRUCTURE results for K = 3.

**Additional file 2: Table S2.** Number of principal components required for bearing habit trait using Bayesian Information Criterion.

**Additional file 3: Table S3.** ‘Franquette’ and ‘UK 6–2’ parental maps for QTL detection.

**Additional file 4: Table S4.** Genotyping results of SNP AX-171179714 by KASP.

**Additional file 5: Table S5.** Walnut trait ontology.

**Additional file 6: Table S6.** Phenotypic raw dataset of the GWAS panel.

**Additional file 7: Table S7.** Phenotypic raw dataset of the F_1_ progeny.

**Additional file 8: Figure S1.** Scatter plots showing the two-year data related to phenological traits in Julian days for the 170 accessions of the GWAS panel.

**Additional file 9: Figure S2.** Density plots showing the two-year data related to phenological traits in Julian days for the 170 accessions of the GWAS panel.

**Additional file 10: Figure S3.** Scatter plots showing the two-year data related to phenological traits in Julian days for the 78 accessions of the F_1_ progeny.

**Additional file 11: Figure S4.** Density plots showing the two-year data related to phenological traits in Julian days for the 78 accessions of the F_1_ progeny.

**Additional file 12: Figure S5.** Detection of the number of clusters using a) Prob(K), and b) deltaK method (Evanno et al., 2005) in the GWAS panel.

**Additional file 13: Figure S6.** Principal Component Analysis performed on the GWAS panel. The two first principal components show accessions colored according to fastSTRUCTURE results with EEAs for Eastern Europe and Asia, and WEAm for Western Europe and America.

**Additional file 14: Figure S7.** Scatter plot showing the estimated kinship coefficients by the proportion of zero Identical-By-State (IBS0) in the F_1_ progeny.

**Additional file 15: Figure S8.** Level plot showing the Identical-By-State (IBS) values for the 170 accessions of the GWAS panel.

**Additional file 16: Figure S9.** Genetic maps and QTLs detected using two-year data. F(X) and U(X) are the linkage groups of ‘Franquette’ and ‘UK 6–2’ parental maps respectively. Legend of the QTLs: black for budbreak date, red for beginning female flowering date, deep green for full female flowering date, blue for end female flowering date, yellow for beginning male flowering date, pink for full female flowering date, and light green for end female flowering date. Solid bars indicate the 95% confidence interval of the QTL, and terminal bars indicate the 99.9% confidence interval of the QTL. The marker names were changed with the corresponding chromosome number and its physical position for a better visualization.

## Data Availability

The phenotypic raw datasets generated and analyzed during the current study for the GWAS panel and for the F_1_ progeny are available respectively in Table S6, and Table S7. The SNP genotyping raw datasets in “hapmap” format are freely and openly accessed on the “Portail Data INRA” repository, via the identifier “INRA’s Walnut Hapmap” and the following Digital Object Identifier (DOI): 10.15454/XPKII8. The dataset called “GWAS_hapmap.txt” is related to the GWAS panel, and the one called “Progeny_hapmap.txt” is related to the F_1_ progeny. The additional file called “List of ID.tab” allows to link the array identifier name of the accessions with the identifier name used in this study.

## Abbreviations

ANRT: Agence nationale de la recherche et de la technologie
BIC: Bayesian information criterion
BLAST: Basic local alignment search tool
BLUPs: Best linear unbiased predictions
Chr: Chromosome
cM: centiMorgan
CTIFL: Centre technique interprofessionnel des fruits et légumes
FAO: Food and agriculture organisation
FarmCPU: Fixed And random model circulating probability unification
FDR: False discovery rate
FRET: Fluorescence resonance energy transfer
GEMMA: Genome-wide efficient mixed model association
GEVES: Groupe d’etude et de contrôle des variétés et des semences
GWAS: Genome-wide association study
IBD: Identity by descent
IBS: Identity by state
INRAE: Institut National de Recherche pour l’Agriculture, l’Alimentation et l’Environnement
KASP: Kompetitive Allele Specific PCR
LD: Linkage disequilibrium
LG: Linkage group
LOD: Logarithm of odds
MAF: Minor allele frequency
MIM: Multiple interval mapping
MLM: Mixed linear model
MLMM: Multi-locus mixed model
NCBI: National center for biotechnology information
NMH: No minor homozygote
PCA: Principal component analysis
PHR: Poly high resolution
QTL: Quantitative trait locus
SNP: Single nucleotide polymorphism
SSR: Simple sequence repeat
