# Supplementary figures and images for: Association and linkage mapping to unravel genetic architecture of phenological traits and lateral bearing in Persian walnut (Juglans regia L.)
Source: BMC Genomics. 2020 Mar 4;21:203. doi: 10.1186/s12864-020-6616-y (PMC7057608; doi:10.1186/s12864-020-6616-y)

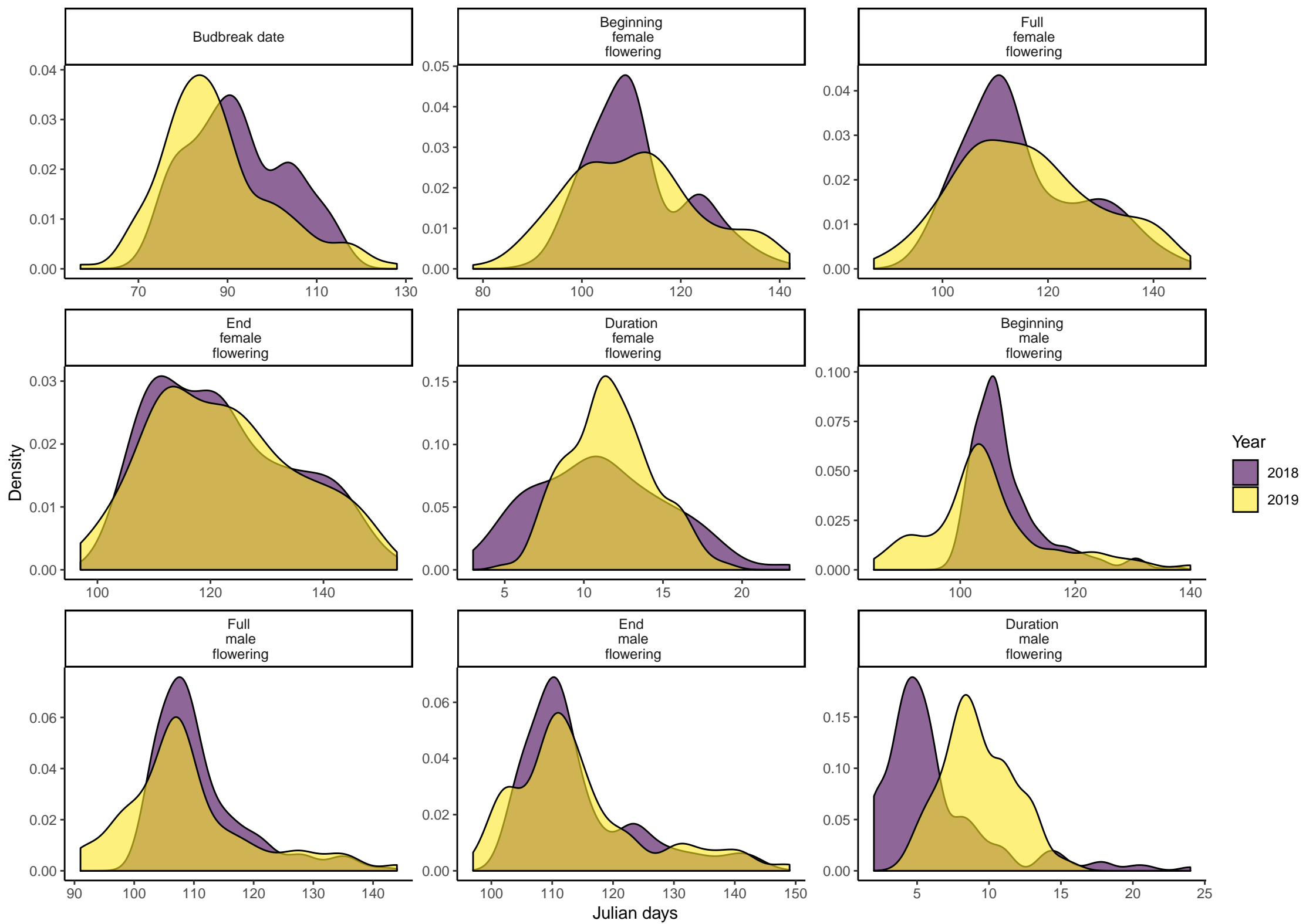

Supplement: Supplementary file 9 — Additional file 9: Figure S2. Density plots showing the two-year data related to phenological traits in Julian days for the 170 accessions of the GWAS panel. [file 12864_2020_6616_MOESM9_ESM.pdf]

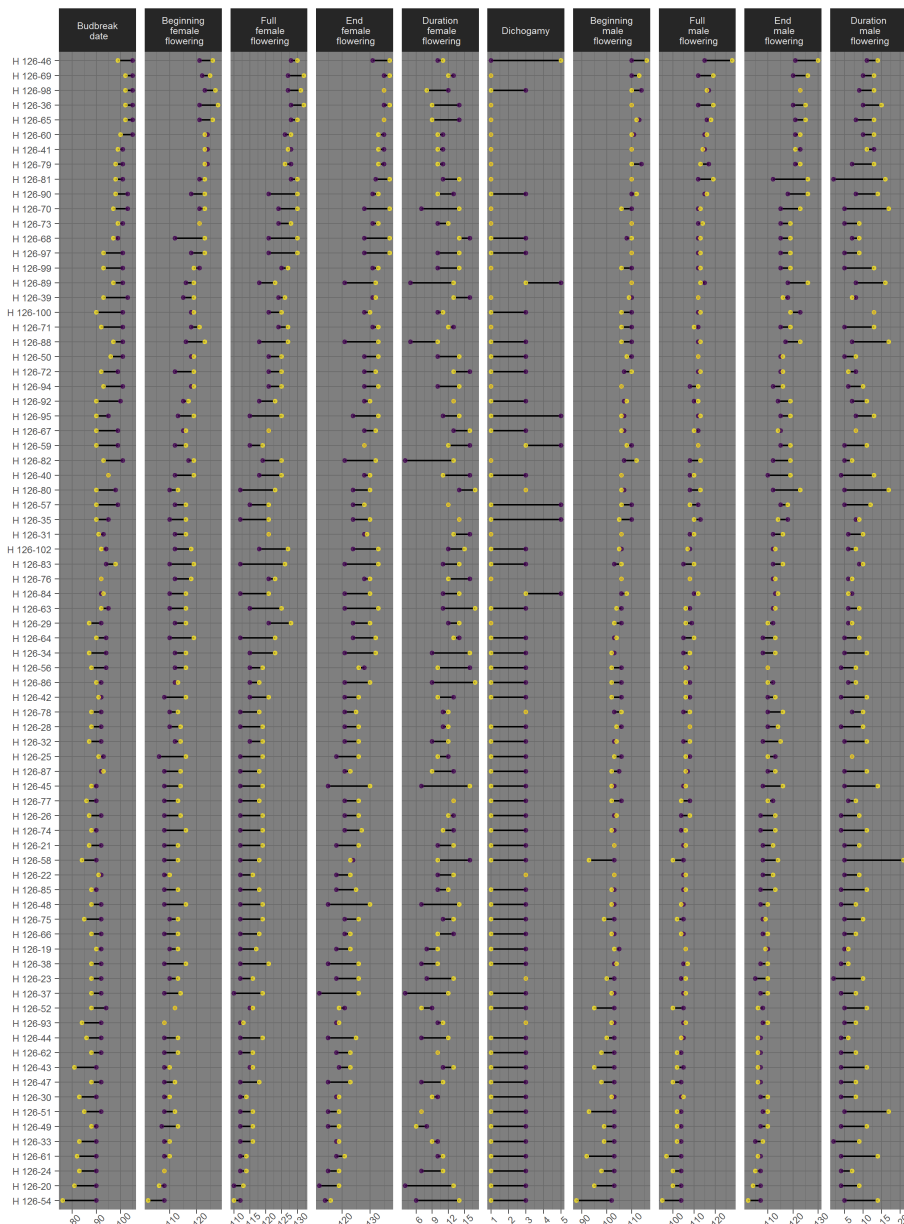

Years 2018 2019

Supplement: Supplementary file 10 — Additional file 10: Figure S3. Scatter plots showing the two-year data related to phenological traits in Julian days for the 78 accessions of the F1 progeny. [file 12864_2020_6616_MOESM10_ESM.pdf]

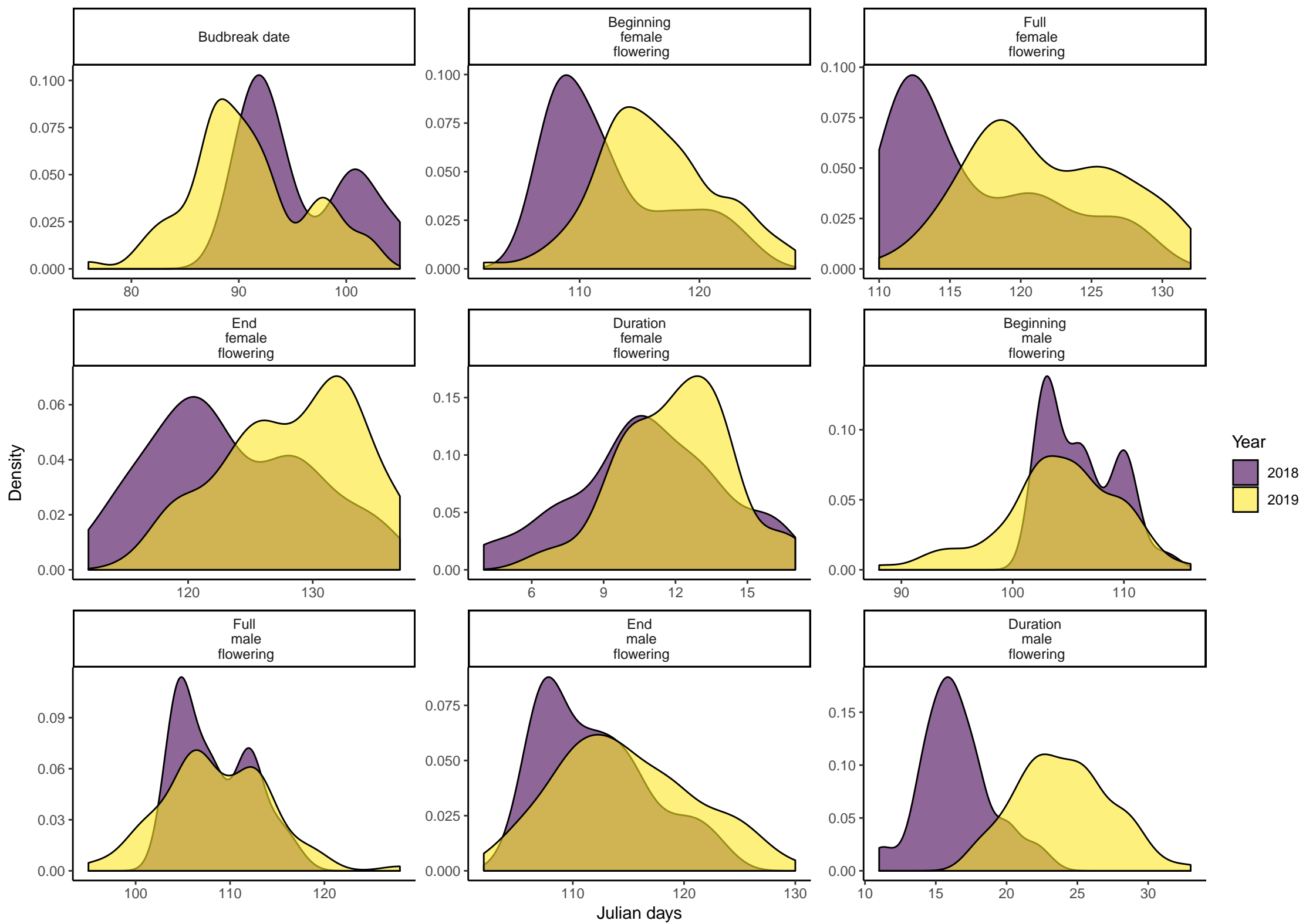

Supplement: Supplementary file 11 — Additional file 11: Figure S4. Density plots showing the two-year data related to phenological traits in Julian days for the 78 accessions of the F1 progeny. [file 12864_2020_6616_MOESM11_ESM.pdf]

a

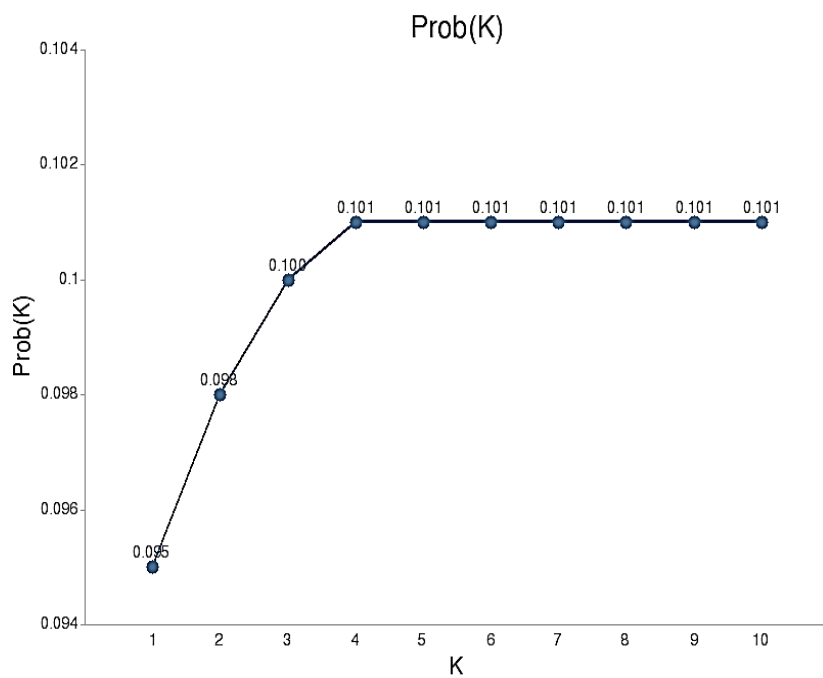

b

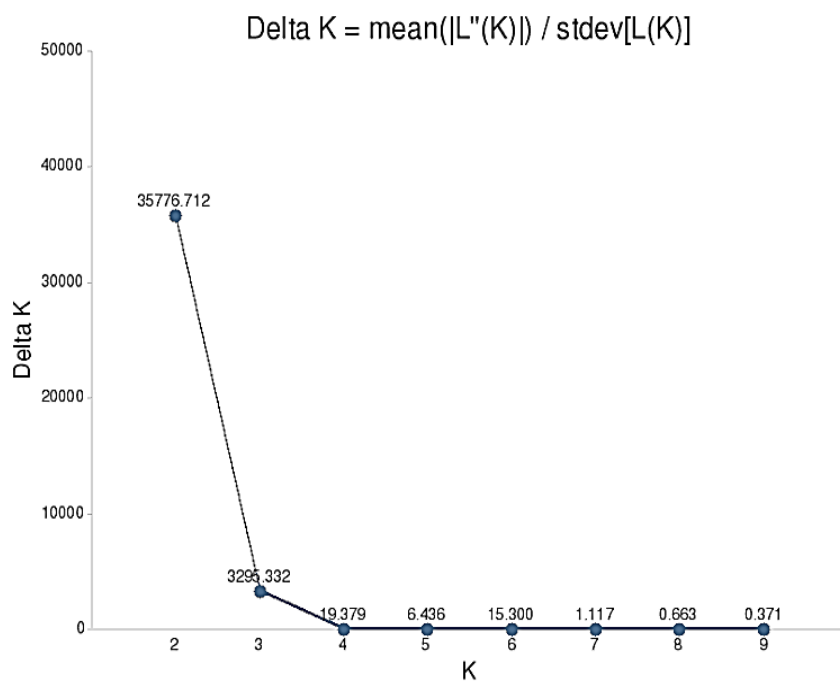

Supplement: Supplementary file 12 — Additional file 12: Figure S5. Detection of the number of clusters using a) Prob(K), and b) deltaK method (Evanno et al., 2005) in the GWAS panel. [file 12864_2020_6616_MOESM12_ESM.pdf]

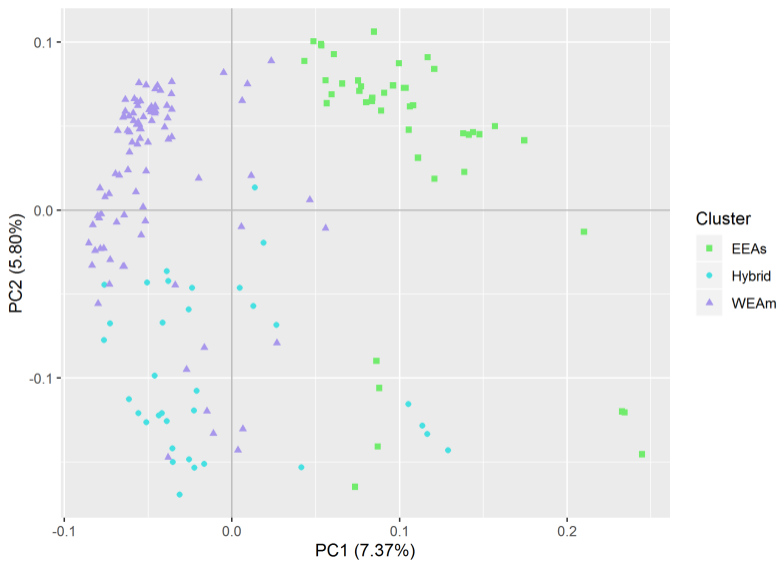

Supplement: Supplementary file 13 — Additional file 13: Figure S6. Principal Component Analysis performed on the GWAS panel. The two first principal components show accessions colored according to fastSTRUCTURE results with EEAs for Eastern Europe and Asia, and WEAm for Western Europe and America. [file 12864_2020_6616_MOESM13_ESM.pdf]

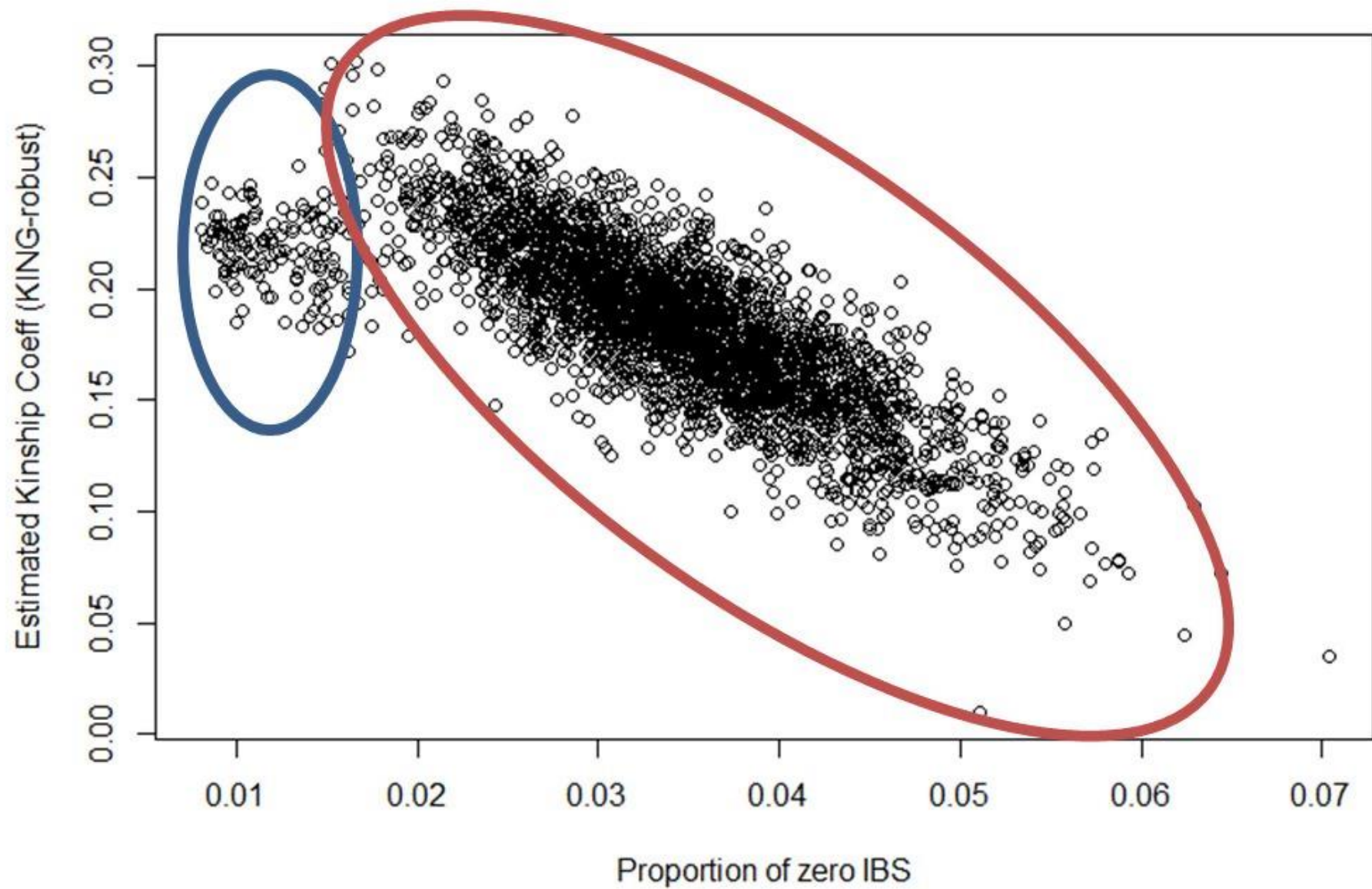

Supplement: Supplementary file 14 — Additional file 14: Figure S7. Scatter plot showing the estimated kinship coefficients by the proportion of zero Identical-By-State (IBS0) in the F1 progeny. [file 12864_2020_6616_MOESM14_ESM.pdf]

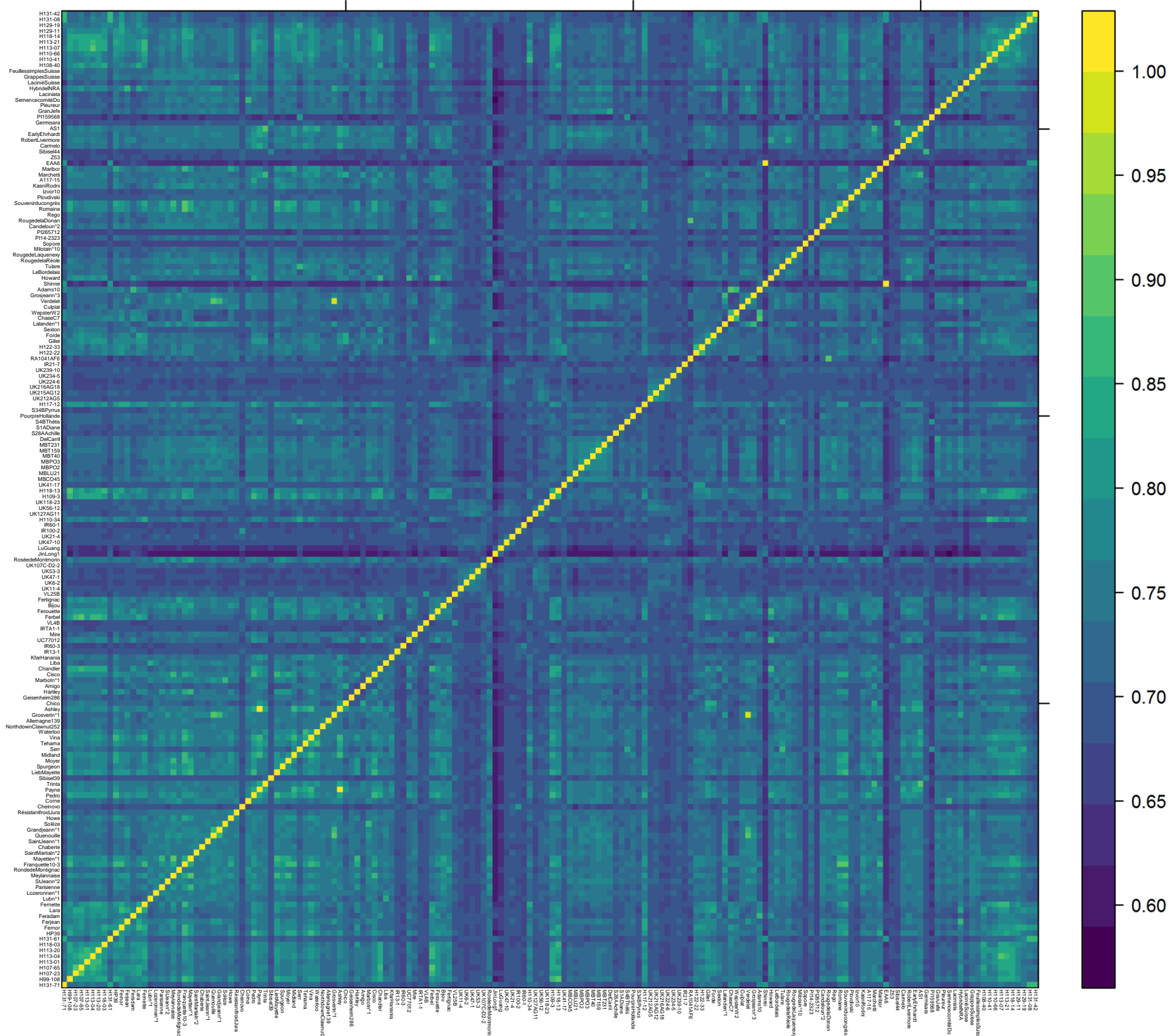

Supplement: Supplementary file 15 — Additional file 15: Figure S8. Level plot showing the Identical-By-State (IBS) values for the 170 accessions of the GWAS panel. [file 12864_2020_6616_MOESM15_ESM.pdf]
