# Supplementary material for: Association and linkage mapping to unravel genetic architecture of phenological traits and lateral bearing in Persian walnut (Juglans regia L.)
Source: BMC Genomics. 2020 Mar 4;21:203. doi: 10.1186/s12864-020-6616-y (PMC7057608; doi:10.1186/s12864-020-6616-y)
Supplement: Supplementary file 16 — Additional file 16: Figure S9. Genetic maps and QTLs detected using two-year data. F(X) and U(X) are the linkage groups of ‘Franquette’ and ‘UK 6–2’ parental maps respectively. Legend of the QTLs: black for budbreak date, red for beginning female flowering date, deep green for full female flowering date, blue for end female flowering date, yellow for beginning male flowering date, pink for full female flowering date, and light green for end female flowering date. Solid bars indicate the 95% confidence interval of the QTL, and terminal bars indicate the 99.9% confidence interval of the QTL. The marker names were changed with the corresponding chromosome number and its physical position for a better visualization. [file 12864_2020_6616_MOESM16_ESM.pdf]

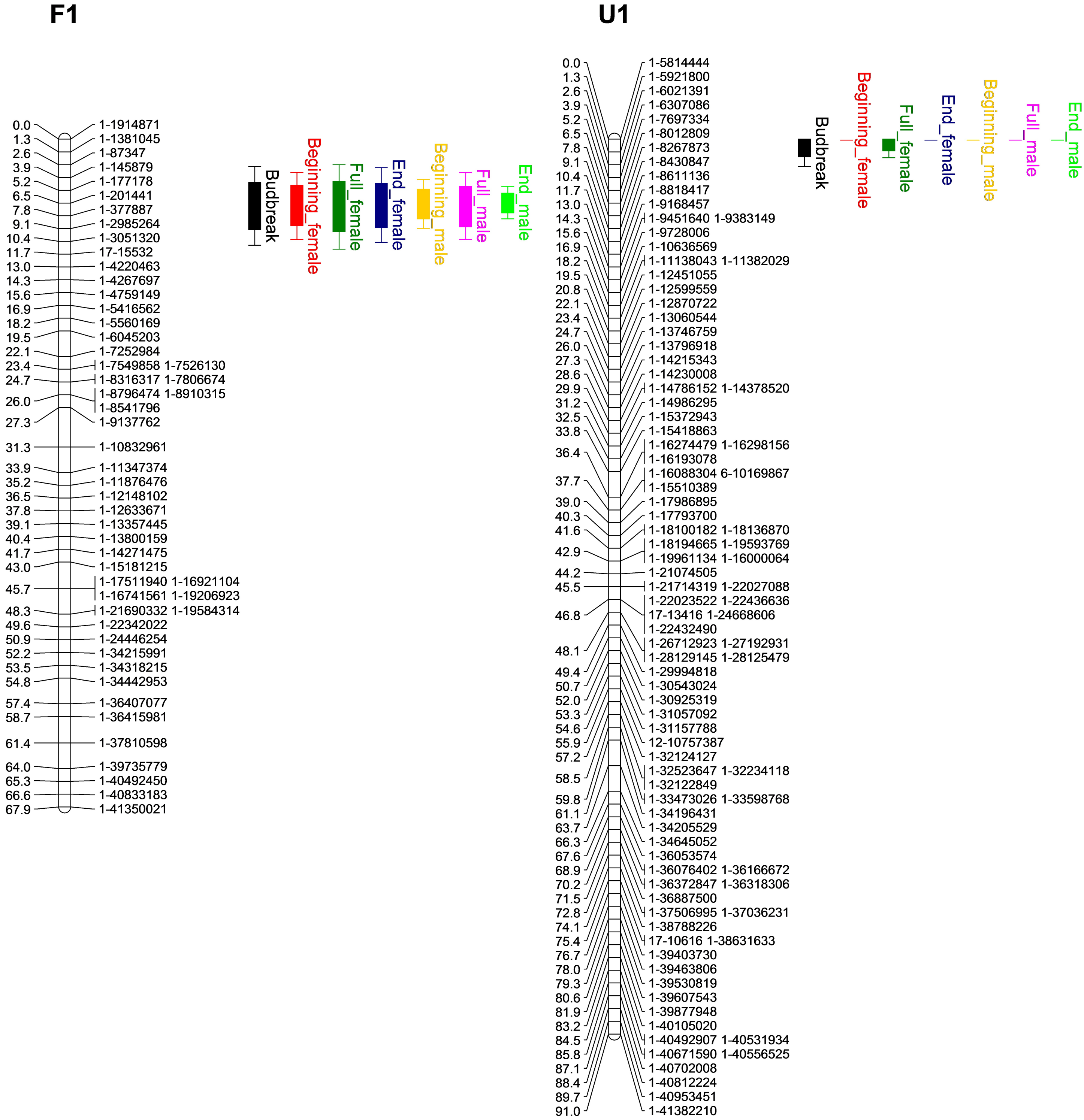
F1
0.0 1-1914871
1.3 1-1381045
2.6 1-87347
3.9 1-145879
5.2 1-177178
6.5 1-201441
7.8 1-377887
9.1 1-2985264
10.4 1-3051320
11.7 17-15532
13.0 1-4220463
14.3 1-4267697
15.6 1-4759149
16.9 1-5416562
18.2 1-5560169
19.5 1-6045203
22.1 1-7252984
23.4 1-7549858 1-7526130
24.7 1-8316317 1-7806674
26.0 1-8796474 1-8910315
1-8541796
27.3 1-9137762
31.3 1-10832961
33.9 1-11347374
35.2 1-11876476
36.5 1-12148102
37.8 1-12633671
39.1 1-13357445
40.4 1-13800159
41.7 1-14271475
43.0 1-15181215
45.7 1-17511940 1-16921104
1-16741561 1-19206923
48.3 1-21690332 1-19584314
49.6 1-22342022
50.9 1-24446254
52.2 1-34215991
53.5 1-34318215
54.8 1-34442953
57.4 1-36407077
58.7 1-36415981
61.4 1-37810598
64.0 1-39735779
65.3 1-40492450
66.6 1-40833183
67.9 1-41350021
Budbreak
Beginning_female
Full_female
End_female
Beginning_male
Full_male
End_male
U1
0.0 1-5814444
1.3 1-5921800
2.6 1-6021391
3.9 1-6307086
5.2 1-7697334
6.5 1-8012809
7.8 1-8267873
9.1 1-8430847
10.4 1-8611136
11.7 1-8818417
13.0 1-9168457
14.3 1-9451640 1-9383149
15.6 1-9728006
16.9 1-10636569
18.2 1-11138043 1-11382029
19.5 1-12451055
20.8 1-12599559
22.1 1-12870722
23.4 1-13060544
24.7 1-13746759
26.0 1-13796918
27.3 1-14215343
28.6 1-14230008
29.9 1-14786152 1-14378520
31.2 1-14986295
32.5 1-15372943
33.8 1-15418863
36.4 1-16274479 1-16298156
1-16193078
37.7 1-16088304 6-10169867
1-15510389
39.0 1-17986895
40.3 1-17793700
41.6 1-18100182 1-18136870
42.9 1-18194665 1-19593769
1-19961134 1-16000064
44.2 1-21074505
45.5 1-21714319 1-22027088
46.8 1-22023522 1-22436636
17-13416 1-24668606
1-22432490
48.1 1-26712923 1-27192931
1-28129145 1-28125479
49.4 1-29994818
50.7 1-30543024
52.0 1-30925319
53.3 1-31057092
54.6 1-31157788
55.9 12-10757387
57.2 1-32124127
58.5 1-32523647 1-32234118
1-32122849
59.8 1-33473026 1-33598768
61.1 1-34196431
63.7 1-34205529
66.3 1-34645052
67.6 1-36053574
68.9 1-36076402 1-36166672
70.2 1-36372847 1-36318306
71.5 1-36887500
72.8 1-37506995 1-37036231
74.1 1-38788226
75.4 17-10616 1-38631633
76.7 1-39403730
78.0 1-39463806
79.3 1-39530819
80.6 1-39607543
81.9 1-39877948
83.2 1-40105020
84.5 1-40492907 1-40531934
85.8 1-40671590 1-40556525
87.1 1-40702008
88.4 1-40812224
89.7 1-40953451
91.0 1-41382210
Budbreak
Beginning_female
Full_female
End_female
Beginning_male
Full_male
End_male

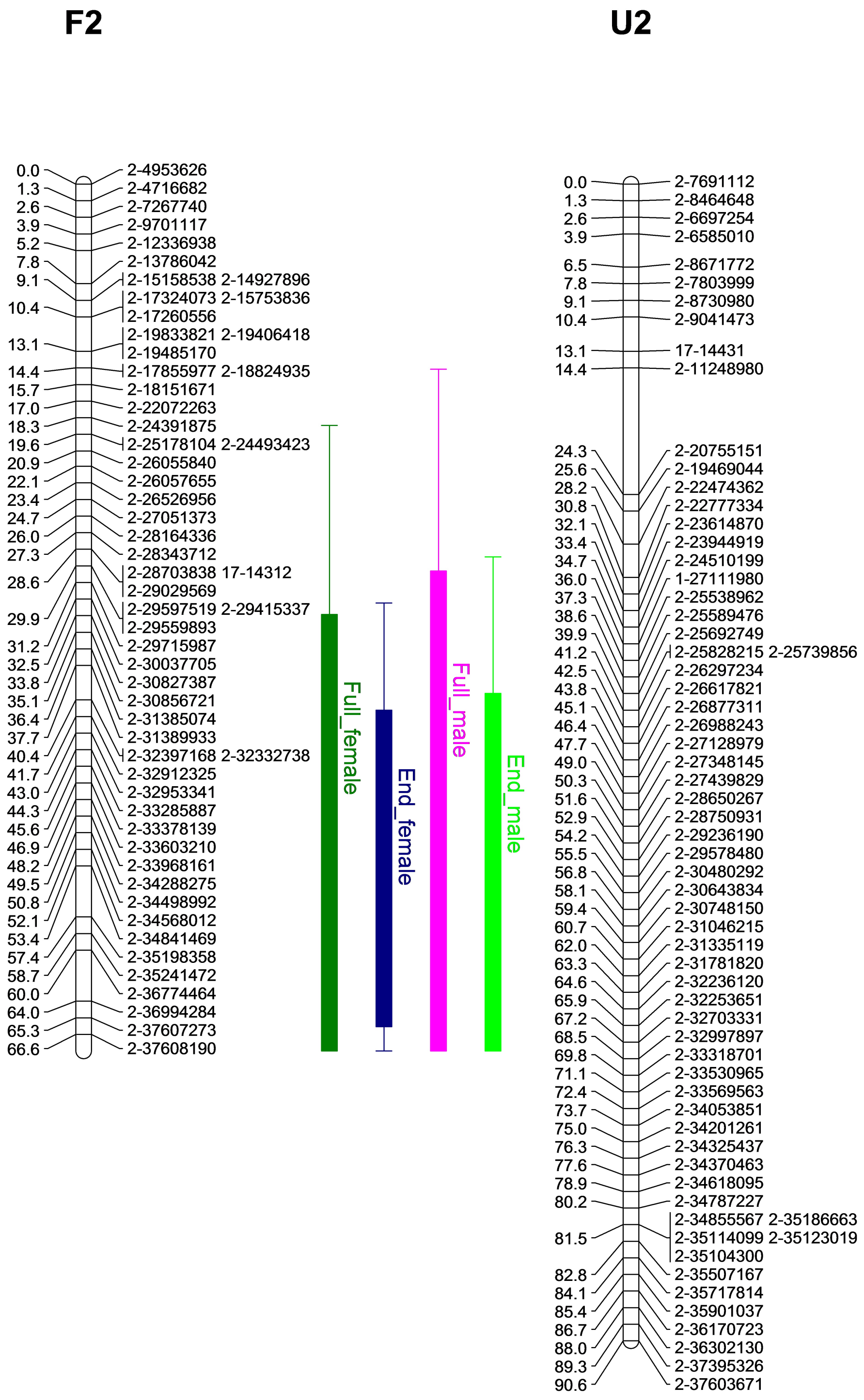
F2
U2
0.0 2-4953626
1.3 2-4716682
2.6 2-7267740
3.9 2-9701117
5.2 2-12336938
7.8 2-13786042
9.1 2-15158538 2-14927896
10.4 2-17324073 2-15753836
2-17260556
13.1 2-19833821 2-19406418
2-19485170
14.4 2-17855977 2-18824935
15.7 2-18151671
17.0 2-22072263
18.3 2-24391875
19.6 2-25178104 2-24493423
20.9 2-26055840
22.1 2-26057655
23.4 2-26526956
24.7 2-27051373
26.0 2-28164336
27.3 2-28343712
28.6 2-28703838 17-14312
2-29029569
29.9 2-29597519 2-29415337
2-29559893
31.2 2-29715987
32.5 2-30037705
33.8 2-30827387
35.1 2-30856721
36.4 2-31385074
37.7 2-31389933
40.4 2-32397168 2-32332738
41.7 2-32912325
43.0 2-32953341
44.3 2-33285887
45.6 2-33378139
46.9 2-33603210
48.2 2-33968161
49.5 2-34288275
50.8 2-34498992
52.1 2-34568012
53.4 2-34841469
57.4 2-35198358
58.7 2-35241472
60.0 2-36774464
64.0 2-36994284
65.3 2-37607273
66.6 2-37608190
Full_female
End_female
Full_male
End_male
0.0 2-7691112
1.3 2-8464648
2.6 2-6697254
3.9 2-6585010
6.5 2-8671772
7.8 2-7803999
9.1 2-8730980
10.4 2-9041473
13.1 17-14431
14.4 2-11248980
24.3 2-20755151
25.6 2-19469044
28.2 2-22474362
30.8 2-22777334
32.1 2-23614870
33.4 2-23944919
34.7 2-24510199
36.0 1-27111980
37.3 2-25538962
38.6 2-25589476
39.9 2-25692749
41.2 2-25828215 2-25739856
42.5 2-26297234
43.8 2-26617821
45.1 2-26877311
46.4 2-26988243
47.7 2-27128979
49.0 2-27348145
50.3 2-27439829
51.6 2-28650267
52.9 2-28750931
54.2 2-29236190
55.5 2-29578480
56.8 2-30480292
58.1 2-30643834
59.4 2-30748150
60.7 2-31046215
62.0 2-31335119
63.3 2-31781820
64.6 2-32236120
65.9 2-32253651
67.2 2-32703331
68.5 2-32997897
69.8 2-33318701
71.1 2-33530965
72.4 2-33569563
73.7 2-34053851
75.0 2-34201261
76.3 2-34325437
77.6 2-34370463
78.9 2-34618095
80.2 2-34787227
81.5 2-34855567 2-35186663
2-35114099 2-35123019
2-35104300
82.8 2-35507167
84.1 2-35717814
85.4 2-35901037
86.7 2-36170723
88.0 2-36302130
89.3 2-37395326
90.6 2-37603671

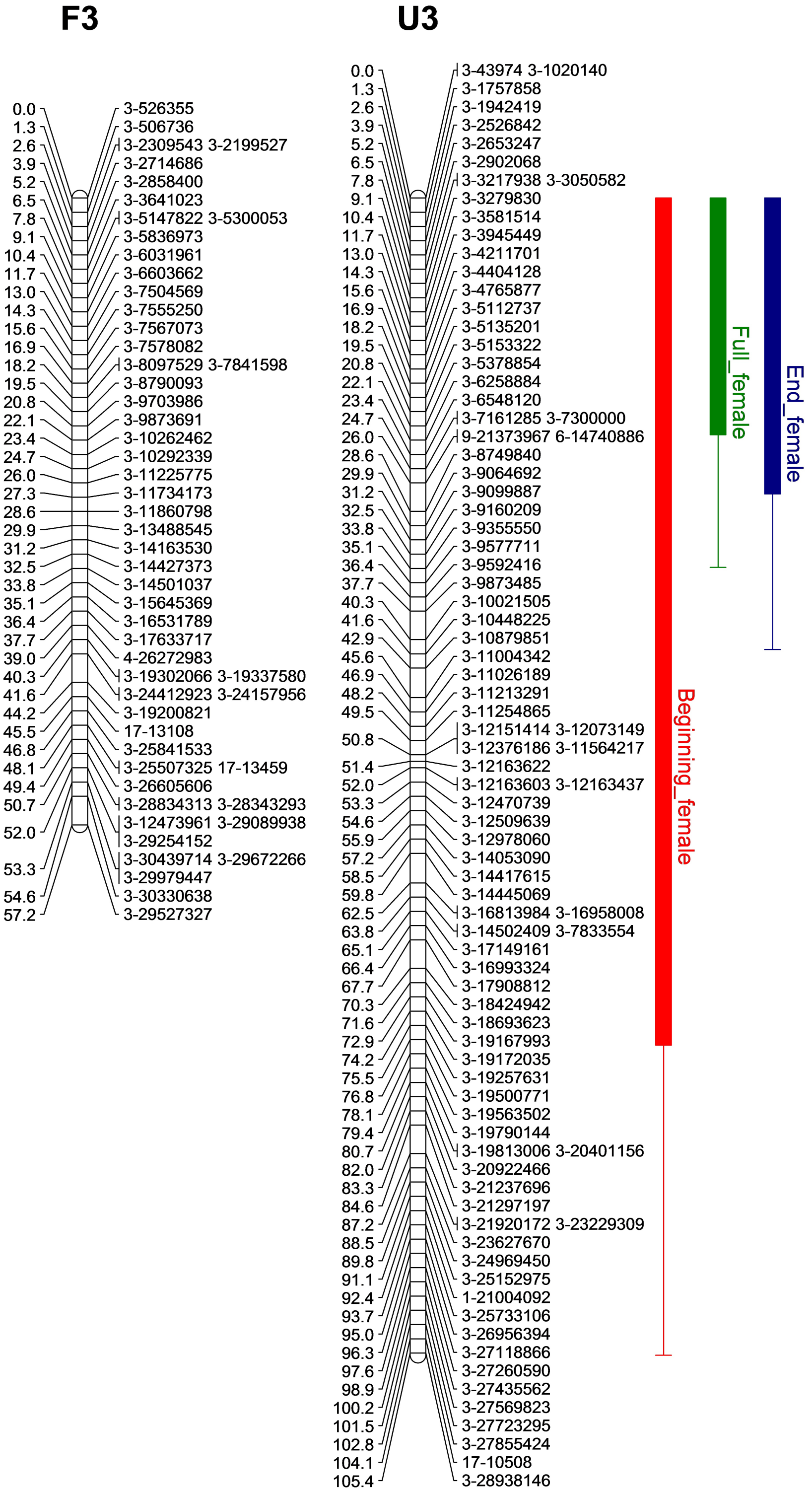
F3
U3
0.0
1.3
2.6
3.9
5.2
6.5
7.8
9.1
10.4
11.7
13.0
14.3
15.6
16.9
18.2
19.5
20.8
22.1
23.4
24.7
26.0
27.3
28.6
29.9
31.2
32.5
33.8
35.1
36.4
37.7
39.0
40.3
41.6
44.2
45.5
46.8
48.1
49.4
50.7
52.0
53.3
54.6
57.2
3-526355
3-506736
3-2309543 3-2199527
3-2714686
3-2858400
3-3641023
3-5147822 3-5300053
3-5836973
3-6031961
3-6603662
3-7504569
3-7555250
3-7567073
3-7578082
3-8097529 3-7841598
3-8790093
3-9703986
3-9873691
3-10262462
3-10292339
3-11225775
3-11734173
3-11860798
3-13488545
3-14163530
3-14427373
3-14501037
3-15645369
3-16531789
3-17633717
4-26272983
3-19302066 3-19337580
3-24412923 3-24157956
3-19200821
17-13108
3-25841533
3-25507325 17-13459
3-26605606
3-28834313 3-28343293
3-12473961 3-29089938
3-29254152
3-30439714 3-29672266
3-29979447
3-30330638
3-29527327
0.0
1.3
2.6
3.9
5.2
6.5
7.8
9.1
10.4
11.7
13.0
14.3
15.6
16.9
18.2
19.5
20.8
22.1
23.4
24.7
26.0
28.6
29.9
31.2
32.5
33.8
35.1
36.4
37.7
40.3
41.6
42.9
45.6
46.9
48.2
49.5
50.8
51.4
52.0
53.3
54.6
55.9
57.2
58.5
59.8
62.5
63.8
65.1
66.4
67.7
70.3
71.6
72.9
74.2
75.5
76.8
78.1
79.4
80.7
82.0
83.3
84.6
87.2
88.5
89.8
91.1
92.4
93.7
95.0
96.3
97.6
98.9
100.2
101.5
102.8
104.1
105.4
3-43974 3-1020140
3-1757858
3-1942419
3-2526842
3-2653247
3-2902068
3-3217938 3-3050582
3-3279830
3-3581514
3-3945449
3-4211701
3-4404128
3-4765877
3-5112737
3-5135201
3-5153322
3-5378854
3-6258884
3-6548120
3-7161285 3-7300000
9-21373967 6-14740886
3-8749840
3-9064692
3-9099887
3-9160209
3-9355550
3-9577711
3-9592416
3-9873485
3-10021505
3-10448225
3-10879851
3-11004342
3-11026189
3-11213291
3-11254865
3-12151414 3-12073149
3-12376186 3-11564217
3-12163622
3-12163603 3-12163437
3-12470739
3-12509639
3-12978060
3-14053090
3-14417615
3-14445069
3-16813984 3-16958008
3-14502409 3-7833554
3-17149161
3-16993324
3-17908812
3-18424942
3-18693623
3-19167993
3-19172035
3-19257631
3-19500771
3-19563502
3-19790144
3-19813006 3-20401156
3-20922466
3-21237696
3-21297197
3-21920172 3-23229309
3-23627670
3-24969450
3-25152975
1-21004092
3-25733106
3-26956394
3-27118866
3-27260590
3-27435562
3-27569823
3-27723295
3-27855424
17-10508
3-28938146
Beginning_female
Full_female
End_female

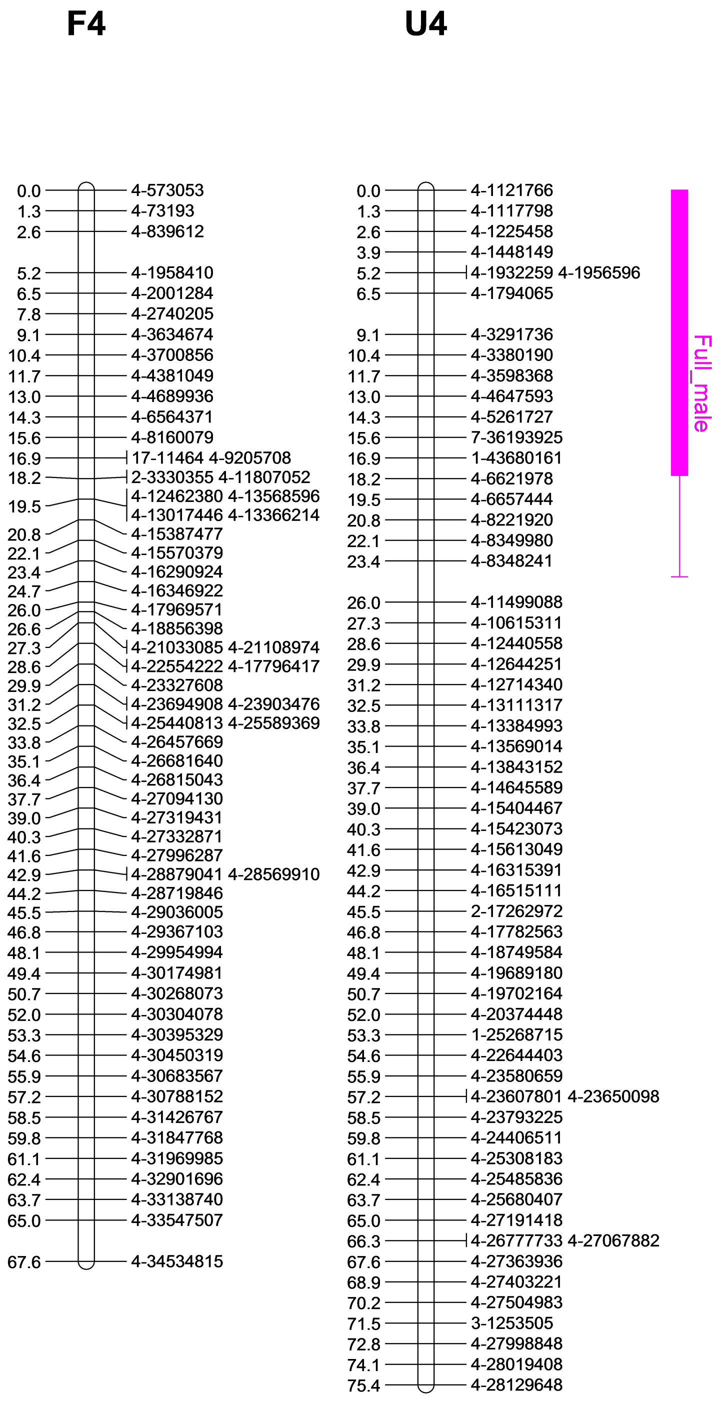

F4
U4
0.0 4-573053
1.3 4-73193
2.6 4-839612
5.2 4-1958410
6.5 4-2001284
7.8 4-2740205
9.1 4-3634674
10.4 4-3700856
11.7 4-4381049
13.0 4-4689936
14.3 4-6564371
15.6 4-8160079
16.9 17-11464 4-9205708
18.2 2-3330355 4-11807052
19.5 4-12462380 4-13568596 4-13017446 4-13366214
20.8 4-15387477
22.1 4-15570379
23.4 4-16290924
24.7 4-16346922
26.0 4-17969571
26.6 4-18856398
27.3 4-21033085 4-21108974
28.6 4-22554222 4-17796417
29.9 4-23327608
31.2 4-23694908 4-23903476
32.5 4-25440813 4-25589369
33.8 4-26457669
35.1 4-26681640
36.4 4-26815043
37.7 4-27094130
39.0 4-27319431
40.3 4-27332871
41.6 4-27996287
42.9 4-28879041 4-28569910
44.2 4-28719846
45.5 4-29036005
46.8 4-29367103
48.1 4-29954994
49.4 4-30174981
50.7 4-30268073
52.0 4-30304078
53.3 4-30395329
54.6 4-30450319
55.9 4-30683567
57.2 4-30788152
58.5 4-31426767
59.8 4-31847768
61.1 4-31969985
62.4 4-32901696
63.7 4-33138740
65.0 4-33547507
67.6 4-34534815
0.0 4-1121766
1.3 4-1117798
2.6 4-1225458
3.9 4-1448149
5.2 4-1932259 4-1956596
6.5 4-1794065
9.1 4-3291736
10.4 4-3380190
11.7 4-3598368
13.0 4-4647593
14.3 4-5261727
15.6 7-36193925
16.9 1-43680161
18.2 4-6621978
19.5 4-6657444
20.8 4-8221920
22.1 4-8349980
23.4 4-8348241
26.0 4-11499088
27.3 4-10615311
28.6 4-12440558
29.9 4-12644251
31.2 4-12714340
32.5 4-13111317
33.8 4-13384993
35.1 4-13569014
36.4 4-13843152
37.7 4-14645589
39.0 4-15404467
40.3 4-15423073
41.6 4-15613049
42.9 4-16315391
44.2 4-16515111
45.5 2-17262972
46.8 4-17782563
48.1 4-18749584
49.4 4-19689180
50.7 4-19702164
52.0 4-20374448
53.3 1-25268715
54.6 4-22644403
55.9 4-23580659
57.2 4-23607801 4-23650098
58.5 4-23793225
59.8 4-24406511
61.1 4-25308183
62.4 4-25485836
63.7 4-25680407
65.0 4-27191418
66.3 4-26777733 4-27067882
67.6 4-27363936
68.9 4-27403221
70.2 4-27504983
71.5 3-1253505
72.8 4-27998848
74.1 4-28019408
75.4 4-28129648
Full_male

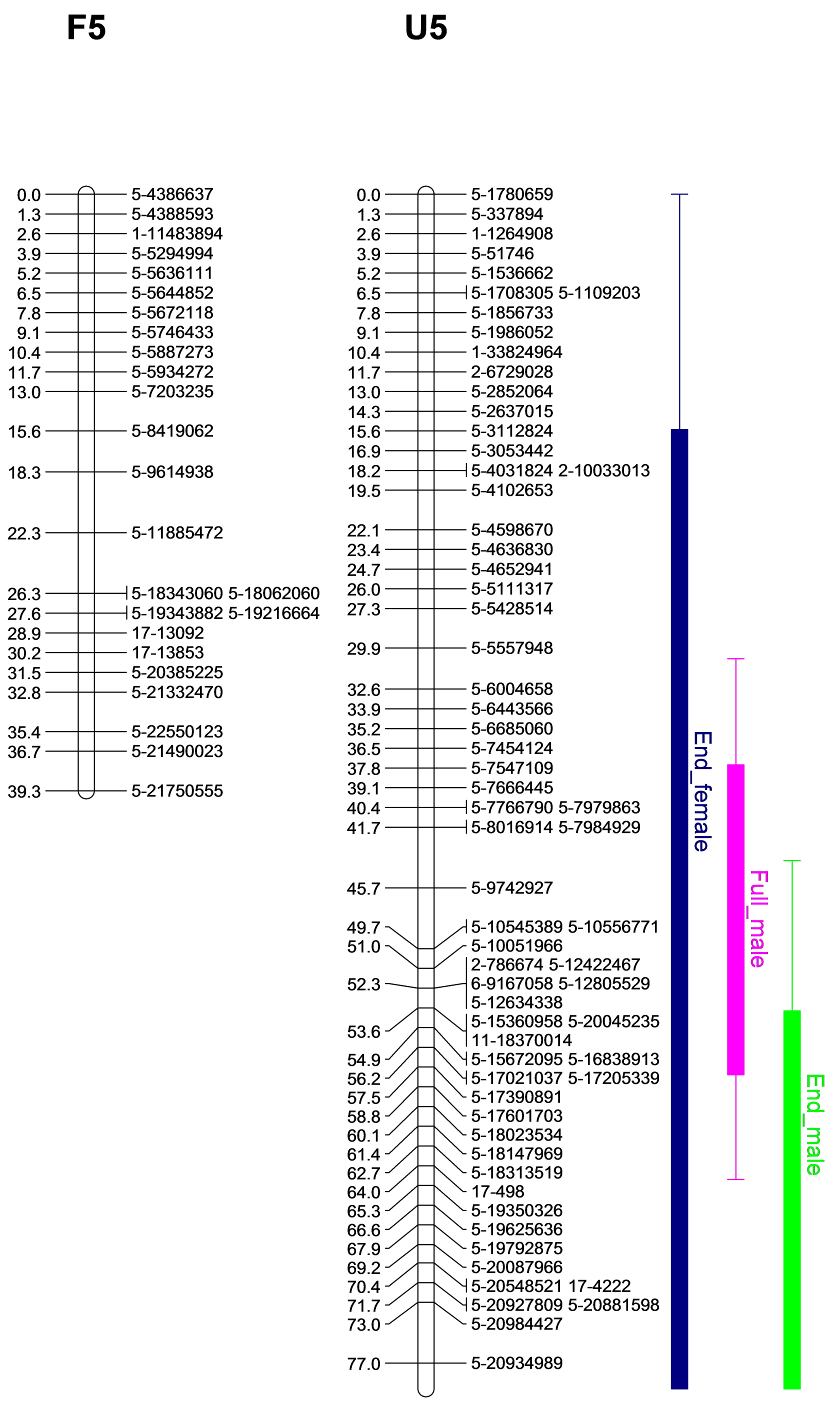
F5
U5
0.0 5-4386637
1.3 5-4388593
2.6 1-11483894
3.9 5-5294994
5.2 5-5636111
6.5 5-5644852
7.8 5-5672118
9.1 5-5746433
10.4 5-5887273
11.7 5-5934272
13.0 5-7203235
15.6 5-8419062
18.3 5-9614938
22.3 5-11885472
26.3 5-18343060 5-18062060
27.6 5-19343882 5-19216664
28.9 17-13092
30.2 17-13853
31.5 5-20385225
32.8 5-21332470
35.4 5-22550123
36.7 5-21490023
39.3 5-21750555
0.0 5-1780659
1.3 5-337894
2.6 1-1264908
3.9 5-51746
5.2 5-1536662
6.5 5-1708305 5-1109203
7.8 5-1856733
9.1 5-1986052
10.4 1-33824964
11.7 2-6729028
13.0 5-2852064
14.3 5-2637015
15.6 5-3112824
16.9 5-3053442
18.2 5-4031824 2-10033013
19.5 5-4102653
22.1 5-4598670
23.4 5-4636830
24.7 5-4652941
26.0 5-5111317
27.3 5-5428514
29.9 5-5557948
32.6 5-6004658
33.9 5-6443566
35.2 5-6685060
36.5 5-7454124
37.8 5-7547109
39.1 5-7666445
40.4 5-7766790 5-7979863
41.7 5-8016914 5-7984929
45.7 5-9742927
49.7 5-10545389 5-10556771
51.0 5-10051966
52.3 2-786674 5-12422467 6-9167058 5-12805529 5-12634338
53.6 5-15360958 5-20045235 11-18370014
54.9 5-15672095 5-16838913
56.2 5-17021037 5-17205339
57.5 5-17390891
58.8 5-17601703
60.1 5-18023534
61.4 5-18147969
62.7 5-18313519
64.0 17-498
65.3 5-19350326
66.6 5-19625636
67.9 5-19792875
69.2 5-20087966
70.4 5-20548521 17-4222
71.7 5-20927809 5-20881598
73.0 5-20984427
77.0 5-20934989
End_female
Full_male
End_male

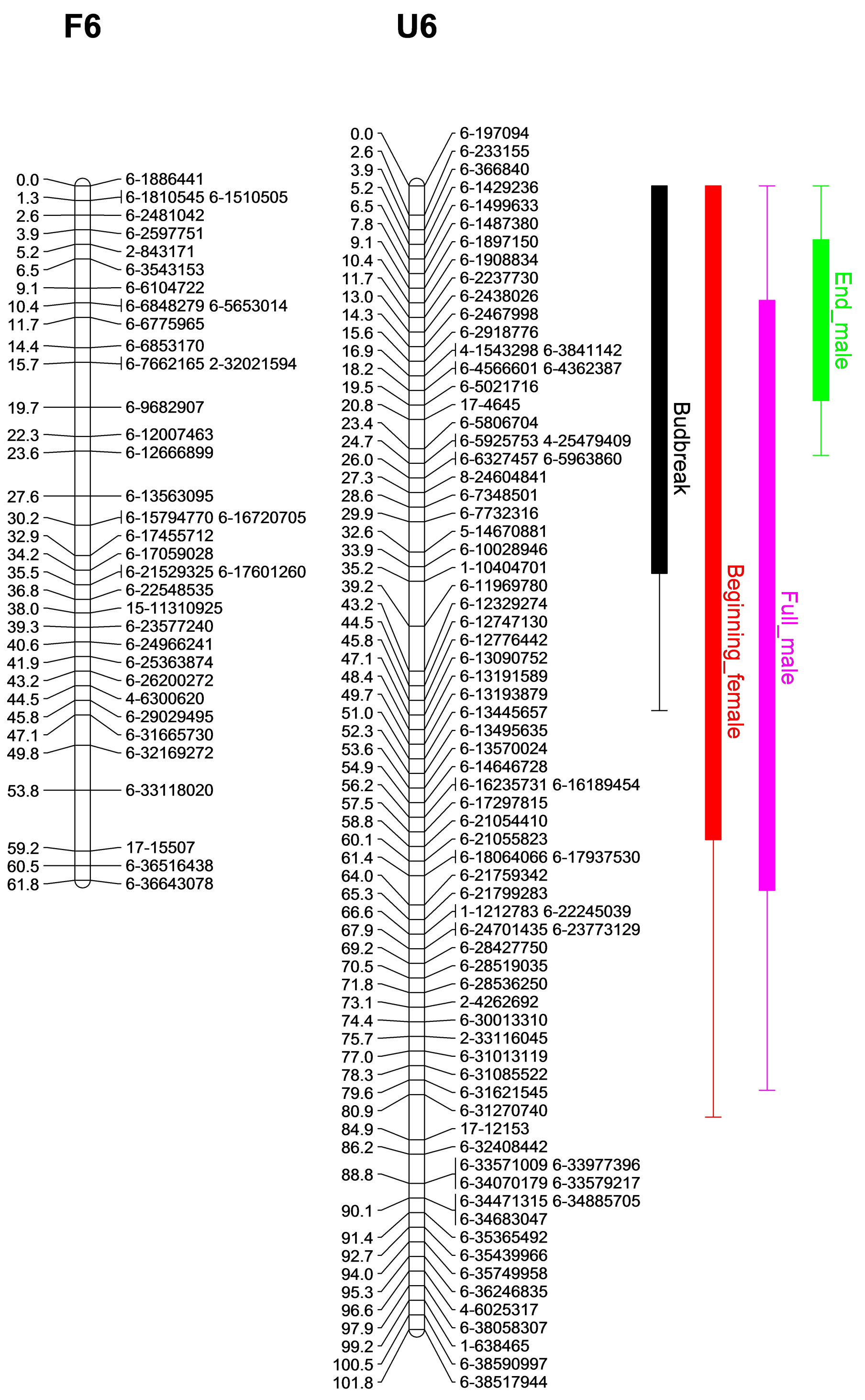
F6
U6
Budbreak
Beginning_female
Full_male
End_male

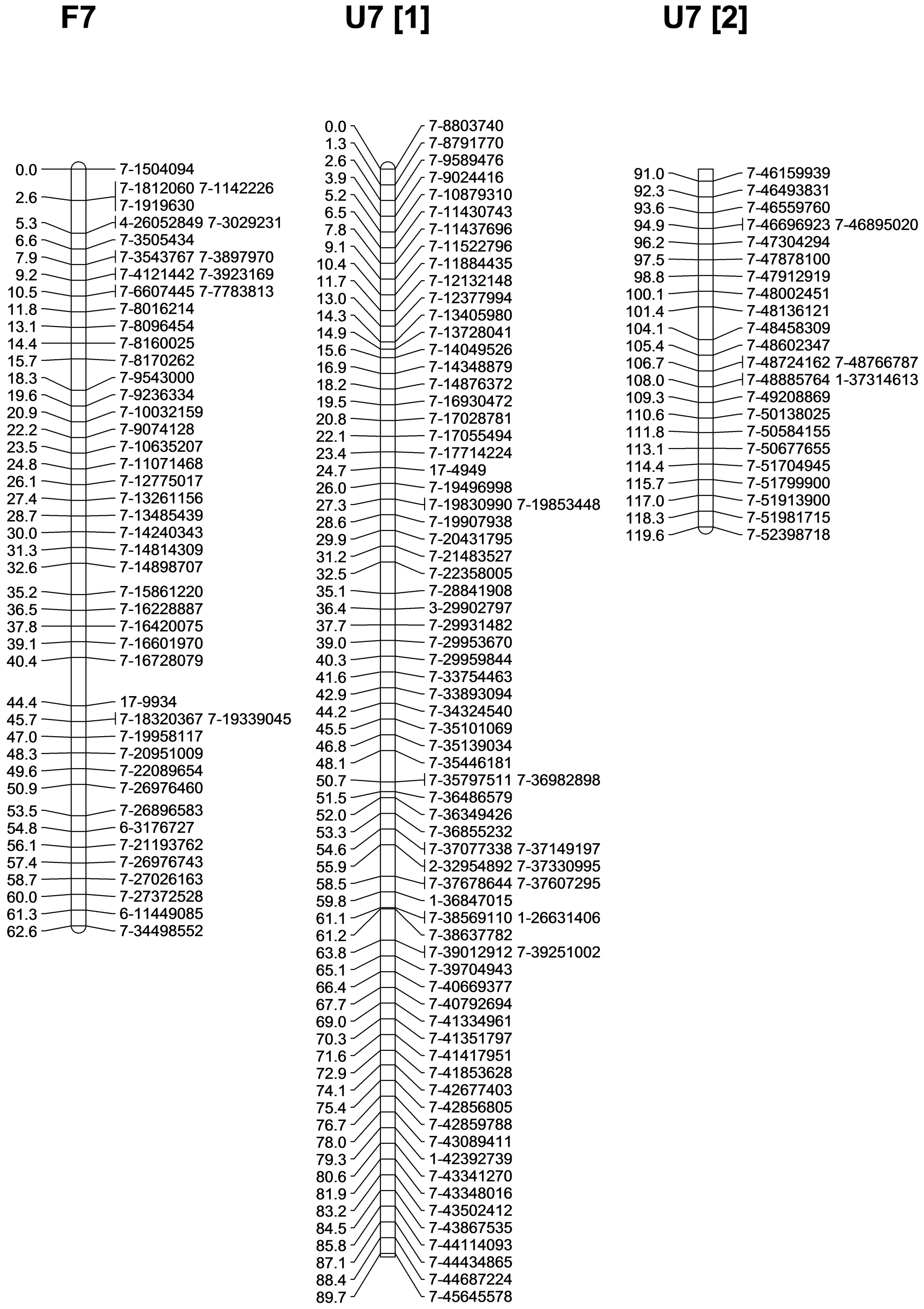

F7
0.0 7-1504094
2.6 7-1812060 7-1142226
7-1919630
5.3 4-26052849 7-3029231
6.6 7-3505434
7.9 7-3543767 7-3897970
9.2 7-4121442 7-3923169
10.5 7-6607445 7-7783813
11.8 7-8016214
13.1 7-8096454
14.4 7-8160025
15.7 7-8170262
18.3 7-9543000
19.6 7-9236334
20.9 7-10032159
22.2 7-9074128
23.5 7-10635207
24.8 7-11071468
26.1 7-12775017
27.4 7-13261156
28.7 7-13485439
30.0 7-14240343
31.3 7-14814309
32.6 7-14898707
35.2 7-15861220
36.5 7-16228887
37.8 7-16420075
39.1 7-16601970
40.4 7-16728079
44.4 17-9934
45.7 7-18320367 7-19339045
47.0 7-19958117
48.3 7-20951009
49.6 7-22089654
50.9 7-26976460
53.5 7-26896583
54.8 6-3176727
56.1 7-21193762
57.4 7-26976743
58.7 7-27026163
60.0 7-27372528
61.3 6-11449085
62.6 7-34498552
U7 [1]
0.0 7-8803740
1.3 7-8791770
2.6 7-9589476
3.9 7-9024416
5.2 7-10879310
6.5 7-11430743
7.8 7-11437696
9.1 7-11522796
10.4 7-11884435
11.7 7-12132148
13.0 7-12377994
14.3 7-13405980
14.9 7-13728041
15.6 7-14049526
16.9 7-14348879
18.2 7-14876372
19.5 7-16930472
20.8 7-17028781
22.1 7-17055494
23.4 7-17714224
24.7 17-4949
26.0 7-19496998
27.3 7-19830990 7-19853448
28.6 7-19907938
29.9 7-20431795
31.2 7-21483527
32.5 7-22358005
35.1 7-28841908
36.4 3-29902797
37.7 7-29931482
39.0 7-29953670
40.3 7-29959844
41.6 7-33754463
42.9 7-33893094
44.2 7-34324540
45.5 7-35101069
46.8 7-35139034
48.1 7-35446181
50.7 7-35797511 7-36982898
51.5 7-36486579
52.0 7-36349426
53.3 7-36855232
54.6 7-37077338 7-37149197
55.9 2-32954892 7-37330995
58.5 7-37678644 7-37607295
59.8 1-36847015
61.1 7-38569110 1-26631406
61.2 7-38637782
63.8 7-39012912 7-39251002
65.1 7-39704943
66.4 7-40669377
67.7 7-40792694
69.0 7-41334961
70.3 7-41351797
71.6 7-41417951
72.9 7-41853628
74.1 7-42677403
75.4 7-42856805
76.7 7-42859788
78.0 7-43089411
79.3 1-42392739
80.6 7-43341270
81.9 7-43348016
83.2 7-43502412
84.5 7-43867535
85.8 7-44114093
87.1 7-44434865
88.4 7-44687224
89.7 7-45645578
U7 [2]
91.0 7-46159939
92.3 7-46493831
93.6 7-46559760
94.9 7-46696923 7-46895020
96.2 7-47304294
97.5 7-47878100
98.8 7-47912919
100.1 7-48002451
101.4 7-48136121
104.1 7-48458309
105.4 7-48602347
106.7 7-48724162 7-48766787
108.0 7-48885764 1-37314613
109.3 7-49208869
110.6 7-50138025
111.8 7-50584155
113.1 7-50677655
114.4 7-51704945
115.7 7-51799900
117.0 7-51913900
118.3 7-51981715
119.6 7-52398718

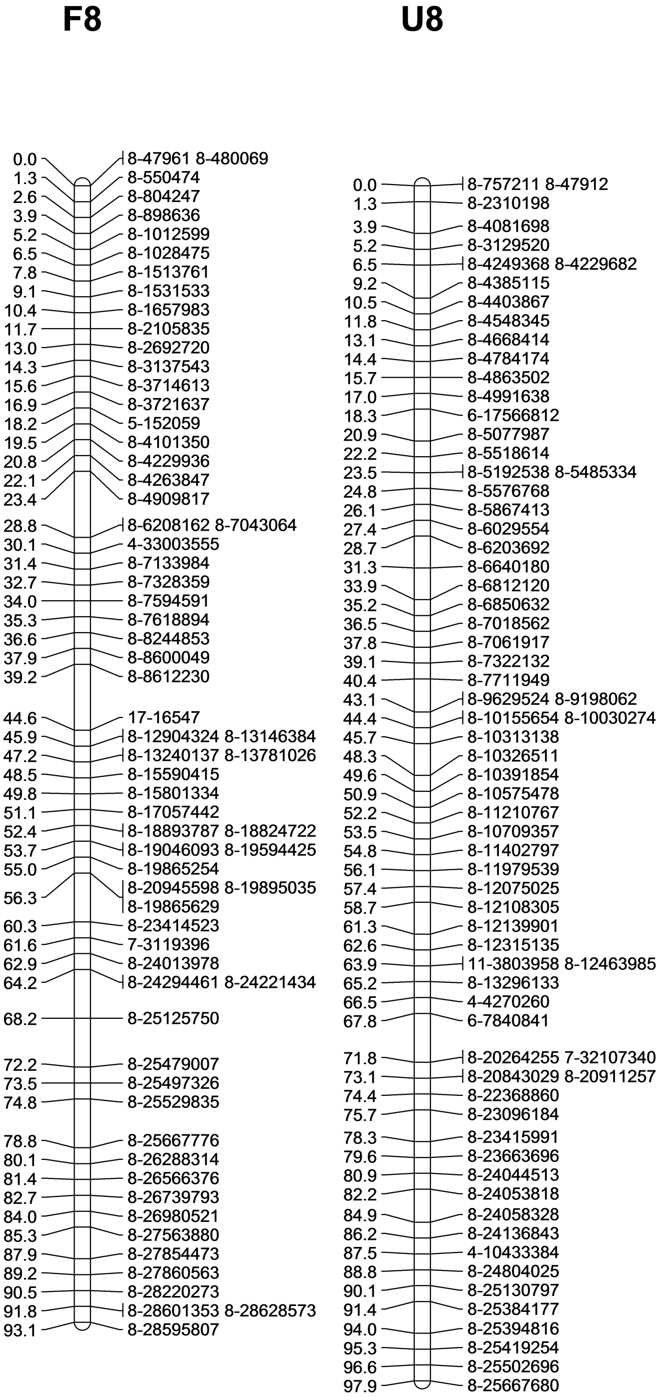
F8
0.0 8-47961 8-480069
1.3 8-550474
2.6 8-804247
3.9 8-898636
5.2 8-1012599
6.5 8-1028475
7.8 8-1513761
9.1 8-1531533
10.4 8-1657983
11.7 8-2105835
13.0 8-2692720
14.3 8-3137543
15.6 8-3714613
16.9 8-3721637
18.2 5-152059
19.5 8-4101350
20.8 8-4229936
22.1 8-4263847
23.4 8-4909817
28.8 8-6208162 8-7043064
30.1 4-33003555
31.4 8-7133984
32.7 8-7328359
34.0 8-7594591
35.3 8-7618894
36.6 8-8244853
37.9 8-8600049
39.2 8-8612230
44.6 17-16547
45.9 8-12904324 8-13146384
47.2 8-13240137 8-13781026
48.5 8-15590415
49.8 8-15801334
51.1 8-17057442
52.4 8-18893787 8-18824722
53.7 8-19046093 8-19594425
55.0 8-19865254
56.3 8-20945598 8-19895035
8-19865629
60.3 8-23414523
61.6 7-3119396
62.9 8-24013978
64.2 8-24294461 8-24221434
68.2 8-25125750
72.2 8-25479007
73.5 8-25497326
74.8 8-25529835
78.8 8-25667776
80.1 8-26288314
81.4 8-26566376
82.7 8-26739793
84.0 8-26980521
85.3 8-27563880
87.9 8-27854473
89.2 8-27860563
90.5 8-28220273
91.8 8-28601353 8-28628573
93.1 8-28595807
U8
0.0 8-757211 8-47912
1.3 8-2310198
3.9 8-4081698
5.2 8-3129520
6.5 8-4249368 8-4229682
9.2 8-4385115
10.5 8-4403867
11.8 8-4548345
13.1 8-4668414
14.4 8-4784174
15.7 8-4863502
17.0 8-4991638
18.3 6-17566812
20.9 8-5077987
22.2 8-5518614
23.5 8-5192538 8-5485334
24.8 8-5576768
26.1 8-5867413
27.4 8-6029554
28.7 8-6203692
31.3 8-6640180
33.9 8-6812120
35.2 8-6850632
36.5 8-7018562
37.8 8-7061917
39.1 8-7322132
40.4 8-7711949
43.1 8-9629524 8-9198062
44.4 8-10155654 8-10030274
45.7 8-10313138
48.3 8-10326511
49.6 8-10391854
50.9 8-10575478
52.2 8-11210767
53.5 8-10709357
54.8 8-11402797
56.1 8-11979539
57.4 8-12075025
58.7 8-12108305
61.3 8-12139901
62.6 8-12315135
63.9 11-3803958 8-12463985
65.2 8-13296133
66.5 4-4270260
67.8 6-7840841
71.8 8-20264255 7-32107340
73.1 8-20843029 8-20911257
74.4 8-22368860
75.7 8-23096184
78.3 8-23415991
79.6 8-23663696
80.9 8-24044513
82.2 8-24053818
84.9 8-24058328
86.2 8-24136843
87.5 4-10433384
88.8 8-24804025
90.1 8-25130797
91.4 8-25384177
94.0 8-25394816
95.3 8-25419254
96.6 8-25502696
97.9 8-25667680

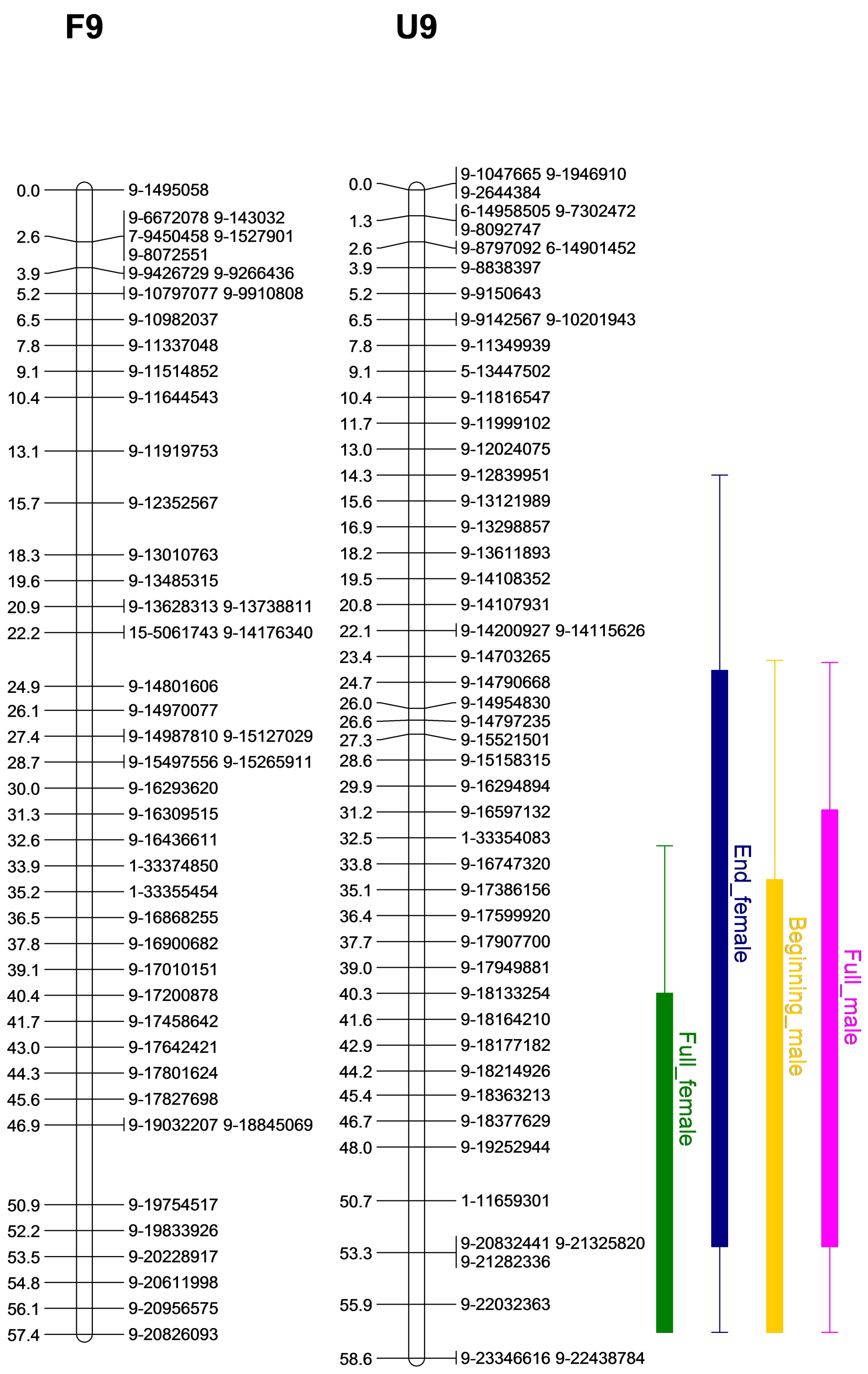
F9
U9
0.0 9-1495058
2.6 9-6672078 9-143032 7-9450458 9-1527901 9-8072551
3.9 9-9426729 9-9266436
5.2 9-10797077 9-9910808
6.5 9-10982037
7.8 9-11337048
9.1 9-11514852
10.4 9-11644543
13.1 9-11919753
15.7 9-12352567
18.3 9-13010763
19.6 9-13485315
20.9 9-13628313 9-13738811
22.2 15-5061743 9-14176340
24.9 9-14801606
26.1 9-14970077
27.4 9-14987810 9-15127029
28.7 9-15497556 9-15265911
30.0 9-16293620
31.3 9-16309515
32.6 9-16436611
33.9 1-33374850
35.2 1-33355454
36.5 9-16868255
37.8 9-16900682
39.1 9-17010151
40.4 9-17200878
41.7 9-17458642
43.0 9-17642421
44.3 9-17801624
45.6 9-17827698
46.9 9-19032207 9-18845069
50.9 9-19754517
52.2 9-19833926
53.5 9-20228917
54.8 9-20611998
56.1 9-20956575
57.4 9-20826093
0.0 9-1047665 9-1946910 9-2644384
1.3 6-14958505 9-7302472 9-8092747
2.6 9-8797092 6-14901452
3.9 9-8838397
5.2 9-9150643
6.5 9-9142567 9-10201943
7.8 9-11349939
9.1 5-13447502
10.4 9-11816547
11.7 9-11999102
13.0 9-12024075
14.3 9-12839951
15.6 9-13121989
16.9 9-13298857
18.2 9-13611893
19.5 9-14108352
20.8 9-14107931
22.1 9-14200927 9-14115626
23.4 9-14703265
24.7 9-14790668
26.0 9-14954830
26.6 9-14797235
27.3 9-15521501
28.6 9-15158315
29.9 9-16294894
31.2 9-16597132
32.5 1-33354083
33.8 9-16747320
35.1 9-17386156
36.4 9-17599920
37.7 9-17907700
39.0 9-17949881
40.3 9-18133254
41.6 9-18164210
42.9 9-18177182
44.2 9-18214926
45.4 9-18363213
46.7 9-18377629
48.0 9-19252944
50.7 1-11659301
53.3 9-20832441 9-21325820 9-21282336
55.9 9-22032363
58.6 9-23346616 9-22438784
Full_female
End_female
Beginning_male
Full_male

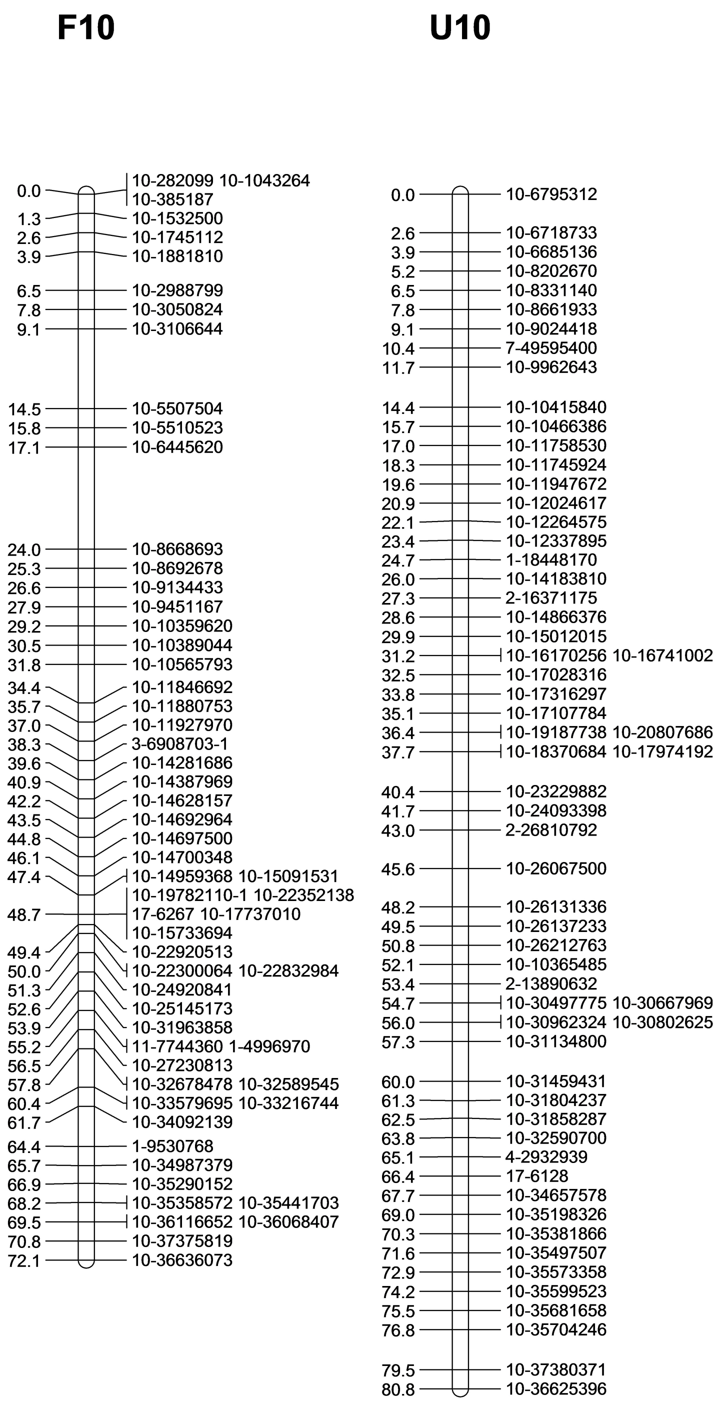
F10
U10
0.0 10-282099 10-1043264
10-385187
1.3 10-1532500
2.6 10-1745112
3.9 10-1881810
6.5 10-2988799
7.8 10-3050824
9.1 10-3106644
14.5 10-5507504
15.8 10-5510523
17.1 10-6445620
24.0 10-8668693
25.3 10-8692678
26.6 10-9134433
27.9 10-9451167
29.2 10-10359620
30.5 10-10389044
31.8 10-10565793
34.4 10-11846692
35.7 10-11880753
37.0 10-11927970
38.3 3-6908703-1
39.6 10-14281686
40.9 10-14387969
42.2 10-14628157
43.5 10-14692964
44.8 10-14697500
46.1 10-14700348
47.4 10-14959368 10-15091531
10-19782110-1 10-22352138
48.7 17-6267 10-17737010
10-15733694
49.4 10-22920513
50.0 10-22300064 10-22832984
51.3 10-24920841
52.6 10-25145173
53.9 10-31963858
55.2 11-7744360 1-4996970
56.5 10-27230813
57.8 10-32678478 10-32589545
60.4 10-33579695 10-33216744
61.7 10-34092139
64.4 1-9530768
65.7 10-34987379
66.9 10-35290152
68.2 10-35358572 10-35441703
69.5 10-36116652 10-36068407
70.8 10-37375819
72.1 10-36636073
0.0 10-6795312
2.6 10-6718733
3.9 10-6685136
5.2 10-8202670
6.5 10-8331140
7.8 10-8661933
9.1 10-9024418
10.4 7-49595400
11.7 10-9962643
14.4 10-10415840
15.7 10-10466386
17.0 10-11758530
18.3 10-11745924
19.6 10-11947672
20.9 10-12024617
22.1 10-12264575
23.4 10-12337895
24.7 1-18448170
26.0 10-14183810
27.3 2-16371175
28.6 10-14866376
29.9 10-15012015
31.2 10-16170256 10-16741002
32.5 10-17028316
33.8 10-17316297
35.1 10-17107784
36.4 10-19187738 10-20807686
37.7 10-18370684 10-17974192
40.4 10-23229882
41.7 10-24093398
43.0 2-26810792
45.6 10-26067500
48.2 10-26131336
49.5 10-26137233
50.8 10-26212763
52.1 10-10365485
53.4 2-13890632
54.7 10-30497775 10-30667969
56.0 10-30962324 10-30802625
57.3 10-31134800
60.0 10-31459431
61.3 10-31804237
62.5 10-31858287
63.8 10-32590700
65.1 4-2932939
66.4 17-6128
67.7 10-34657578
69.0 10-35198326
70.3 10-35381866
71.6 10-35497507
72.9 10-35573358
74.2 10-35599523
75.5 10-35681658
76.8 10-35704246
79.5 10-37380371
80.8 10-36625396

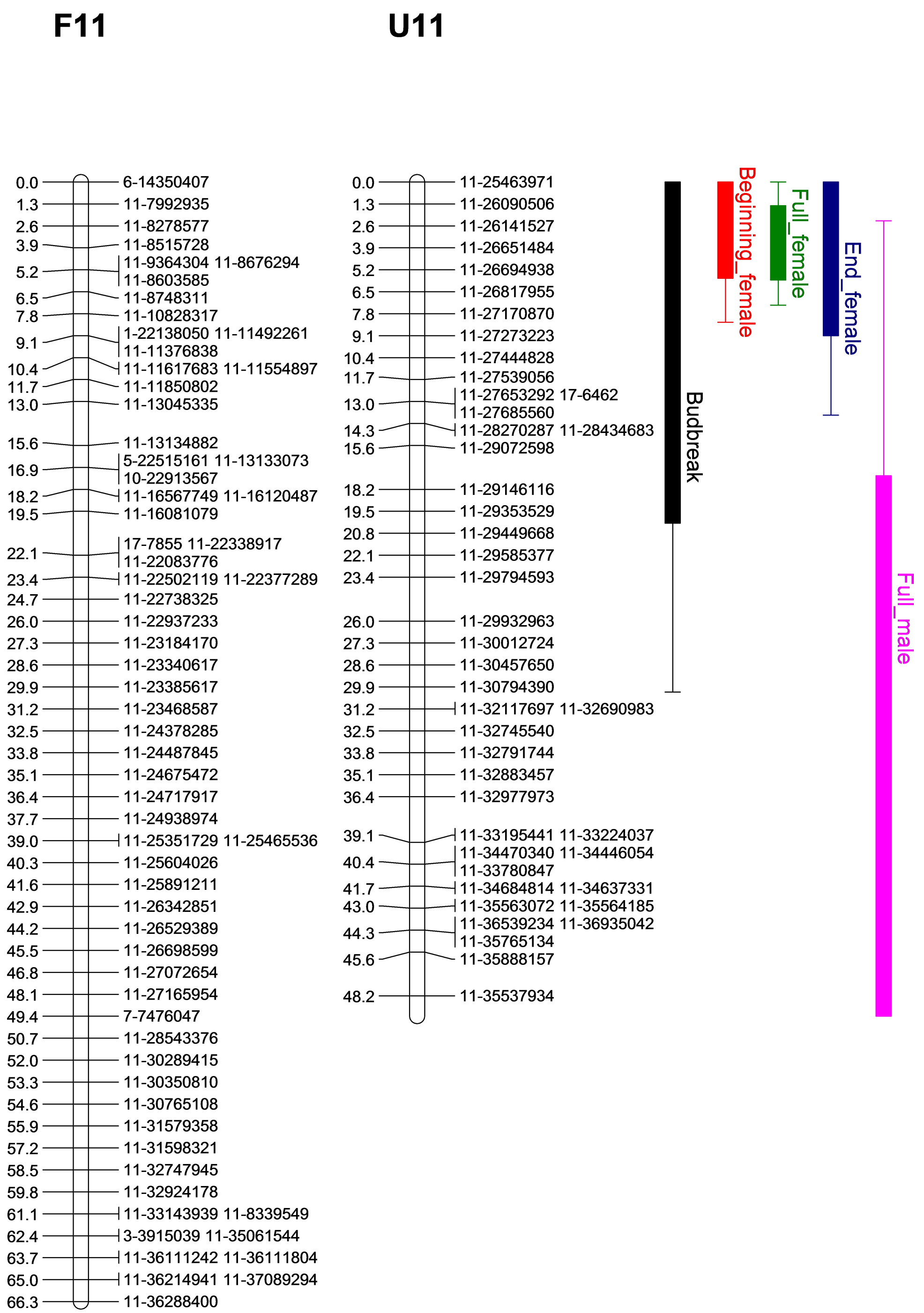
F11
U11
0.0 6-14350407
1.3 11-7992935
2.6 11-8278577
3.9 11-8515728
5.2 11-9364304 11-8676294
11-8603585
6.5 11-8748311
7.8 11-10828317
9.1 1-22138050 11-11492261
11-11376838
10.4 11-11617683 11-11554897
11.7 11-11850802
13.0 11-13045335
15.6 11-13134882
16.9 5-22515161 11-13133073
10-22913567
18.2 11-16567749 11-16120487
19.5 11-16081079
22.1 17-7855 11-22338917
11-22083776
23.4 11-22502119 11-22377289
24.7 11-22738325
26.0 11-22937233
27.3 11-23184170
28.6 11-23340617
29.9 11-23385617
31.2 11-23468587
32.5 11-24378285
33.8 11-24487845
35.1 11-24675472
36.4 11-24717917
37.7 11-24938974
39.0 11-25351729 11-25465536
40.3 11-25604026
41.6 11-25891211
42.9 11-26342851
44.2 11-26529389
45.5 11-26698599
46.8 11-27072654
48.1 11-27165954
49.4 7-7476047
50.7 11-28543376
52.0 11-30289415
53.3 11-30350810
54.6 11-30765108
55.9 11-31579358
57.2 11-31598321
58.5 11-32747945
59.8 11-32924178
61.1 11-33143939 11-8339549
62.4 3-3915039 11-35061544
63.7 11-36111242 11-36111804
65.0 11-36214941 11-37089294
66.3 11-36288400
0.0 11-25463971
1.3 11-26090506
2.6 11-26141527
3.9 11-26651484
5.2 11-26694938
6.5 11-26817955
7.8 11-27170870
9.1 11-27273223
10.4 11-27444828
11.7 11-27539056
13.0 11-27653292 17-6462
11-27685560
14.3 11-28270287 11-28434683
15.6 11-29072598
18.2 11-29146116
19.5 11-29353529
20.8 11-29449668
22.1 11-29585377
23.4 11-29794593
26.0 11-29932963
27.3 11-30012724
28.6 11-30457650
29.9 11-30794390
31.2 11-32117697 11-32690983
32.5 11-32745540
33.8 11-32791744
35.1 11-32883457
36.4 11-32977973
39.1 11-33195441 11-33224037
40.4 11-34470340 11-34446054
11-33780847
41.7 11-34684814 11-34637331
43.0 11-35563072 11-35564185
44.3 11-36539234 11-36935042
11-35765134
45.6 11-35888157
48.2 11-35537934
Budbreak
Beginning_female
Full_female
End_female
Full_male

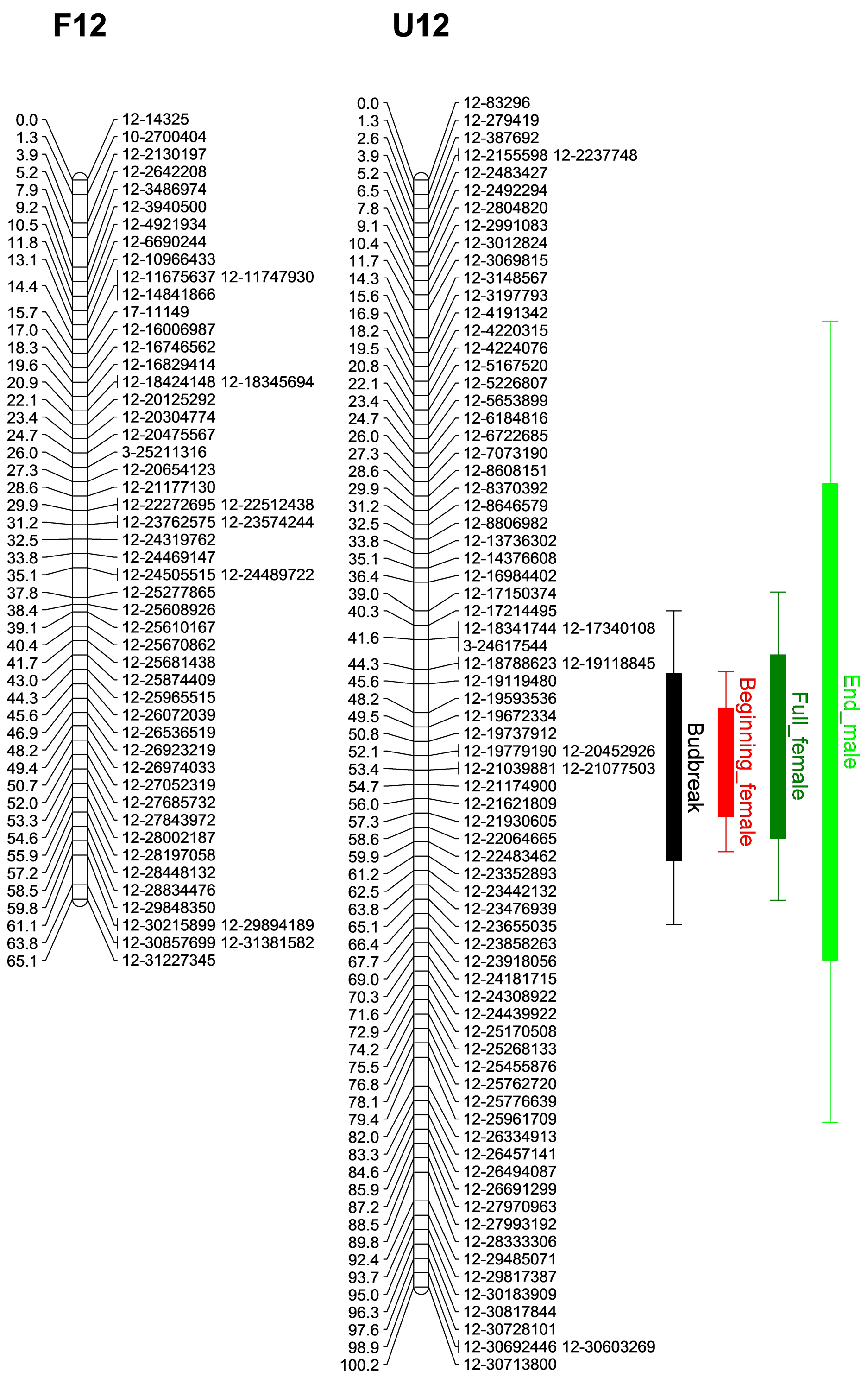
F12
U12
0.0 12-14325
1.3 10-2700404
3.9 12-2130197
5.2 12-2642208
7.9 12-3486974
9.2 12-3940500
10.5 12-4921934
11.8 12-6690244
13.1 12-10966433
14.4 12-11675637 12-11747930
12-14841866
15.7 17-11149
17.0 12-16006987
18.3 12-16746562
19.6 12-16829414
20.9 12-18424148 12-18345694
22.1 12-20125292
23.4 12-20304774
24.7 12-20475567
26.0 3-25211316
27.3 12-20654123
28.6 12-21177130
29.9 12-22272695 12-22512438
31.2 12-23762575 12-23574244
32.5 12-24319762
33.8 12-24469147
35.1 12-24505515 12-24489722
37.8 12-25277865
38.4 12-25608926
39.1 12-25610167
40.4 12-25670862
41.7 12-25681438
43.0 12-25874409
44.3 12-25965515
45.6 12-26072039
46.9 12-26536519
48.2 12-26923219
49.4 12-26974033
50.7 12-27052319
52.0 12-27685732
53.3 12-27843972
54.6 12-28002187
55.9 12-28197058
57.2 12-28448132
58.5 12-28834476
59.8 12-29848350
61.1 12-30215899 12-29894189
63.8 12-30857699 12-31381582
65.1 12-31227345
0.0 12-83296
1.3 12-279419
2.6 12-387692
3.9 12-2155598 12-2237748
5.2 12-2483427
6.5 12-2492294
7.8 12-2804820
9.1 12-2991083
10.4 12-3012824
11.7 12-3069815
14.3 12-3148567
15.6 12-3197793
16.9 12-4191342
18.2 12-4220315
19.5 12-4224076
20.8 12-5167520
22.1 12-5226807
23.4 12-5653899
24.7 12-6184816
26.0 12-6722685
27.3 12-7073190
28.6 12-8608151
29.9 12-8370392
31.2 12-8646579
32.5 12-8806982
33.8 12-13736302
35.1 12-14376608
36.4 12-16984402
39.0 12-17150374
40.3 12-17214495
41.6 12-18341744 12-17340108
3-24617544
44.3 12-18788623 12-19118845
45.6 12-19119480
48.2 12-19593536
49.5 12-19672334
50.8 12-19737912
52.1 12-19779190 12-20452926
53.4 12-21039881 12-21077503
54.7 12-21174900
56.0 12-21621809
57.3 12-21930605
58.6 12-22064665
59.9 12-22483462
61.2 12-23352893
62.5 12-23442132
63.8 12-23476939
65.1 12-23655035
66.4 12-23858263
67.7 12-23918056
69.0 12-24181715
70.3 12-24308922
71.6 12-24439922
72.9 12-25170508
74.2 12-25268133
75.5 12-25455876
76.8 12-25762720
78.1 12-25776639
79.4 12-25961709
82.0 12-26334913
83.3 12-26457141
84.6 12-26494087
85.9 12-26691299
87.2 12-27970963
88.5 12-27993192
89.8 12-28333306
92.4 12-29485071
93.7 12-29817387
95.0 12-30183909
96.3 12-30817844
97.6 12-30728101
98.9 12-30692446 12-30603269
100.2 12-30713800
Budbreak
Beginning_female
Full_female
End_male

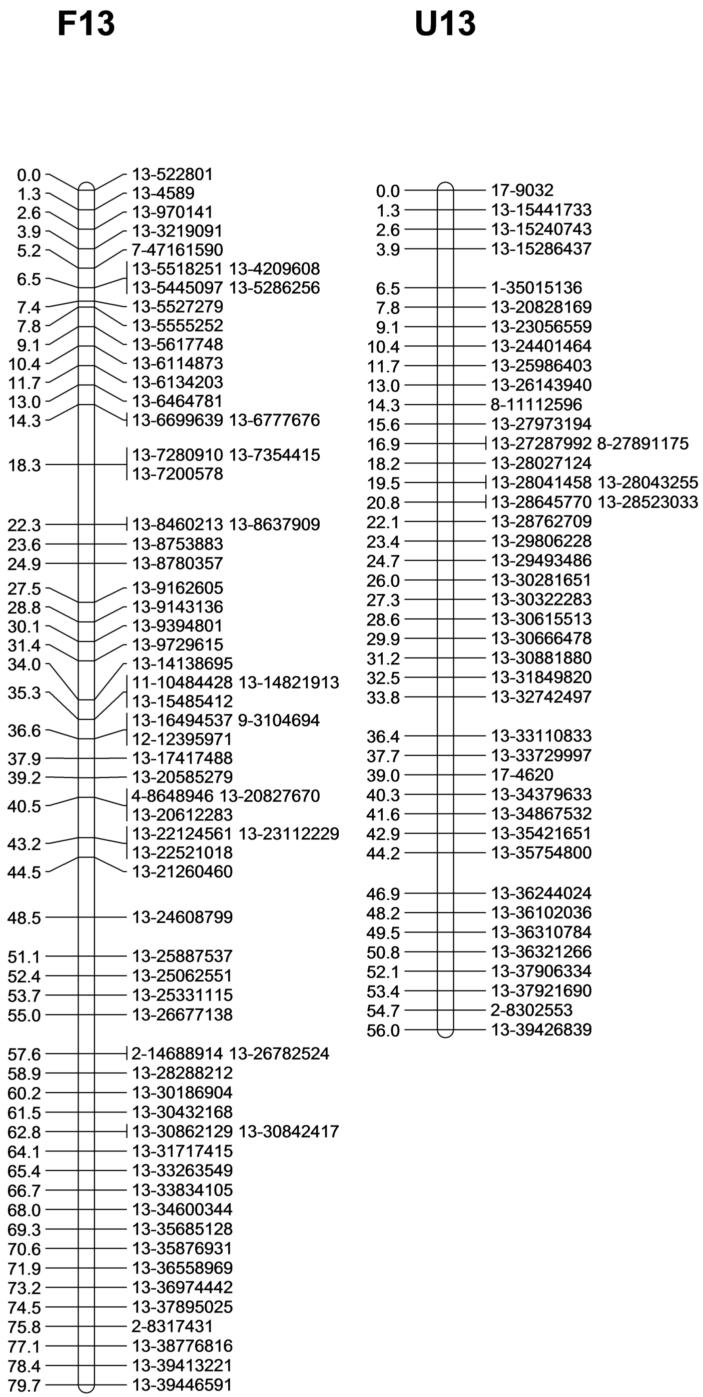
F13
0.0 13-522801
1.3 13-4589
2.6 13-970141
3.9 13-3219091
5.2 7-47161590
6.5 13-5518251 13-4209608 13-5445097 13-5286256
7.4 13-5527279
7.8 13-5555252
9.1 13-5617748
10.4 13-6114873
11.7 13-6134203
13.0 13-6464781
14.3 13-6699639 13-6777676
18.3 13-7280910 13-7354415 13-7200578
22.3 13-8460213 13-8637909
23.6 13-8753883
24.9 13-8780357
27.5 13-9162605
28.8 13-9143136
30.1 13-9394801
31.4 13-9729615
34.0 13-14138695
35.3 11-10484428 13-14821913 13-15485412
36.6 13-16494537 9-3104694 12-12395971
37.9 13-17417488
39.2 13-20585279
40.5 4-8648946 13-20827670 13-20612283
43.2 13-22124561 13-23112229 13-22521018
44.5 13-21260460
48.5 13-24608799
51.1 13-25887537
52.4 13-25062551
53.7 13-25331115
55.0 13-26677138
57.6 2-14688914 13-26782524
58.9 13-28288212
60.2 13-30186904
61.5 13-30432168
62.8 13-30862129 13-30842417
64.1 13-31717415
65.4 13-33263549
66.7 13-33834105
68.0 13-34600344
69.3 13-35685128
70.6 13-35876931
71.9 13-36558969
73.2 13-36974442
74.5 13-37895025
75.8 2-8317431
77.1 13-38776816
78.4 13-39413221
79.7 13-39446591
U13
0.0 17-9032
1.3 13-15441733
2.6 13-15240743
3.9 13-15286437
6.5 1-35015136
7.8 13-20828169
9.1 13-23056559
10.4 13-24401464
11.7 13-25986403
13.0 13-26143940
14.3 8-11112596
15.6 13-27973194
16.9 13-27287992 8-27891175
18.2 13-28027124
19.5 13-28041458 13-28043255
20.8 13-28645770 13-28523033
22.1 13-28762709
23.4 13-29806228
24.7 13-29493486
26.0 13-30281651
27.3 13-30322283
28.6 13-30615513
29.9 13-30666478
31.2 13-30881880
32.5 13-31849820
33.8 13-32742497
36.4 13-33110833
37.7 13-33729997
39.0 17-4620
40.3 13-34379633
41.6 13-34867532
42.9 13-35421651
44.2 13-35754800
46.9 13-36244024
48.2 13-36102036
49.5 13-36310784
50.8 13-36321266
52.1 13-37906334
53.4 13-37921690
54.7 2-8302553
56.0 13-39426839

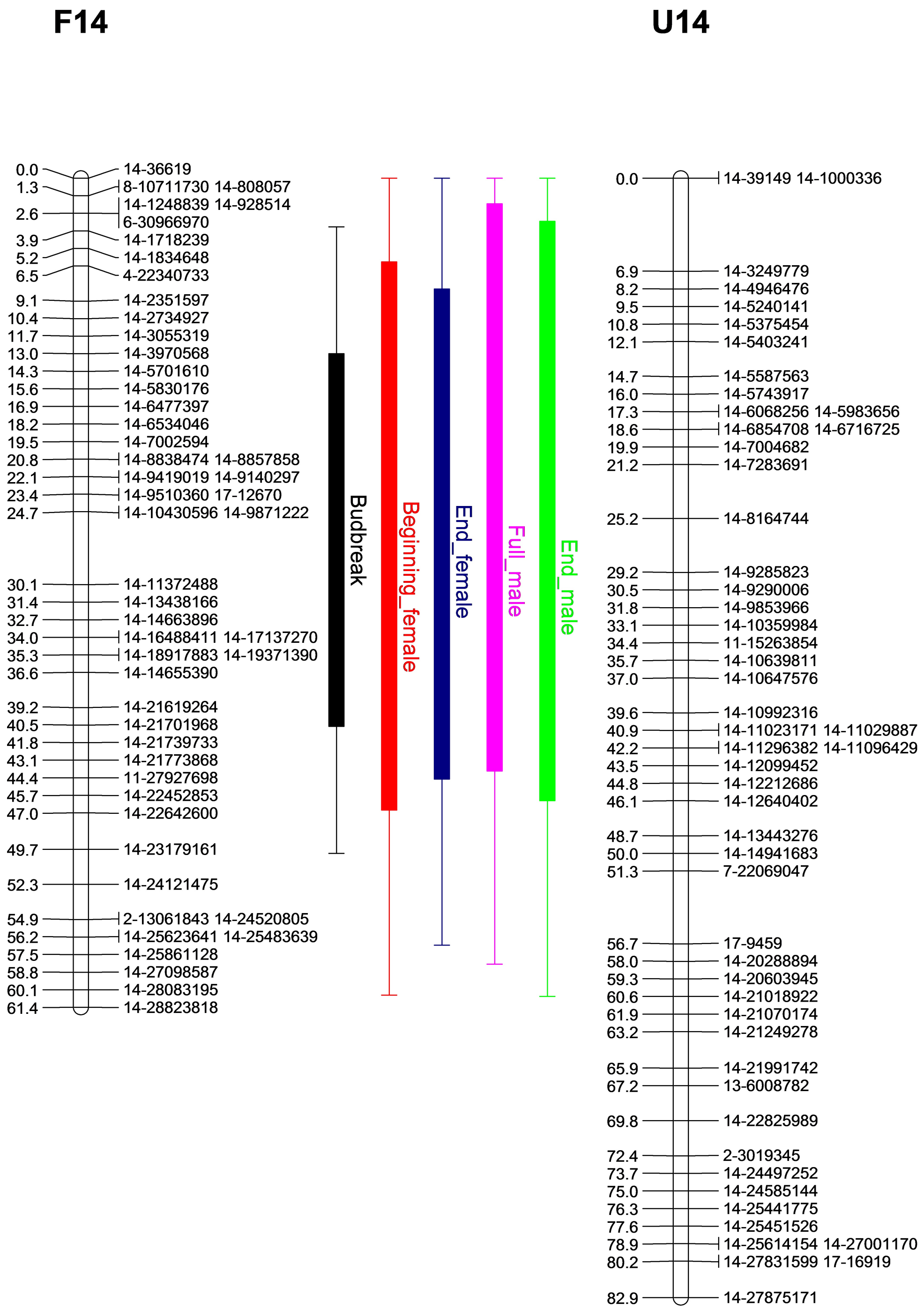
F14
U14
0.0 14-36619
1.3 8-10711730 14-808057
2.6 14-1248839 14-928514 6-30966970
3.9 14-1718239
5.2 14-1834648
6.5 4-22340733
9.1 14-2351597
10.4 14-2734927
11.7 14-3055319
13.0 14-3970568
14.3 14-5701610
15.6 14-5830176
16.9 14-6477397
18.2 14-6534046
19.5 14-7002594
20.8 14-8838474 14-8857858
22.1 14-9419019 14-9140297
23.4 14-9510360 17-12670
24.7 14-10430596 14-9871222
30.1 14-11372488
31.4 14-13438166
32.7 14-14663896
34.0 14-16488411 14-17137270
35.3 14-18917883 14-19371390
36.6 14-14655390
39.2 14-21619264
40.5 14-21701968
41.8 14-21739733
43.1 14-21773868
44.4 11-27927698
45.7 14-22452853
47.0 14-22642600
49.7 14-23179161
52.3 14-24121475
54.9 2-13061843 14-24520805
56.2 14-25623641 14-25483639
57.5 14-25861128
58.8 14-27098587
60.1 14-28083195
61.4 14-28823818
Budbreak
Beginning_female
End_female
Full_male
End_male
0.0 14-39149 14-1000336
6.9 14-3249779
8.2 14-4946476
9.5 14-5240141
10.8 14-5375454
12.1 14-5403241
14.7 14-5587563
16.0 14-5743917
17.3 14-6068256 14-5983656
18.6 14-6854708 14-6716725
19.9 14-7004682
21.2 14-7283691
25.2 14-8164744
29.2 14-9285823
30.5 14-9290006
31.8 14-9853966
33.1 14-10359984
34.4 11-15263854
35.7 14-10639811
37.0 14-10647576
39.6 14-10992316
40.9 14-11023171 14-11029887
42.2 14-11296382 14-11096429
43.5 14-12099452
44.8 14-12212686
46.1 14-12640402
48.7 14-13443276
50.0 14-14941683
51.3 7-22069047
56.7 17-9459
58.0 14-20288894
59.3 14-20603945
60.6 14-21018922
61.9 14-21070174
63.2 14-21249278
65.9 14-21991742
67.2 13-6008782
69.8 14-22825989
72.4 2-3019345
73.7 14-24497252
75.0 14-24585144
76.3 14-25441775
77.6 14-25451526
78.9 14-25614154 14-27001170
80.2 14-27831599 17-16919
82.9 14-27875171

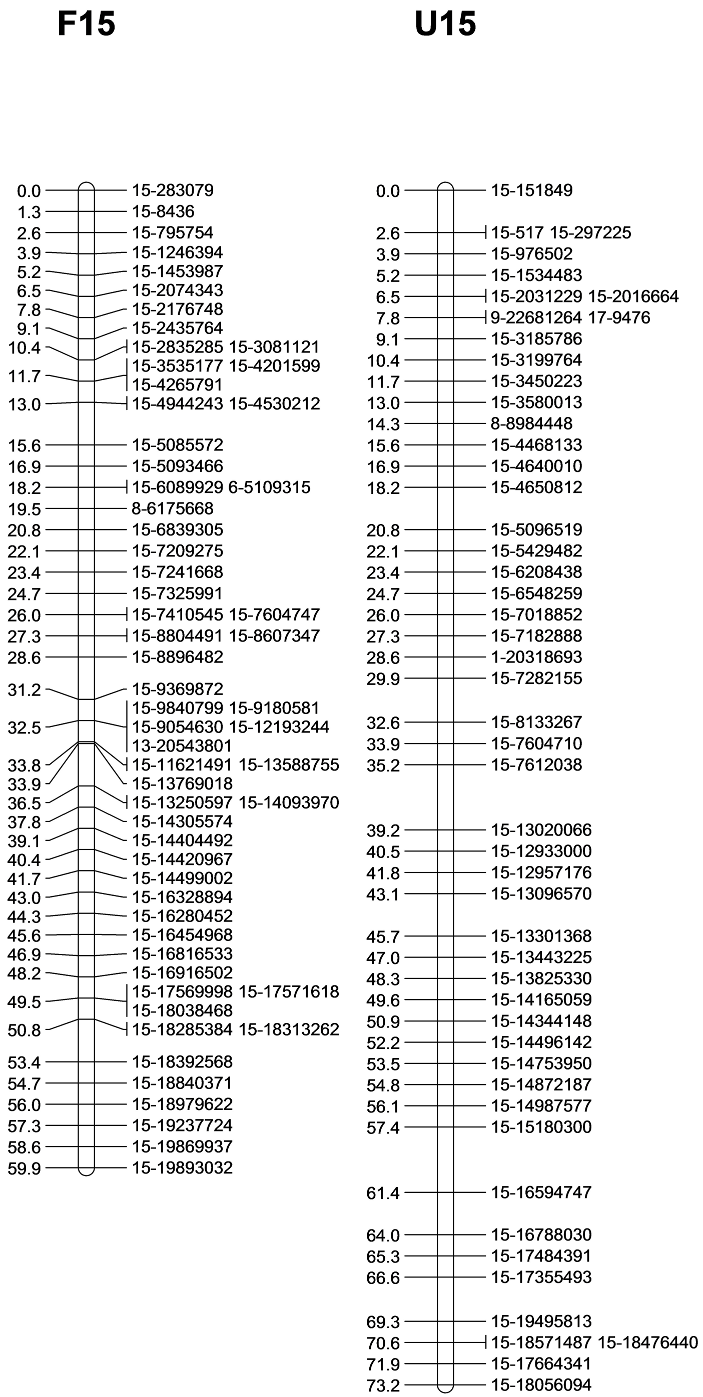
F15
0.0 15-283079
1.3 15-8436
2.6 15-795754
3.9 15-1246394
5.2 15-1453987
6.5 15-2074343
7.8 15-2176748
9.1 15-2435764
10.4 15-2835285 15-3081121
11.7 15-3535177 15-4201599 15-4265791
13.0 15-4944243 15-4530212
15.6 15-5085572
16.9 15-5093466
18.2 15-6089929 6-5109315
19.5 8-6175668
20.8 15-6839305
22.1 15-7209275
23.4 15-7241668
24.7 15-7325991
26.0 15-7410545 15-7604747
27.3 15-8804491 15-8607347
28.6 15-8896482
31.2 15-9369872
32.5 15-9840799 15-9180581 15-9054630 15-12193244 13-20543801
33.8 15-11621491 15-13588755
33.9 15-13769018
36.5 15-13250597 15-14093970
37.8 15-14305574
39.1 15-14404492
40.4 15-14420967
41.7 15-14499002
43.0 15-16328894
44.3 15-16280452
45.6 15-16454968
46.9 15-16816533
48.2 15-16916502
49.5 15-17569998 15-17571618 15-18038468
50.8 15-18285384 15-18313262
53.4 15-18392568
54.7 15-18840371
56.0 15-18979622
57.3 15-19237724
58.6 15-19869937
59.9 15-19893032
U15
0.0 15-151849
2.6 15-517 15-297225
3.9 15-976502
5.2 15-1534483
6.5 15-2031229 15-2016664
7.8 9-22681264 17-9476
9.1 15-3185786
10.4 15-3199764
11.7 15-3450223
13.0 15-3580013
14.3 8-8984448
15.6 15-4468133
16.9 15-4640010
18.2 15-4650812
20.8 15-5096519
22.1 15-5429482
23.4 15-6208438
24.7 15-6548259
26.0 15-7018852
27.3 15-7182888
28.6 1-20318693
29.9 15-7282155
32.6 15-8133267
33.9 15-7604710
35.2 15-7612038
39.2 15-13020066
40.5 15-12933000
41.8 15-12957176
43.1 15-13096570
45.7 15-13301368
47.0 15-13443225
48.3 15-13825330
49.6 15-14165059
50.9 15-14344148
52.2 15-14496142
53.5 15-14753950
54.8 15-14872187
56.1 15-14987577
57.4 15-15180300
61.4 15-16594747
64.0 15-16788030
65.3 15-17484391
66.6 15-17355493
69.3 15-19495813
70.6 15-18571487 15-18476440
71.9 15-17664341
73.2 15-18056094

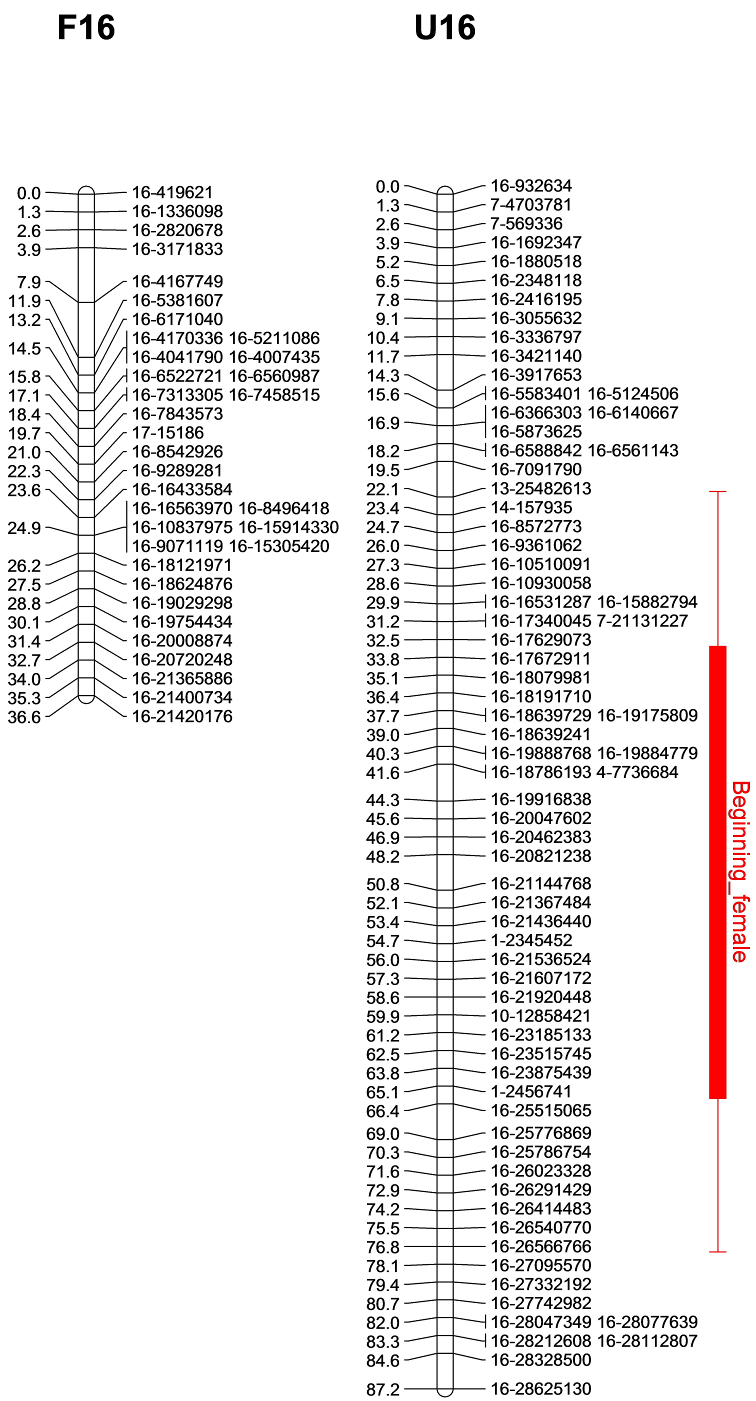
F16
U16
0.0 16-419621
1.3 16-1336098
2.6 16-2820678
3.9 16-3171833
7.9 16-4167749
11.9 16-5381607
13.2 16-6171040
14.5 16-4170336 16-5211086
16-4041790 16-4007435
15.8 16-6522721 16-6560987
17.1 16-7313305 16-7458515
18.4 16-7843573
19.7 17-15186
21.0 16-8542926
22.3 16-9289281
23.6 16-16433584
24.9 16-16563970 16-8496418
16-10837975 16-15914330
16-9071119 16-15305420
26.2 16-18121971
27.5 16-18624876
28.8 16-19029298
30.1 16-19754434
31.4 16-20008874
32.7 16-20720248
34.0 16-21365886
35.3 16-21400734
36.6 16-21420176
0.0 16-932634
1.3 7-4703781
2.6 7-569336
3.9 16-1692347
5.2 16-1880518
6.5 16-2348118
7.8 16-2416195
9.1 16-3055632
10.4 16-3336797
11.7 16-3421140
14.3 16-3917653
15.6 16-5583401 16-5124506
16.9 16-6366303 16-6140667
16-5873625
18.2 16-6588842 16-6561143
19.5 16-7091790
22.1 13-25482613
23.4 14-157935
24.7 16-8572773
26.0 16-9361062
27.3 16-10510091
28.6 16-10930058
29.9 16-16531287 16-15882794
31.2 16-17340045 7-21131227
32.5 16-17629073
33.8 16-17672911
35.1 16-18079981
36.4 16-18191710
37.7 16-18639729 16-19175809
39.0 16-18639241
40.3 16-19888768 16-19884779
41.6 16-18786193 4-7736684
44.3 16-19916838
45.6 16-20047602
46.9 16-20462383
48.2 16-20821238
50.8 16-21144768
52.1 16-21367484
53.4 16-21436440
54.7 1-2345452
56.0 16-21536524
57.3 16-21607172
58.6 16-21920448
59.9 10-12858421
61.2 16-23185133
62.5 16-23515745
63.8 16-23875439
65.1 1-2456741
66.4 16-25515065
69.0 16-25776869
70.3 16-25786754
71.6 16-26023328
72.9 16-26291429
74.2 16-26414483
75.5 16-26540770
76.8 16-26566766
78.1 16-27095570
79.4 16-27332192
80.7 16-27742982
82.0 16-28047349 16-28077639
83.3 16-28212608 16-28112807
84.6 16-28328500
87.2 16-28625130
Beginning_female
