# Supplementary material for: Association and linkage mapping to unravel genetic architecture of phenological traits and lateral bearing in Persian walnut (Juglans regia L.)
Source: BMC Genomics. 2020 Mar 4;21:203. doi: 10.1186/s12864-020-6616-y (PMC7057608; doi:10.1186/s12864-020-6616-y)
Supplement: Supplementary file 8 — Additional file 8: Figure S1. Scatter plots showing the two-year data related to phenological traits in Julian days for the 170 accessions of the GWAS panel. [file 12864_2020_6616_MOESM8_ESM.pdf]

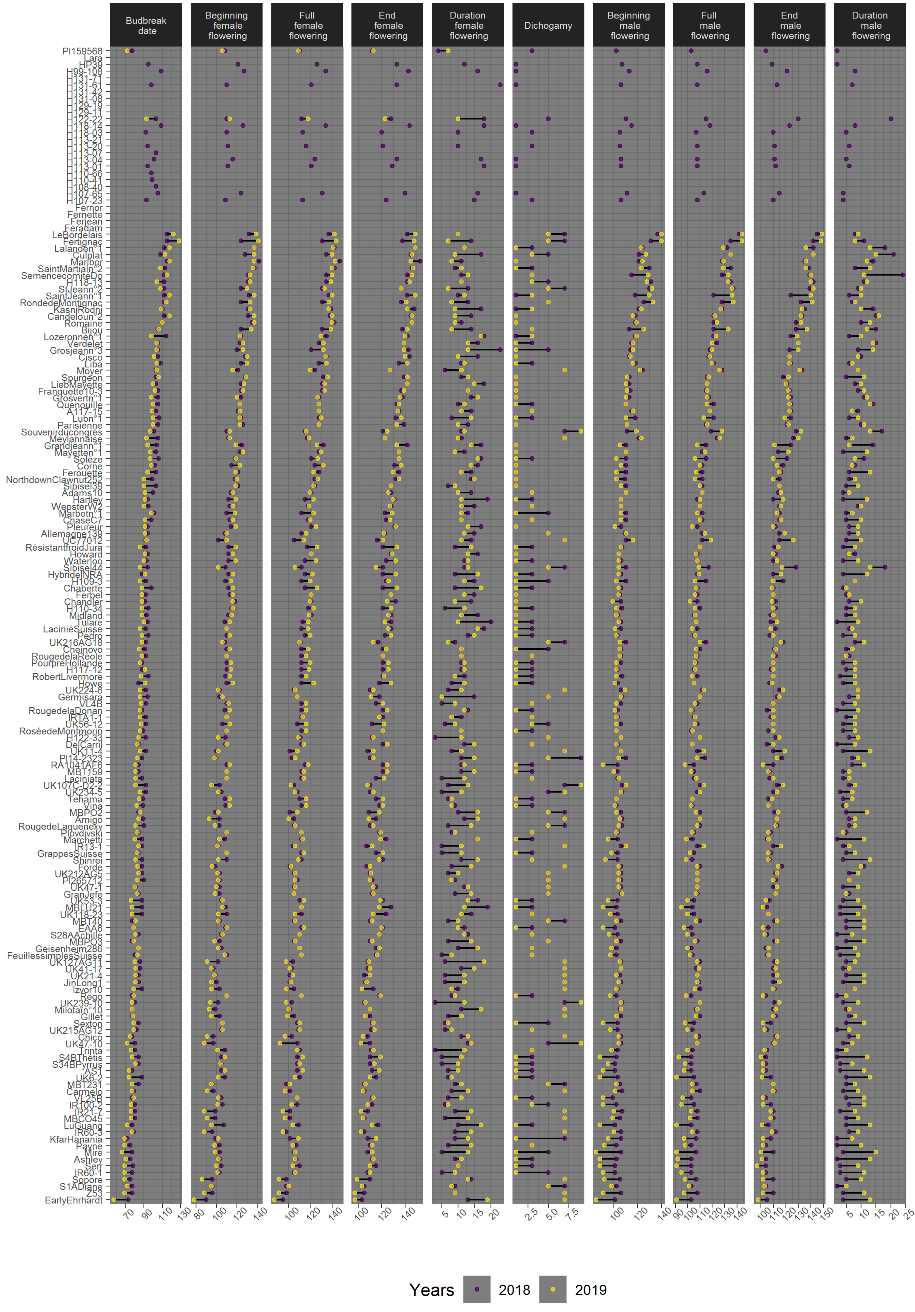
Budbreak date
Beginning female flowering
Full female flowering
End female flowering
Duration female flowering
Dichogamy
Beginning male flowering
Full male flowering
End male flowering
Duration male flowering
70 90 110 130
80 100 120 140
100 120 140
100 120 140
5 10 15 20
2.5 5.0 7.5
100 120 140
90 100 110 120 130 140
100 110 120 130 140 150
5 10 15 20 25
Years
2018
2019
